# Design, Synthesis, and Structure–Activity Relationship Study of Potent MAPK11 Inhibitors

**DOI:** 10.3390/molecules27010203

**Published:** 2021-12-29

**Authors:** Mengdie Gong, Mingyan Tu, Hongxia Sun, Lu Li, Lili Zhu, Honglin Li, Zhenjiang Zhao, Shiliang Li

**Affiliations:** 1 Shanghai Key Laboratory of New Drug Design, School of Pharmacy, East China University of Science & Technology, Shanghai 200237, China; mdgong1@163.com (M.G.); tumingyan@gmail.com (M.T.); shx7982@163.com (H.S.); lilu_dws_llm@163.com (L.L.); zhulfl@ecust.edu.cn (L.Z.); hlli@ecust.edu.cn (H.L.); 2 Jiangzhong Pharmaceutical Co., Ltd., Nanchang 330096, China

**Keywords:** MAPK11, Huntington’s disease, mHTT protein, inhibitors

## Abstract

Huntington’s disease (HD) is a rare single-gene neurodegenerative disease, which can only be treated symptomatically. Currently, there are no approved drugs for HD on the market. Studies have found that MAPK11 can serve as a potential therapeutic target for HD. Regrettably, no MAPK11 small molecule inhibitors have been approved at present. This paper presents three series of compounds that were designed and synthesized based on the structure of skepinone-L, a known MAPK14 inhibitor. Among the synthesized compounds, **13a** and **13b**, with IC_50_ values of 6.40 nM and 4.20 nM, respectively, displayed the best inhibitory activities against MAPK11. Furthermore, the structure–activity relationship (SAR) is discussed in detail, which is constructive in optimizing the MAPK11 inhibitors for better activity and effect against HD.

## 1. Introduction

Huntington’s disease (HD) is an autosomal-dominant neurodegenerative disorder characterized by a combination of several motor, cognitive, and psychiatric symptoms. It is typically adult-onset, with irreversible progression of symptoms over 10 to 15 years [1]. All cases of HD are caused by the same mutation, CAG trinucleotide repeat amplification in the HTT gene, resulting in ubiquitous expression of the toxic mutant huntingtin (mHTT) protein [2]. Therefore, reducing the production of mHTT could be a potential treatment strategy for HD. Unfortunately, due to the lack of disease relief therapy, only supportive and symptomatic management is available at present [3].

Mitogen-activated protein kinases (MAPKs) are serine/threonine protein kinases with the ability to transform extracellular stimuli into various cellular responses. Among them, the p38MAPK family consists of four isozymes: p38α (MAPK14), p38β (MAPK11), p38γ (MAPK12), and p38δ (MAPK13) [4]. Abnormal p38MAPK signal transduction in nerve cells is linked to the pathogenesis of various neurodegenerative diseases, including Alzheimer’s disease, Parkinson’s disease, and HD [5]. Recent studies have shown that MAPK11 can indirectly regulate the level of mHTT protein by regulating the mRNA stability of the protein. In addition, knockout of MAPK11 in a knock-in HD mouse model rescued disease-relevant behavioral phenotypes significantly [6]. Collectively, these studies revealed MAPK11 as a new therapeutic target for HD.

MAPK11, also known as p38β, was described by Jiang et al. in 1996 and is encoded by the MAPK11 gene [7]. The protein consists of 364 amino acids, including a kinase domain with a T-G-Y dual phosphorylation motif, essential for its kinase activity [8]. The three-dimensional structure of MAPK11 resembles that of a typical kinase with a smaller β-sheet N-terminal domain and a larger C-terminal domain. The ATP-binding site is located between the two domains, which are linked by a single polypeptide chain [7]. In terms of their overall structure, MAPK14 and MAPK11 are highly similar. However, due to the differences in the relative orientation of the N- and C-terminal domains, the size of the ATP-binding pocket in MAPK11 is smaller in comparison to MAPK14 [9]. This size difference between the two pockets can be exploited to achieve selectivity.

**Figure 1:**
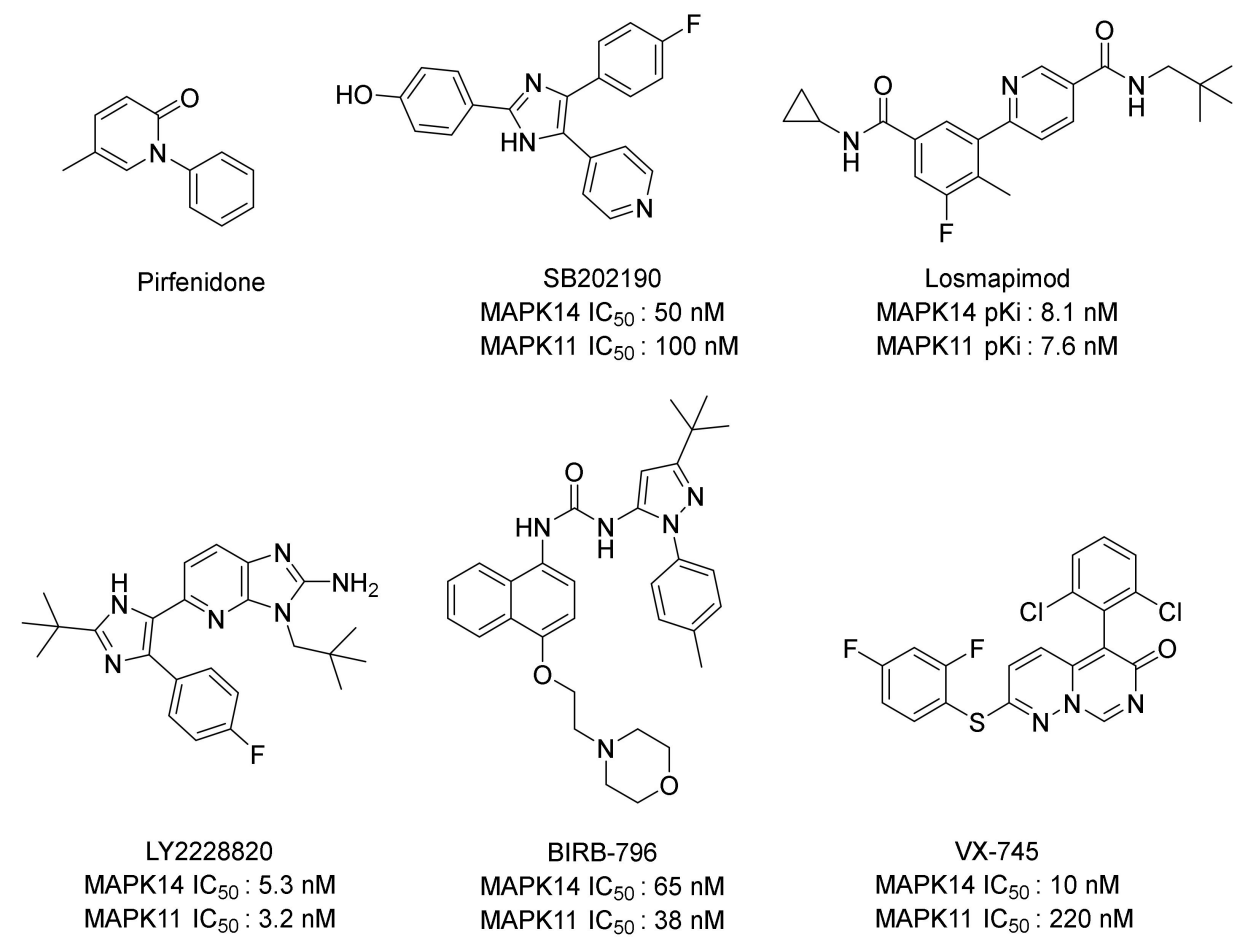
Structures of some representative p38MAPK inhibitors.

Even though there are more than 20 candidates in clinical trials, no drug (except the weak and unselective p38 inhibitor, pirfenidone) has been approved for p38MAPK yet [10]. Among these, inhibitors related to MAPK11 mainly include SB202190 [11], Losmapimod [12], LY2228820 [13], BIRB-796 [14], VX-745 [15], and so on (Figure 1). Despite being highly active and selective MAPK14 inhibitors, they show inhibitory effects on MAPK11 as well. However, no small molecule inhibitors for HD have been approved for marketing at present. Therefore, it is of great research significance to develop MAPK11 inhibitors for the treatment of HD.

**Figure 2:**
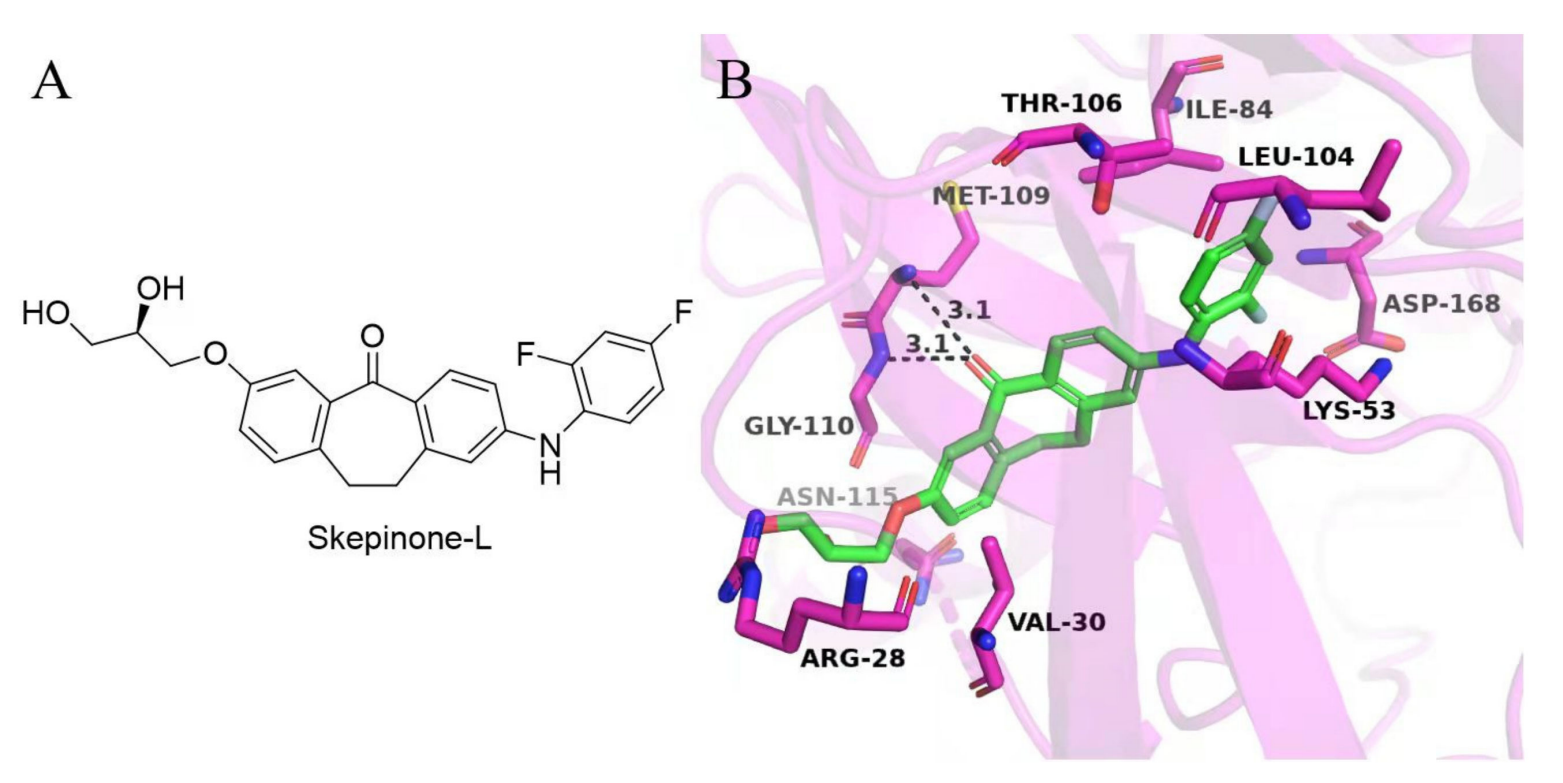
(**A**) The chemical structure of skepinone-L; (**B**) Predicted binding mode of skepinone-L with MAPK11. The structure of MAPK11 is shown in red cartoon (PDB ID: 3GC8). Skepinone-L is presented as green sticks. Key residues are shown in purple sticks and labeled. The H-bond interactions are depicted as black dashed lines.

**Figure 3:**
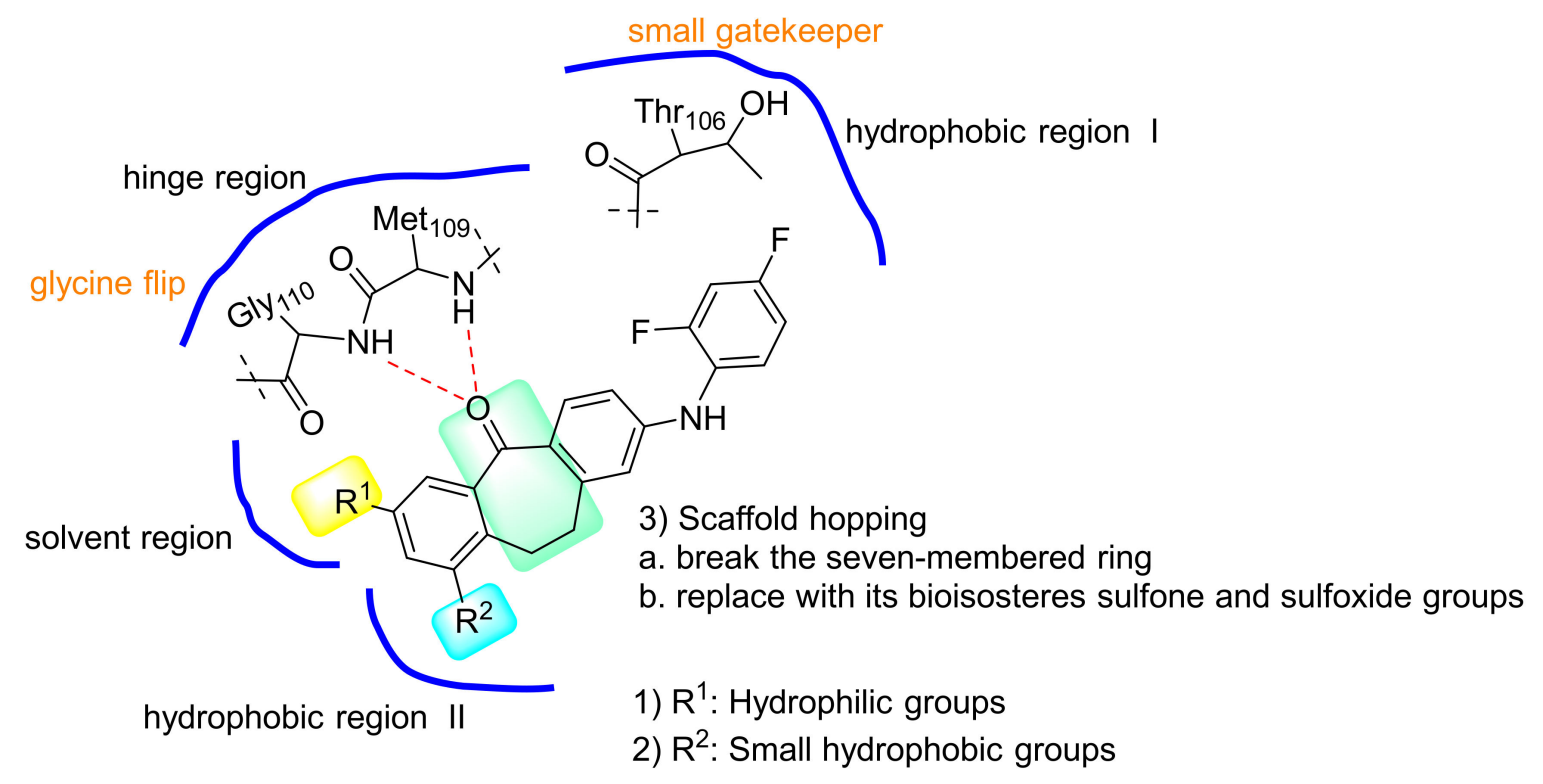
Design and optimization strategies of novel MAPK11 inhibitors.

Skepinone-L is the first ATP-competitive MAPK14 inhibitor (IC_50_ = 5 nM) showing excellent in vivo efficacy and selectivity [16]. The small gatekeeper amino acid Thr106 in the hydrophobic region I and the glycine flip in the hinge region are the key factors for it achieving selectivity [17]. As these determinants of selectivity, such as Thr106 and Gly110, are also conserved in MAPK11, we selected skepinone-L as the lead compound to design MAPK11 inhibitors (Figure 2). In this study, several modification strategies were conducted based on the diphenylcycloheptanone skeleton of skepinone-L with the expectation of discovering more novel and selective MAPK11 inhibitors. It mainly included the following: (1) modification at R^1^ with hydrophilic groups in the kinase solvent region; (2) modification at R^2^ with small hydrophobic groups in the hydrophobic region II; (3) scaffold hopping to diaryl sulfone and sulfoxide derivatives to attempt to obtain novel scaffolds (Figure 3). As a result, a series of dibenzocycloheptanone, diaryl sulfone, and sulfoxide compounds were designed and synthesized, and the biological activities against MAPK11 were evaluated.

## 2. Results and Discussion

### 2.1. Synthesis

**Scheme 1:**
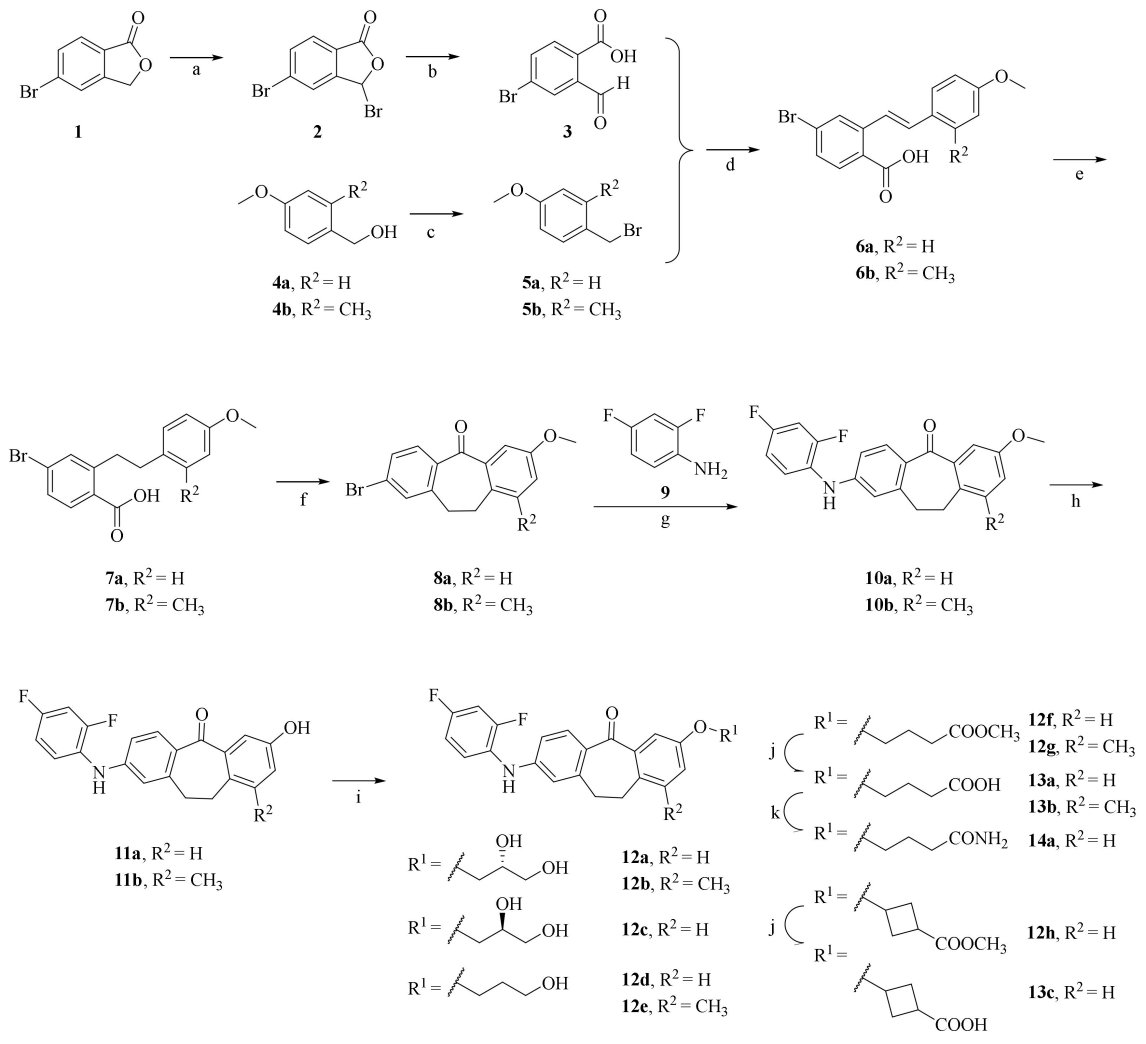
Synthesis of dibenzocycloheptanone derivatives **12**–**14** ^a^. ^a^ Reagents and conditions: (**a**) NBS, AIBN, DCE, reflux, 63.0%; (**b**) H_2_O, reflux, 63.5%; (**c**) PBr_3_, dry DCM, 98.5%; (**d**) (1) PPh_3_, toluene, reflux, 73.0%; (2) MeOH, MeONa, reflux, 85.0%; (**e**) H_2_, Pd/C, DCM, 65.0%; (**f**) (1) SOCl_2_, dry DCM, reflux; (2) AlCl_3_, r.t., 47.7%; (**g**) t-BuOK, t-BuOH, Pd(OAc)_2_, X-Phos, toluene, 110 °C, reflux, **10a**: 83.9%, **10b**: 99.5%; (**h**) HBr, CH_3_COOH, 100 °C, reflux, 61.4%; (**i**) R^1^Cl, K_2_CO_3_, DMF, 80 °C, 47.6%; (**j**) 1N aq. NaOH, MeOH, 70 °C, reflux, 43.2%; (**k**) Et_3_N, isopropyl chlorocarbonate, 28% NH_3_·H_2_O, DCM, 0 °C-r.t., 24.2%.

The general synthetic procedures for dibenzocycloheptanone derivatives **12**–**14** are described in Scheme 1. Oxidative ring-opening with N-bromo-succinimide/azoisobutyronitrile (NBS/AIBN) after a two-step reaction yielded the desired aldehyde **3** from the starting materials 5-bromophthalide **1**. The starting material 4-methoxy-2-methylbenzaldehyde was reduced to the corresponding alcohol **4b** using sodium borohydride. Then, p-methoxybenzyl bromide derivatives **5a**–**b** were obtained from the commercial reagent **4a** and the intermediate **4b** under the action of PBr_3_. The Wittig reaction, yielding the respective stilbeno-2-carboxylic acid derivatives **6a**–**b**, is the most important step of the synthesis. Reduction of the etheno-linker using hydrogen and Pd/carbon produced the corresponding saturated acid **7a**–**b**, which was cyclized by an intramolecular Friedel–Crafts reaction with SOCl_2_/AlCl_3_ to obtain 2-bromo-methoxydibenzosuberone derivatives **8a**–**b**. Further, the Buchwald–Hartwig reaction with 2,4-difluoroaniline **9** produced intermediates **10a**–**b**, which were demethylated under the action of 48%HBr/acetic acid to obtain the key intermediates **11a**–**b**. Finally, the final products **12a**, **12c**–**d**, **12f**, and intermediate **12h** were obtained by introducing different substituents from intermediate **11a**, while the final products **12b**, **12e**, and intermediate **12g** were obtained from intermediate **11b**. Furthermore, the ester hydrolysis reaction yielded the final products **13a**–**c** from **12f**–**h**, respectively, whereas the amidation reaction gave the final product **14a** from **13a**. The key factor distinguishing the compounds **12b**, **12e**, and **13b** from the compounds **12a**, **12c**–**d**, **12f**, **12h**, **13a**, **13c**, and **14a** is the introduction of a methyl group at the 9 position of the benzene ring of dibenzocycloheptanone.

**Scheme 2:**
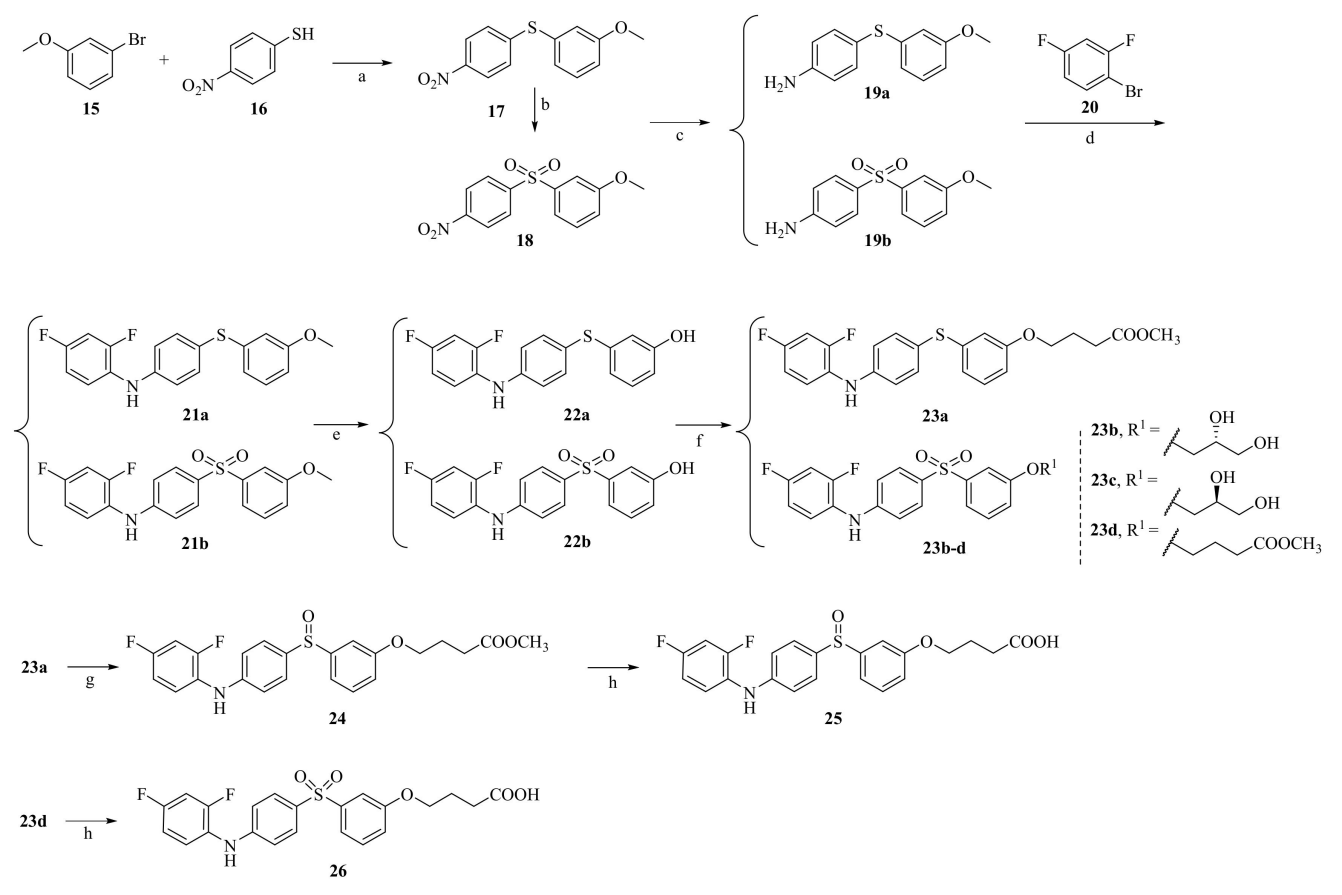
Synthesis of diaryl sulfone and sulfoxide derivatives **23**–**26** ^a^. ^a^ Reagents and conditions: (**a**) K_2_CO_3_, Cu, anhydrous DMF, N_2_, 150 °C, 35.7%; (**b**) mCPBA, DCM, 0 °C-r.t., 98.0%; (**c**) Pd/C, H_2_, MeOH, 92.6%; (**d**) Pd_2_(dba)_3_, X-phos, Cs_2_CO_3_, 90 °C, 60.7%; (**e**) CH_3_COOH, 48% HBr, 100 °C, 57.2%; (**f**) R^1^Cl, K_2_CO_3_, DMF, 80 °C, 64.7%; (**g**) 30% H_2_O_2_, p-TsOH, DCM, r.t., 78.2%; (**h**) 1N aq. NaOH, MeOH, reflux, **25**: 44.8%, **26**: 24.4%.

The diaryl sulfone and sulfoxide derivatives **23**–**26** were synthesized as presented in Scheme 2. Initially, commercially available meta-bromoanisole **15** and 4-nitrothiophenol **16** were used as starting materials to generate the intermediate **17**, followed by mCPBA oxidation with the sulfone product of **18**. Then, intermediates **17** and **18** were reduced to yield compounds **19a**–**b** under hydrogen atmosphere, which were further reacted with 2,4-difluorobromobenzene **20** subsequently using palladium-catalyzed coupling to generate the intermediates **21a**–**b**.The methyl group was then removed using hydrobromic acid, resulting in the key intermediates **22a**–**b**. Different groups were introduced to the intermediates **22a**–**b** to yield the final products **23b**–**d**, and the intermediate **23a**, which was transferred from sulfide to sulfoxide by oxidation with 30% hydrogen peroxide to produce compound **24**. Finally, compounds **24** and **23d** were subjected to ester hydrolysis reaction to yield the final products **25** and **26**, respectively.

### 2.2. Structure–Activity Relationship

Structural modification on the lead skepinone-L was carried out guided by structure-based drug design. After alignment of the amino acid residues around the ATP binding pocket of MAPK11 and MAPK14, it was found that Arg28 in MAPK11 is closer to the center of the binding pocket than Ser28 in MAPK14, which is located at the solvent-exposed region of the pocket. Then, groups with different lengths and hydrophilicity were decorated to the 7 position of the dibenzosuberone scaffold (the R^1^ group) to study their effect on the bioactivity. Compounds **12a**, **12c**–**d**, **12f**, **13a**, **13c**, and **14a** were designed and synthesized, and their inhibitory activities against MAPK11 were evaluated (shown in Table 1).

**Figure 4:**
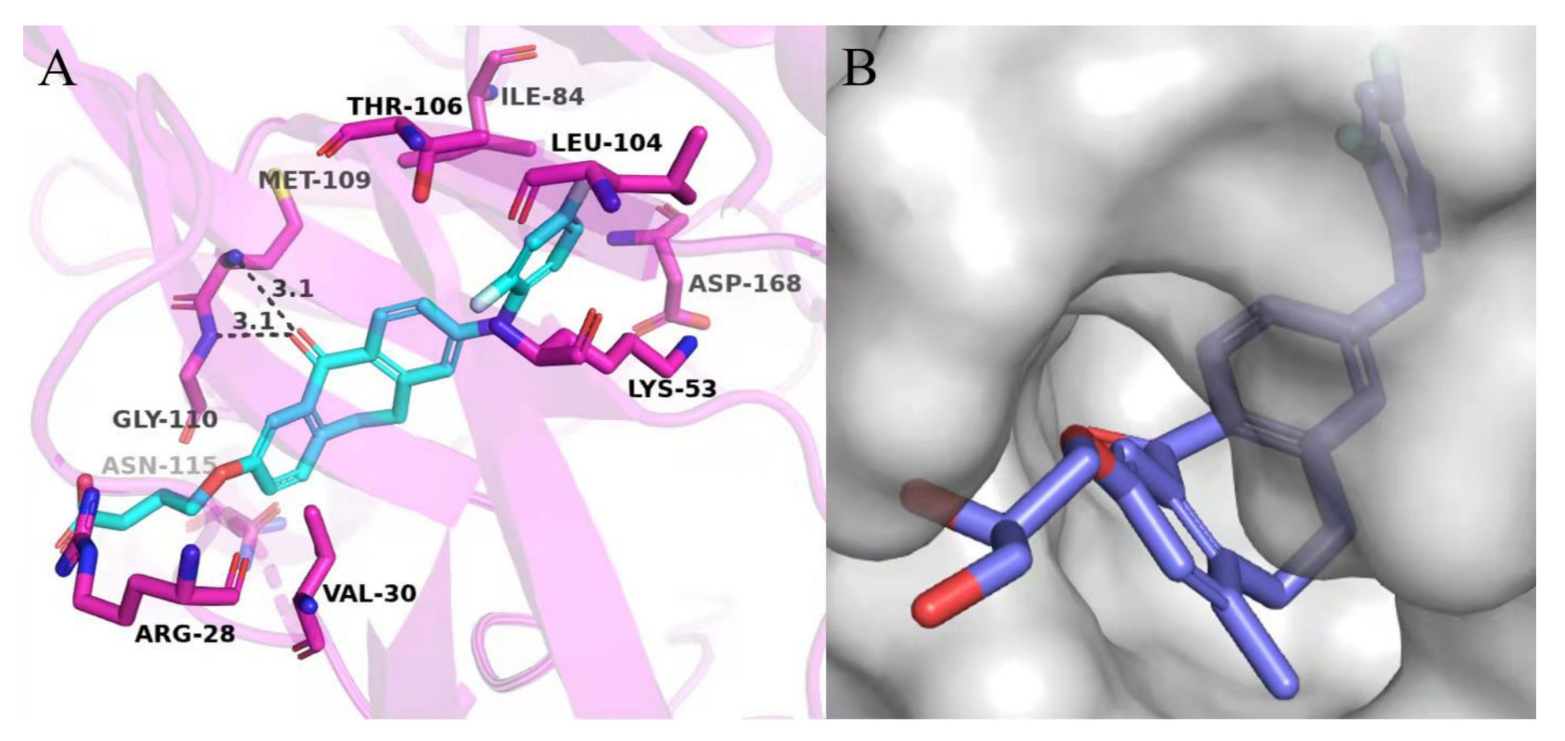
(**A**) Predicted binding mode of **13a** with MAPK11; (**B**) Surface binding model of **12b** with MAPK11. The structure of MAPK11 is shown in red cartoon (PDB ID: 3GC8), **13a** and **12b** are shown as sticks. The key residues are presented in purple sticks and labeled, and the H-bond interactions are depicted as black dashed lines.

Comparing **12c** in S configuration with the lead **12a** in R configuration, the inhibitory activity of **12c** against MAPK11 was slightly reduced, which may be caused by steric hindrance between the hydroxyl group in the S configuration and the surrounding residues. In addition, the compound **12d**, introduced 1-propanol group, displayed better activity than **12c**, proving the above conjecture. It was also observed that the introduction of more polar groups, such as carboxylic acid and amide, improved the inhibitory activities of compounds **13a**, **13c**, and **14a** against MAPK11. Furthermore, **13a** and **14a** were found to be slightly more potent than **13c**, indicating that the linear three-carbon alkyl chain is more conducive than cyclobutene. However, when **13a** containing a carboxylic acid was replaced by the corresponding ester (**12f**), its potency decreased by about 10-fold. The above results suggest that introducing polar groups to the kinase solvent region is beneficial to maintain its bioactivity. The predicted binding mode of **13a** (IC_50_ = 6.4 nM, docking score = −10.564 kcal/mol) revealed the formation of a strong salt bridge interaction between the carboxyl group and the side chain of Arg28 in the solvent region. In addition, the 2,4-difluoro-substituted benzene ring was found to occupy the small hydrophobic pocket I around the gatekeeper residue Thr106, resulting in favorable hydrophobic interactions. The dibenzocycloheptanone scaffold of **13a** could induce the glycine to perform a 180° conformational flip in the hinge region, allowing the carbonyl oxygen of the scaffold to form hydrogen bond interactions with the backbone nitrogen atom of residues Met109 and Gly110 simultaneously (Figure 4A).

The binding mode of skepinone-L with MAPK11 showed that the hydrophobic II pocket of the kinase was not occupied completely. To increase its potency further, a small hydrophobic group, such as methyl, was introduced to the 9 position of the dibenzocycloheptanone core, which led to the design and synthesis of compounds **12b**, **12e**, and **13b**. The results showed that the introduction of methyl groups could indeed increase the activity of compounds **12a**, **12d**, and **13a** (Table 1). Moreover, the binding mode of **12b** revealed that the methyl group could occupy the cavity of the hydrophobic II pocket in MAPK11 (Figure 4B), which was consistent with our design strategy. Thereby, compound **13b**, with the best bioactivity (IC_50_ = 4.2 nM) as well as the docking score (−10.969 kcal/mol, Supplementary Materials Table S1), was obtained for further optimization, which was about 4.5-fold more potent than the lead compound **12a** (IC_50_ = 19.2 nM).

Attempting to obtain novel scaffold, we broke the seven-membered ring in the dibenzocycloheptanone moiety and replaced the carbonyl group with its bioisosteres sulfone and sulfoxide groups. Thus, several diarylsulfone and sulfoxide derivatives **23b**–**c**, **25**, and **26** were designed and synthesized (Table 2) through scaffold hopping. Unfortunately, all the diarylsulfone compounds **23b**–**c** and **26** lost bioactivities against MAPK11, with IC_50_ values larger than 10 μM. We speculated that the orientation of the two oxygens in the sulfone might have a significant impact on the glycine flip, causing it to lose the critical interactions in the hinge region. Moreover, the slight increase observed in the activity of the sulfoxide compound **25** supported the above hypothesis. However, when compared to dibenzocycloheptanone compounds, **25** displayed more than a hundred-fold decrease in potency (IC_50_ = 2547.3 nM), which may be attributed to the cycloheptanone ring-opening after replacing it with sulfoxide, thus resulting in the inability to induce glycine reversal and maintain the linear binding mode. The above results provide some reference to explore novel skeletons further.

## 3. Materials and Methods

### 3.1. Chemistry

The reagents used in the experiments were all commercially available, most of which were purchased from Energy Chemical Company, Shanghai, China. All the reactions were monitored by TLC. ^1^H NMR and ^13^C NMR spectra were recorded on a Bruker Avance-400 or Ascend 600 spectrometer in CDCl_3_ or DMSO-*d*_6_ with TMS as an internal standard. High-resolution mass spectra (HRMS) were acquired using a Xevo G2 TOF MS spectrometer in positive EI mode. Mass spectra for all intermediates were recorded on an Agilent Technologies 6100 Series Single Quadrupole LC/MS or Micromass GCT CA 055 instrument. Melting points (M.p.) were measured on a WRS-1B digital melting point apparatus and kept uncorrected. The purification of all the tested compounds was performed on an Agilent 1100 series HPLC using an Agilent XDB 5 μ C18 column (4.6 mm × 150 mm). Elution was carried out using water as mobile phase A and acetonitrile as mobile phase B, with conditions phase A 20% + phase B 80% during 25 min. The flow rate of the mobile phase was 0.5 mL/min, and the sample injection volume was 10 μL. The determined wavelength was 254 nm. The purity of all compounds was ≥95%, as determined by HPLC analysis.

The spectral data of the key compound is described below. In addition, detailed synthetic procedures and spectral data for other final compounds are provided in the Supplementary Materials (Figure S1).

4-((8-((2,4-difluorophenyl)amino)-1-methyl-5-oxo-10,11-dihydro-5H-dibenzo [a,d][7]annulen-3-yl)oxy)butanoic acid (13b)

Light yellow solid. Yield: 26.9%. M.p.: 120.4–120.6 ℃. ^1^H NMR (400 MHz, DMSO-*d*_6_) δ 8.55 (s, 1H), 7.76 (d, *J* = 8.7 Hz, 1H), 7.43–7.31 (m, 2H), 7.11–7.06 (m, 1H), 7.05 (d, *J* = 2.5 Hz, 1H), 6.94 (d, *J* = 2.2 Hz, 1H), 6.72 (d, *J* = 8.7 Hz, 1H), 6.62 (s, 1H), 3.95 (t, *J* = 6.7 Hz, 2H), 3.02–2.91 (m, 4H), 2.30 (s, 3H), 2.05 (t, *J* = 6.8 Hz, 2H), 1.87–1.81 (m, 2H).^13^C NMR (151 MHz, DMSO-*d*_6_) δ 193.7, 158.0 (dd, *J* = 245.6, 11.3 Hz), 155.2 (dd, *J* = 247.6, 11.8 Hz), 149.2, 145.1, 141.7, 137.0, 132.7, 132.0, 130.1, 128.7, 126.3 (dd, *J* = 10.3, 2.1 Hz), 126.2, 125.6 (dd, *J* = 8.7, 8.5 Hz), 120.7, 114.0, 112.6, 112.3, 112.3 (dd, *J* = 22.3, 3.8 Hz), 105.4 (dd, *J* = 25.4, 25.2 Hz), 68.2, 35.1, 28.6, 27.0, 26.4, 20.5. HRMS (EI): calcd for C_26_H_23_F_2_NO_4,_ [M+H]^+^ m/z 451.1595; found 451.1592. Purity: 96.5%, retention time = 10.78 min.

### 3.2. Enzyme Assay against MAPK11

The effect of the above synthesized inhibitors on MAPK11 kinase activity in vitro was assessed using mobility shift assay by ChemPartner. Following the kinase reaction, a part of the substrate was added to a phosphate group under the action of the kinase to yield a product. The substrate and the product have a difference of 3 charges, thereby allowing them to be separated and detected by capillary electrophoresis. In these assays, SB202190 was used as a positive control, and at least two parallel experiments were carried out. Finally, the data was fitted to the XLFit Excel plug-in version 5.4.0.8 to calculate the IC_50_ value.

### 3.3. Molecular Docking

The X-ray structure of MAPK11 (PDB code: 3GC8) was downloaded from the Protein Data Bank. The MAPK11 structure was prepared using the Protein Preparation Wizard in Maestro (Schrodinger, Inc., New York, NY, USA, version 10.2). Energy minimization was performed with a root mean square deviation (RMSD) value of 0.3 Å using OPLS-2005 force field. The optimized structures of compounds were generated using the LigPrep module with pH of 7.0 ± 2.0 for Epik. The prepared compounds were docked into the ATP pocket using Glide with default settings.

## 4. Conclusions

In conclusion, a series of dibenzocycloheptanone derivatives were designed, synthesized, and evaluated as novel and potent MAPK11 inhibitors. Among them, **13a** and **13b**, with IC_50_ values of 6.40 nM and 4.20 nM, respectively, were found to be the most potent, namely 3- and 4.5-fold more potent than **12a** (skepinone-L, IC_50_ = 19.2 nM). Furthermore, to elucidate the structure–activity relationship, the binding modes of these compounds to MAPK11 were discussed, which would be beneficial for guiding future optimization of more potent MAPK11 inhibitors. Considering the significant inhibitory activity of dibenzocycloheptanone compounds against MAPK11, various anti-HD functional experiments will be further conducted for preliminarily evaluation of their ability to degrade mHTT protein in vitro.

## Figures and Tables

**Table 1:** Inhibitory activities of dibenzocycloheptanone derivatives against the MAPK11 enzyme ^a^.

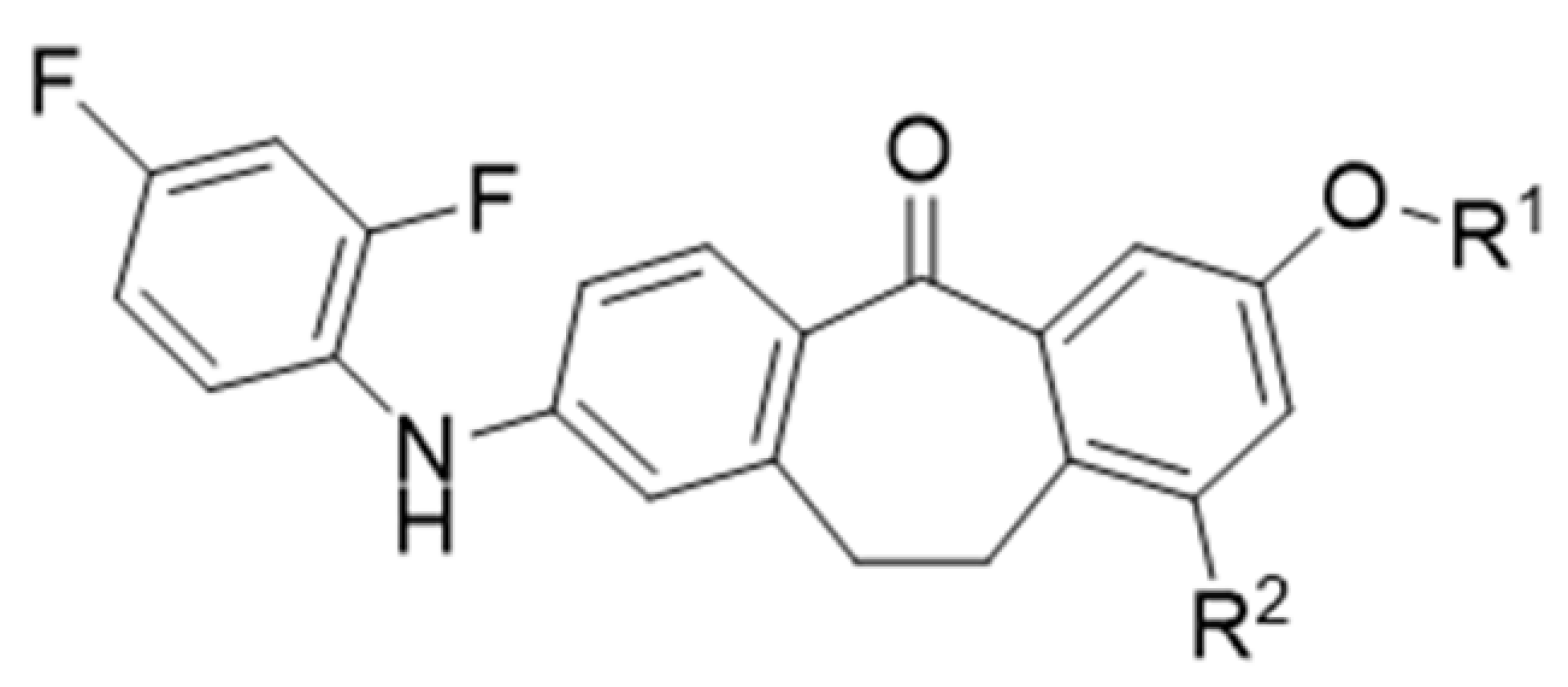			
**Compounds**	**R^1^**	**R^2^**	**MAPK11 Enzyme Inhibitory Activity IC_50_/nM**
**12a (Skepinone-L)**	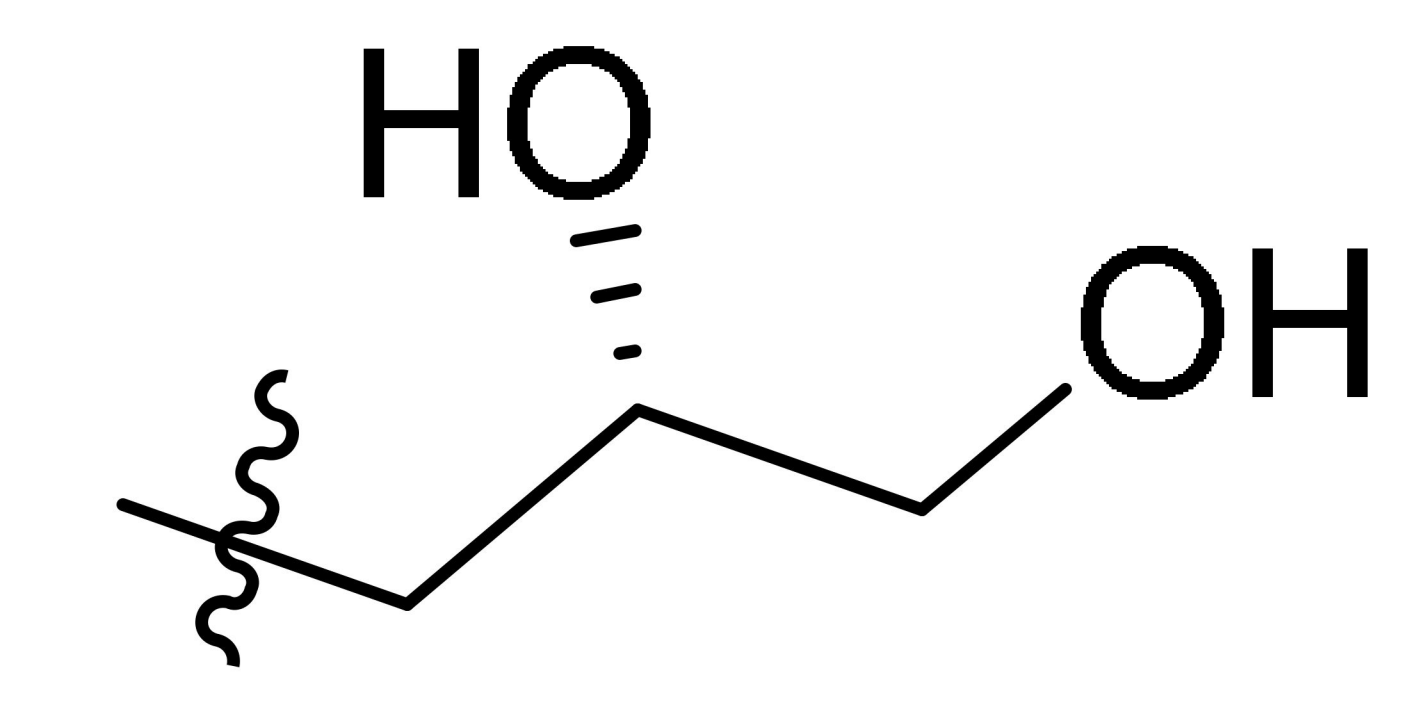	H	19.2 ± 0.9
**12c**	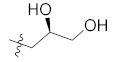	H	32.0 ± 0.6
**12d**	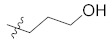	H	25.9 ± 0.7
**12f**	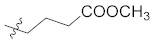	H	61.0 ± 2.1
**13a**	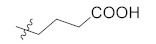	H	6.4 ± 0.6
**13c**	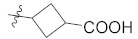	H	14.3 ± 0.6
**14a**	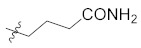	H	8.4 ± 0.1
**12b**	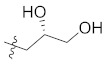	CH_3_	14.5 ± 0.3
**12e**	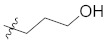	CH_3_	16.4 ± 0.6
**13b**	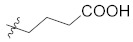	CH_3_	4.2 ± 0.1
SB202190 ^b^			34.2 ± 0.8

^a^ Kinase activities were determined by using mobility shift assay. IC_50_ values are averages of at least two independent determinations and reported as the mean ± SD. ^b^ SB202190 was used as the positive control.

**Table 2:** Inhibitory activities of diarylsulfone and sulfoxide derivatives against MAPK11 enzyme ^a^.

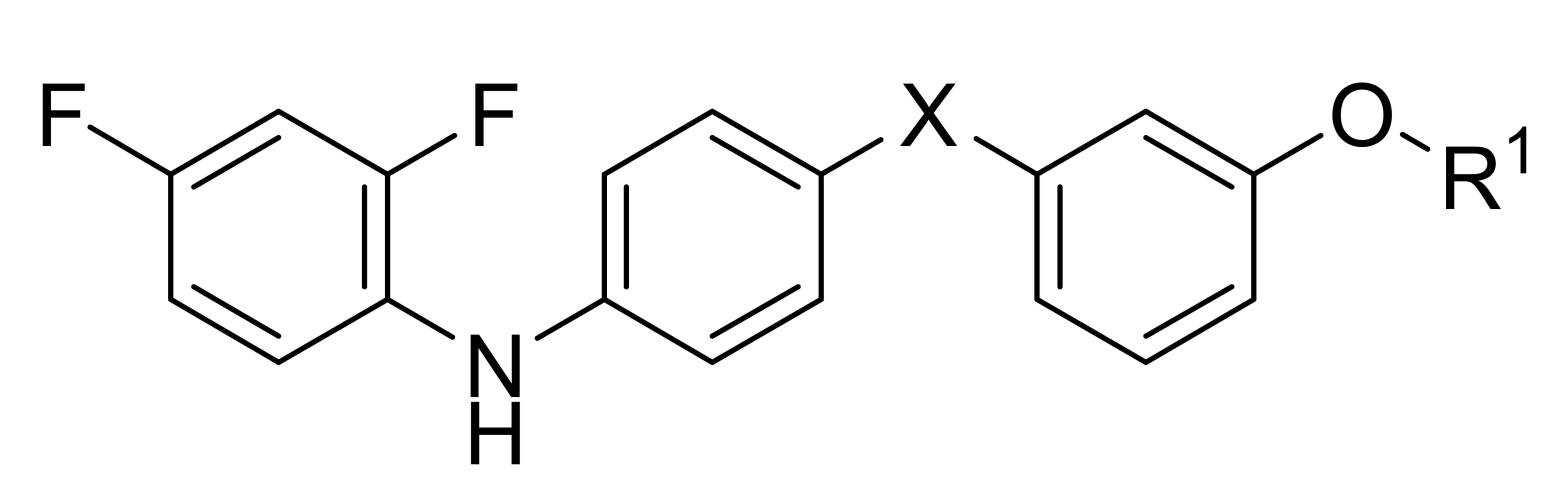			
**Compounds**	**X**	**R^1^**	**MAPK11 Enzyme Inhibitory Activity** **IC_50_/nM**
**23b**	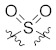	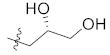	>10,000
**23c**	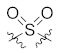	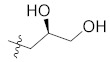	>10,000
**26**	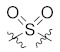	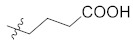	>10,000
**25**	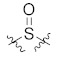	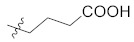	2547.3 ± 682.1
**12a** (**Skepinone-L**)			19.2 ± 0.9
SB202190 ^b^			34.2 ± 0.8

^a^ Kinase activities were determined by using mobility shift assay. IC_50_ values are averages of at least two independent determinations and reported as the mean ± SD. ^b^ SB202190 was used as the positive control.

## Supplementary Materials

The following supporting information can be downloaded online, Table S1: Glide docking scores of compounds, Figure S1: The spectra of 1H NMR, 13C NMR and HRMS (EI) of representative compounds.

## Sample Availability

Samples of the compounds **12a–f**, **13a–c**, **23b–c**, **25** and **26** are available from the authors.
