# Supplementary material for: Design, Synthesis, and Structure–Activity Relationship Study of Potent MAPK11 Inhibitors
Source: Molecules. 2021 Dec 29;27(1):203. doi: 10.3390/molecules27010203 (PMC8746797; doi:10.3390/molecules27010203)
Supplement: Supplementary file 1 [file molecules-27-00203-s001.zip › molecules-1507463-supplementary.pdf]

Supplementary information

Design, Synthesis and Structure-activity Relationship Study of Potent MAPK11 Inhibitors

Mengdie Gong [1,†], Mingyan Tu [1,†], Hongxia Sun [1,†], Lu Li [1], Lili Zhu [1], Honglin Li [1,2], Zhenjiang Zhao [1,*] and Shiliang Li [1,*]

[1]Shanghai Key Laboratory of New Drug Design, School of Pharmacy, East China University of Science & Technology, Shanghai 200237, China
[2] Jiangzhong Pharmaceutical Co., Ltd., Nanchang 330096, China
†Authors contributed equally to this work.
* Correspondence: author. zhjzhao@ecust.edu.cn, shiliangli@ecust.edu.cn

Content

Chemistry 2
General procedure for compounds **12-14**. 2
General procedure for compounds **23-26**. 10
References. 14
Table S1. Glide docking scores of compounds. 15
Figure S1. The spectra of ^{1}H NMR, ^{13}C NMR and HRMS (EI) of representative compounds 15

Chemistry

All chemical reagents and solvents used in experiments were of commercial grade. Silica gel (300-400 mesh) used for column chromatography and thin-layer chromatography (TLC) used to monitor the progress of the reaction were purchased from Qingdao Haiyang Chemical Co., Ltd. ^1H and ^{13}C NMR spectra were performed on the Bruker spectrometer (AV-400) at 400 and 100 MHz, respectively. The low-resolution mass spectra (LC-MS) were measured using Agilent 6120 LC-MS. The high-resolution mass spectra (HRMS, Waters LCT Premier XE TOF) were measured at the Institute of Fine Chemistry of ECUST. Melting points were determined with the digital melting point apparatus (WRS-1B). The purity of the final compounds was analyzed by HPLC (Hewlett-Packard 1100). The HPLC instrument was equipped with a photodiode array detector using a Zorbax RP-18 column (5 μm, 4.6 mm × 250 mm, reverse phase column). The mobile phases A and B were acetonitrile and purified water, respectively.

General procedure for compounds 12–14.

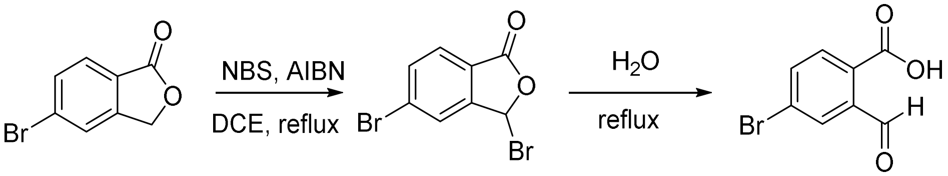

5-bromoisobenzofuran-1(3H)-one **1** (3.35 g, 15.79 mmol) was dissolved in 1,2-dichloroethane (70 mL). Then N-Bromosuccinimide (NBS, 3.10 g, 17.36 mmol) was added. Until the solution was heated to 80 °C, the solution of azobisisobutyronitrile (AIBN, 100 mg, 0.63 mmol) in 1,2-dichloroethane (2 mL) was added dropwise. After stirred for 12 h at 80 °C, the reaction solution was distilled off under reduced pressure. Then a solid was precipitated after a small amount of dichloromethane was added dropwise. The suspension was filtered and the filtrate was collected to distill off under reduced pressure to give the 3,5-dibromoisobenzofuran-1(3H)-one **2** (2.90 g, 63.0%) as a brown viscous liquid which directly used in the synthesis of **3**.

3,5-dibromoisobenzofuran-1(3H)-one **2** (2.90 g, 9.93 mmol) was dissolved in an appropriate amount of water, then heated to 105 °C to reflux for 2 h until solid particles were produced. The mixture was extracted three times with ethyl acetate. Then combined organic layers were washed once with brine and dried over Na_2SO_4, filtered, and the solvent was removed by rotary evaporation to obtain the 4-bromo-2-formylbenzoic acid **3**[1] (2.29 g, 63.5%) without further purification. ^1H NMR (400 MHz, DMSO-d_6) δ 8.29 (s, 1H), 7.92 (s, 1H), 7.86 (d, J = 8.0 Hz, 1H), 7.77 (d, J = 8.0 Hz, 1H), 6.65 (s, 1H). LC-MS(ESI) calcd for $C_8H_5BrO_3$ $[M+H]^+$ 231.03, found 231.05.

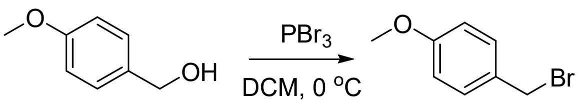

(4-methoxyphenyl) methanol **4a** (6.90 g, 50.00 mmol) was dissolved in anhydrous dichloromethane, stirred in ice bath, and the solution of phosphorus tribromide (PBr_3, 6.80 g, 25.00 mmol) in dichloroethane (10 mL) were added dropwise. After 1 h at room temperature, the saturated $NaHCO_3$ solution was added slowly, accompanied by bubbles. The mixture was extracted three times with dichloroethane. Then combined organic layers were washed once with brine and dried over Na_2SO_4, filtered, and the solvent was removed by rotary evaporation to obtain the crude 1-(bromomethyl)-4-methoxybenzene **5a**[2] (9.90 g, 98.5%). LC-MS(ESI) calcd for C_8H_9BrO $[M-H_2O+H]^+$ 184.06, found 184.85.

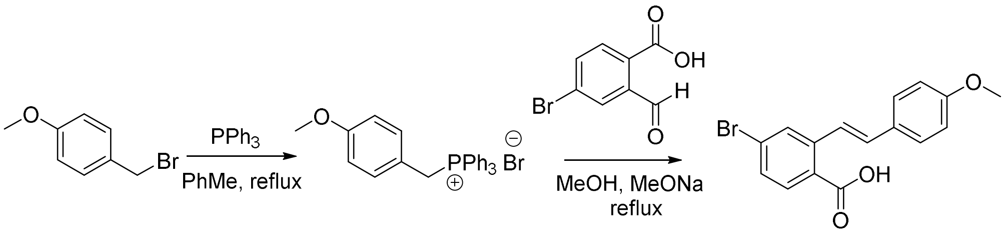

After triphenyl phosphine (PPh_3, 12.98 g, 49.50 mmol) dissolved in toluene (70 mL), the crude 1-(bromomethyl)-4-methoxybenzene **5a** (9.90 g, 49.50 mmol) was added. After reflux reaction for 12 h at 80 °C, a white solid was precipitated, and the filter cake was collected by suction filtration with a suction filter and dried in an oven to obtain phosphine ylide reagent (16.70 g, 73.0%).

The phosphine ylide reagent (2.18 g, 4.40 mmol) was dissolved in methanol (30 mL), stirred in ice bath, then the 4-bromo-2-formylbenzoic acid **3** (1.00 g, 4.40 mmol) and the sodium methoxide solution (710 mg, 13.2 mmol) dissolved in methanol (4 mL) were added. After stirred for 10 h at 70 °C, the ice water was poured and to adjust the pH to acidity using diluted hydrochloric acid. The mixture was extracted three times with dichloroethane. Then combined organic layers were washed once with brine and dried over Na_2SO_4, filtered, and the solvent was removed by rotary evaporation to obtain the crude (E)-4-bromo-2-(4-methoxystyryl) benzoic acid **6a**[3] (1.37 g, 85.0%) as a light yellow solid. LC-MS(ESI) calcd for $C_{16}H_{13}BrO_3$ $[M+H]^+$ 334.18, found 334.20.

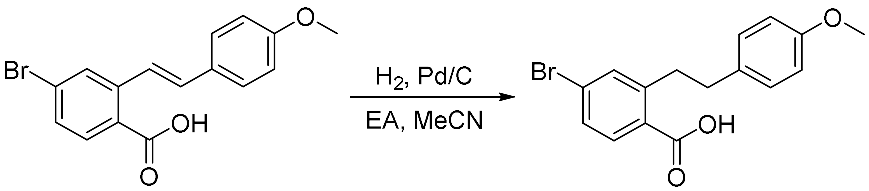

(E)-4-bromo-2-(4-methoxystyryl) benzoic acid **6a** (200 mg, 0.60 mmol) was dissolved in ethyl acetate (2 mL) and acetonitrile (2 mL), and charged Pd/C (20 mg, 10%) into reaction solution stirred at room temperature for 12 h. The suspension was filtered and filtrate was distilled off under reduced pressure. The residue was purified by silica gel chromatography (petroleum ether/ethyl acetate = 2:1, v/v) to give the product 4-bromo-2-(4-methoxyphenethyl) benzoic acid **7a**[3] (130 mg, 65.0%) as a white solid.^{1}H NMR (400 MHz, $CDCl_3$) δ 7.97 (dd, *J* = 7.8, 1.0 Hz, 1H), 7.56 (d, *J* = 2.8 Hz, 1H), 7.42 (td, *J* = 7.4, 1.2 Hz, 1H), 7.31 (t, *J* = 7.2 Hz, 1H), 7.21 (d, *J* = 7.6 Hz, 1H), 7.12 (d, *J* = 8.4 Hz, 1H), 6.99 (dd, *J* = 8.4, 2.8 Hz, 1H), 3.84 (s, 3H), 3.15 (d, *J* = 2.1 Hz, 4H). LC-MS(ESI) calcd for $C_{16}H_{15}BrO_3$ $[M+H]^+$ 336.20, found 336.05.

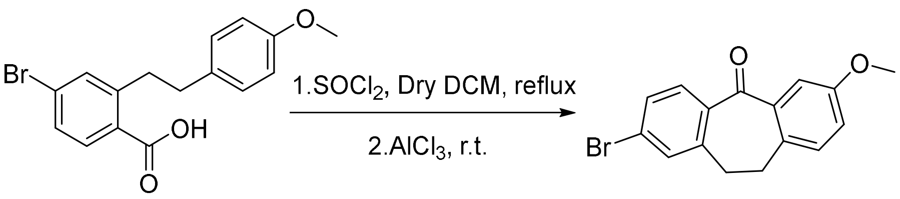

4-bromo-2-(4-methoxyphenethyl) benzoic acid **7a** (1.50 g, 4.50 mmol) was dissolved in anhydrous dichloromethane (10 mL), then thionyl chloride (1 mL, 13.50 mmol) was added dropwise. After reflux reaction for 1 h at 40 °C, and cooled to room temperature, aluminum chloride (780 mg, 5.85 mmol) was added to stir for 3 h. After quenching with cold water, the reaction solution was extracted three times with ethyl acetate. Then combined organic layers were washed once with brine and dried over Na_2SO_4, filtered, and the solvent was removed by rotary evaporation. The residue was purified by silica gel chromatography (petroleum ether/ethyl acetate = 2:1, v/v) to give the product 2-bromo-7-

methoxy-10,11-dihydro-5H-dibenzo[a,d][7]annulen-5-one **8a**[3] (680 mg, 47.7%) as a yellow viscous liquid. ^{1}H NMR (400 MHz, $CDCl_3$) δ 7.87 (d, *J* = 8.4 Hz, 1H), 7.60 – 7.52 (m, 1H), 7.48 – 7.30 (m, 2H), 7.13 (d, *J* = 8.4 Hz, 1H), 7.06 – 6.96 (m, 1H), 3.85 (s, 3H), 3.33 – 3.00 (m, 4H). LC-MS(ESI) calcd for $C_{16}H_{13}BrO_2$ $[M+H]^+$ 318.18, found 318.00.

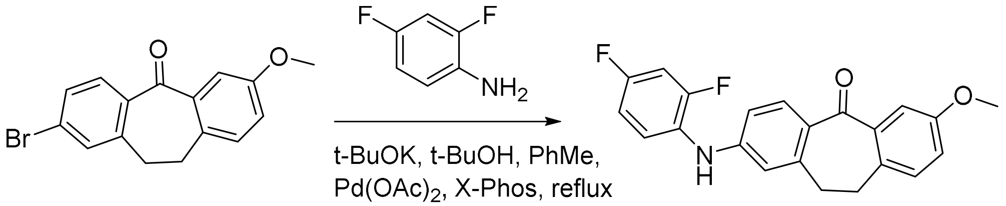

2-bromo-7-methoxy-10,11-dihydro-5H-dibenzo[a,d][7]annulen-5-one **8a** (50 mg, 0.16 mmol) was dissolved in toluene (5 mL), then 2,4-difluoroaniline **9** (22.50 mg, 0.17 mmol), potassium tert-butoxide (165 mg, 1.47 mmol), palladium acetate (5 mg, 0.02 mmol), 2-dicyclohexylphosphorus-2,4,6-triisopropylbiphenyl (15 mg, 0.03 mmol), tert-butanol (2 mL) were added in turn. After reflux reaction for 24 h at 110 °C in the protection of nitrogen, the reaction solution was quenched with water, and extracted three times with dichloroethane. Then combined organic layers were washed once with brine and dried over Na_2SO_4, filtered, and the solvent was removed by rotary evaporation. The residue was purified by silica gel chromatography (petroleum ether/ethyl acetate = 5:1, v/v) to give the product 2-((2,4-difluorophenyl)amino)-7-methoxy-10,11-dihydro-5H-dibenzo[a,d][7] annulen-5-one **10a**[3] (49 mg, 83.9%) as a yellow viscous liquid. ^{1}H NMR (400 MHz, $CDCl_3$) δ 8.13 (d, *J* = 8.7 Hz, 1H), 7.59 (d, *J* = 2.8 Hz, 1H), 7.36 (d, *J* = 5.7 Hz, 1H), 7.26 (s, 1H), 7.12 (d, *J* = 8.3 Hz, 1H), 7.00-6.83 (m, 4H), 6.67 (d, *J* = 2.3 Hz, 1H), 3.85 (s, 3H), 3.09 (s, 4H). LC-MS(ESI) calcd for $C_{22}H_{17}F_2NO_2$ $[M+H]^-$ 366.38, found 366.20.

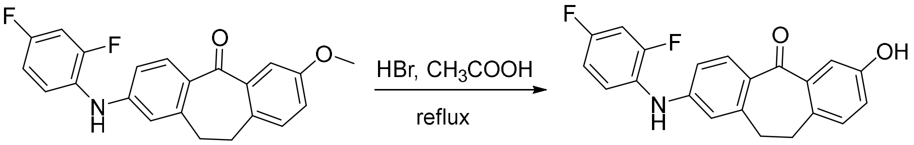

2-((2,4-difluorophenyl)amino)-7-methoxy-10,11-dihydro-5H-dibenzo[a,d][7]annulen-5-one **10a** (49 mg) was directly dissolved in acetic acid (1 mL) and hydrobromic acid (1 mL). After reflux reaction for 5 h at 100 °C, the reaction solution was quenched with little cold water and the solid was precipitated to collect the filter cake by suction filtration with a suction filter, and dried in an oven to obtain the product 2-((2,4-difluorophenyl)amino)-7-hydroxy-10,11-dihydro-5H-dibenzo[a,d][7]annulen-5-one **11a**[3] (34.50 mg, 61.4%) as a brown solid. ^{1}H NMR (400 MHz, DMSO-d_6) δ 9.51 (s, 1H), 8.55 (s, 1H), 7.94 (d, *J* = 8.8 Hz, 1H), 7.39 (m, 2H), 7.30 (d, *J* = 2.7 Hz, 1H), 7.09 (d, *J* = 3.3 Hz, 1H), 6.86 (dd, *J* = 8.2, 2.7 Hz, 1H), 6.73 (d, *J* = 8.6 Hz, 1H), 6.60 (s, 1H), 3.08-2.85 (m, 4H). LC-MS(ESI) calcd for $C_{21}H_{15}F_2NO_2$ $[M+H]^+$ 352.11, found 352.20.

(R)-2-((2,4-difluorophenyl)amino)-7-(2,3-dihydroxypropoxy)-10,11-dihydro-5H-dibenzo [a,d][7]annulen-5-one (12a).

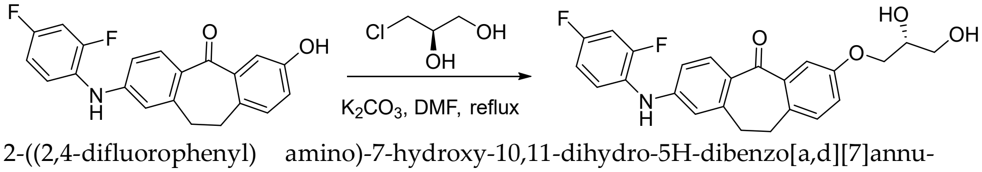

2-((2,4-difluorophenyl) amino)-7-hydroxy-10,11-dihydro-5H-dibenzo[a,d][7]annulen-5-one **11a** (34.50 mg, 0.10 mmol) was dissolved in anhydrous *N, N*-dimethylformamide (10 mL). Then (R)-(+)-3-chloro-1,2-propanediol (14 mg, 0.13 mmol) and potassium carbonate were added. After reflux reaction for 24 h at 80 °C in the protection of nitrogen, the reaction solution was extracted three times with dichloroethane. Then combined organic layers were washed once with brine and dried over Na_2SO_4, filtered, and the solvent

was removed by rotary evaporation. The residue was purified by silica gel chromatography (petroleum ether/ethyl acetate = 2:1, v/v) to give the product (R)-2-((2,4-difluorophenyl)amino)-7-(2,3-dihydroxypropoxy)-10,11-dihydro-5H-dibenzo [a,d] [7]annulen -5-one **12a** (11 mg, 26.4%) as a light yellow solid. Mp: 131.2-132.1 °C. ^{1}H NMR (400 MHz, DMSO-d_6) δ 8.62 (s, 1H), 8.01 (d, *J* = 8.8 Hz, 1H), 7.52 – 7.36 (m, 3H), 7.28 (d, *J* = 8.4 Hz, 1H), 7.20 – 7.06 (m, 2H), 6.85 – 6.75 (m, 1H), 6.67 (s, 1H), 5.03 (d, *J* = 5.1 Hz, 1H), 4.75 (t, *J* = 5.7 Hz, 1H), 4.08 (dd, *J* = 9.7, 4.0 Hz, 1H), 3.98 – 3.90 (m, 1H), 3.88 – 3.82 (m, 1H), 3.51 (t, *J* = 5.6 Hz, 2H), 3.06 (s, 4H). ^{13}C NMR (151 MHz, DMSO-d_6) δ 190.7, 158.7 (dd, *J* = 243.2, 11.2 Hz), 157.6, 155.9 (dd, *J* = 248.1, 12.3 Hz), 149.4, 145.7, 139.9, 134.5, 133.8, 130.6, 127.7, 126.7 (dd, *J* = 12.2, 3.8 Hz), 125.6 (dd, *J* =9.7, 9.5 Hz), 119.1, 115.4, 113.8, 112.5, 112.4 (dd, *J* = 23.2, 4.8 Hz), 105.7 (dd, *J* = 28.4, 28.2 Hz), 63.0, 36.2, 33.5, 31.5, 30.1. HRMS (EI): calcd for $C_{24}H_{21}F_2NO_4$, [M]$^+$: m/z: 425.1439; found 425.1438. HPLC purity: 98.2%, retention time = 10.12 min. $[\alpha]_D^{25}$ = -105.7 (*c* 1.00, CH_2Cl_2).

The following compounds **12c-d**, **12f**, **13a**, **13c** and **14a** were prepared by a method similar to that for the synthesis of compound **12a** from the intermediates **4a-11a**.

(S)-2-((2,4-difluorophenyl)amino)-7-(2,3-dihydroxypropoxy)-10,11-dihydro-5H-dibenzo[a,d] [7]annulen-5-one (12c).

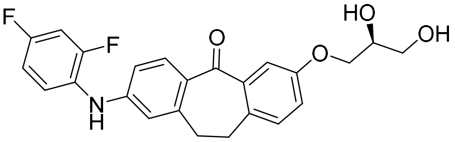

Light yellow solid. Yield: 28.9%. Mp: 134.5-135.3 °C. ^{1}H NMR (400 MHz, DMSO-d_6) δ 8.57 (s, 1H), 7.96 (d, *J* = 8.8 Hz, 1H), 7.43-7.38 (m, 2H), 7.37 (s, 1H), 7.23 (d, *J* = 8.4 Hz, 1H), 7.15-7.09 (m, 1H), 7.08-7.03 (m, 1H), 6.72 (d, *J* = 8.4 Hz, 1H), 6.62 (s, 1H), 4.96 (d, *J* = 5.2 Hz, 1H), 4.68 (s, 1H), 4.03 (dd, *J* = 8.8, 2.8 Hz, 1H), 3.83 (dd, *J* = 9.6, 6.4 Hz, 1H), 3.80 (t, *J* = 4.8 Hz, 1H), 3.45 (t, *J* = 5.4 Hz, 2H), 3.01 (s, 4H). ^{13}C NMR (151 MHz, DMSO-d_6) δ 190.5, 158.6 (dd, *J* = 243.2, 11.2 Hz), 157.3, 155.9 (dd, *J* = 248.1, 12.3 Hz), 149.2, 145.5, 139.7, 134.3, 133.6, 130.3, 128.9, 127.7 (dd, *J* = 12.2, 3.8 Hz), 126.4 (dd, *J* =9.7, 9.5 Hz), 119.7, 115.1, 113.6, 112.7, 112.4 (dd, *J* = 23.2, 4.8 Hz), 104.9 (dd, *J* = 28.4, 28.2 Hz), 70.0, 37.2, 33.7, 31.2, 29.8. HRMS (EI): calcd for $C_{24}H_{21}F_2NO_4$ [M+H]$^+$ m/z 425.1439, found 425.1440. HPLC purity: 97.3%, retention time = 10.87 min. $[\alpha]_D^{25}$ = +105.7 (*c* 1.00, CH_2Cl_2).

2-((2,4-difluorophenyl)amino)-7-(3-hydroxypropoxy)-10,11-dihydro-5H-dibenzo[a,d][7] annulen-5-one (12d).

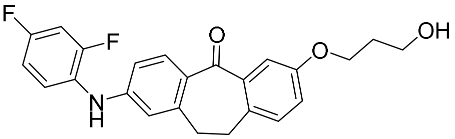

Light yellow solid. Yield: 32.7%. Mp: 123.5-123.9 °C. ^{1}H NMR (400 MHz, DMSO-d_6) δ 7.7 (s, 1H), 7.1 (d, *J* = 8.8 Hz, 1H), 6.6 (tdd, *J* = 11.4, 9.1, 2.8 Hz, 3H), 6.4 (d, *J* = 8.4 Hz, 1H), 6.3 – 6.2 (m, 2H), 5.9 (d, *J* = 8.8 Hz, 1H), 5.8 (s, 1H), 3.7 (t, *J* = 5.2 Hz, 1H), 3.2 (q, *J* = 7.3, 6.8 Hz, 2H), 2.7 (q, *J* = 5.6 Hz, 2H), 2.2 (s, 4H), 1.68 – 1.66 (m, 2H). ^{13}C NMR (151 MHz, DMSO-d_6) δ 190.7, 158.7 (dd, *J* = 243.4, 11.6 Hz), 157.6, 157.2 (dd, *J* = 248.3, 12.7 Hz), 149.4, 145.7, 139.9, 134.5, 133.8, 130.5, 127.7, 126.4 (dd, *J* = 9.8, 3.0 Hz), 125.2 (dd, *J* = 11.9, 3.5 Hz), 119.0, 115.2, 113.8, 112.5, 112.2 (dd, *J* = 22.0, 4.0 Hz), 106.1 (dd, *J* = 26.9, 26.7 Hz), 65.1, 57.6, 36.2, 33.5, 32.4. HRMS (ESI): calcd for $C_{24}H_{21}F_2NO_3$ [M+H]$^+$ m/z 409.1489, found 409.1491. HPLC purity: 96.8%, retention time =10.33 min.

4-((8-((2,4-difluorophenyl)amino)-5-oxo-10,11-dihydro-5H-dibenzo[a,d][7]annulen-3-yl)oxy) methylbutanoate (12f).

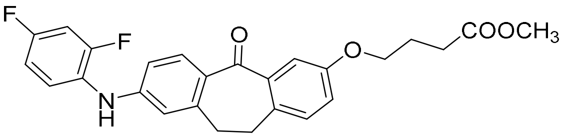

Light yellow solid. Yield: 47.6%. Mp: 124.6-125.0 °C.^{1}H NMR (400 MHz, $CDCl_3$) δ 8.05 (d, *J* = 8.7 Hz, 1H), 7.48 (d, *J* = 2.8 Hz, 1H), 7.28 (td, *J* = 9.0, 5.7 Hz, 1H), 7.03 (d, *J* = 8.3 Hz, 1H), 6.93 – 6.73 (m, 4H), 6.59 (d, *J* = 2.3 Hz, 1H), 5.81 (s, 1H), 3.97 (t, *J* = 6.1 Hz, 2H), 3.62 (s, 3H), 3.01 (s, 4H), 2.45 (t, *J* = 7.3 Hz, 2H), 2.04 (p, *J* = 6.6 Hz, 2H). ^{13}C NMR (151 MHz, $CDCl_3$) δ 192.1, 173.7, 158.5 (dd, *J* = 245.6, 11.3 Hz), 157.5, 155.1 (dd, *J* = 247.4, 11.8 Hz), 147.5, 145.5, 139.8, 134.5, 134.1, 130.1, 129.8, 125.1 (dd, *J* = 11.9, 3.6 Hz), 123.4 (dd, *J* = 9.3, 9.1 Hz), 119.6, 115.0, 114.7, 113.3, 111.4 (dd, *J* = 22.2, 3.8 Hz), 105.1 (dd, J = 26.7, 26.5 Hz), 66.9, 51.7, 36.3, 33.9, 30.6, 24.6. HRMS (EI): calcd for $C_{26}H_{23}F_2NO_4$ $[M+H]^+$ m/z 451.1595, found 451.1593. HPLC purity: 98.2%, retention time = 11.34 min.

4-((8-((2,4-difluorophenyl)amino)-5-oxo-10,11-dihydro-5H-dibenzo[a,d][7]annulen-3-yl)oxy) butanoic acid (13a).

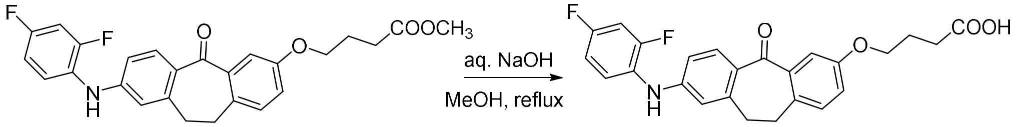

4-((8-((2,4-difluorophenyl)amino)-5-oxo-10,11-dihydro-5H-dibenzo[a,d][7]annulen-3-yl)oxy) methylbutanoate **12f** (40 mg, 0.09 mmol) was dissolved in methanol (2 mL) and 1N sodium hydroxide aqueous solution(40 mg, 1 mL). After reflux reaction for 1 h at 70 °C, the reaction solution was extracted three times with dichloromethane. Then combined organic layers were washed once with brine and dried over Na_2SO_4, filtered, and the solvent was removed by rotary evaporation The residue was purified by silica gel chromatography (petroleum ether/ethyl acetate = 2:1, v/v) to give the product 4-((8-((2,4-difluorophenyl)amino)-5-oxo-10,11-dihydro-5H-dibenzo[a,d][7]annulen-3-yl)oxy) butanoic acid **13a** (17 mg, 43.2%) as a light yellow solid. Mp: 143.4-144.2 °C. ^{1}H NMR (400 MHz, DMSO-d_6) δ 12.12 (s, 1H), 8.55 (s, 1H), 7.96 (d, *J* = 8.8 Hz, 1H), 7.51 – 7.32 (m, 3H), 7.24 – 7.20 (m, 1H), 7.15 – 7.01 (m, 2H), 6.75 (dd, *J* = 8.8, 1.6 Hz, 1H), 6.62 (s, 1H), 4.01 (t, *J* = 6.4 Hz, 2H), 3.01 (s, 4H), 2.39 (t, *J* = 7.2 Hz, 2H), 1.98-1.91 (m, 2H). ^{13}C NMR (151 MHz, $CDCl_3$) δ 190.5, 174.4, 158.5 (dd, *J* = 245.6, 11.3 Hz), 157.1, 155.1 (dd, *J* = 247.4, 11.8 Hz), 149.2, 145.5, 139.8, 134.4, 133.6, 130.4, 127.5, 126.2 (dd, *J* = 11.9, 3.6 Hz), 125.0 (dd, *J* = 9.3, 9.1 Hz), 118.8, 115.1, 114.7, 113.6, 112.3 (dd, *J* = 22.6, 4.2 Hz), 105.1 (dd, J = 26.5, 26.3 Hz), 66.9, 36.3, 33.3, 30.4, 24.5. HRMS (EI): calcd for $C_{25}H_{21}F_2NO_4$ $[M+H]^+$ m/z 437.1439, found 437.1441. HPLC purity: 98.7%, retention time = 10.65 min.

3-((8-((2,4-difluorophenyl)amino)-5-oxo-10,11-dihydro-5H-dibenzo[a,d][7]annulen-3-yl)oxy) cyclobutane-1-carboxylic acid (13c).

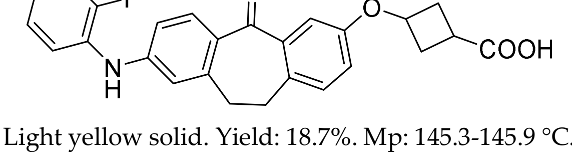

Light yellow solid. Yield: 18.7%. Mp: 145.3-145.9 °C. ^{1}H NMR (400 MHz, DMSO-d_6) δ 8.79 (s, 1H), 7.93 (d, *J* = 8.7 Hz, 1H), 7.44 – 7.33 (m, 2H), 7.29 (s, 1H), 7.19 (d, *J* = 7.9 Hz, 1H), 7.11 (d, *J* = 9.7 Hz, 1H), 6.94 (d, *J* = 6.6 Hz, 1H), 6.73 (d, *J* = 8.5 Hz, 1H), 6.60 (s, 1H), 4.51 (s, 1H), 2.98 (s, 4H), 2.12 (s, 1H), 1.75 (s, 4H), 1.22 (s, 1H).^{13}C NMR (151 MHz, DMSO-d_6) δ

190.9, 158.9 (dd, J = 243.3, 10.8 Hz), 156.1 (dd, J = 247.7, 12.4 Hz), 156.1, 149.7, 145.8, 140.1, 134.6, 133.9, 130.8, 127.7, 126.7 (dd, J = 12.5, 4.2 Hz), 125.4 (dd, J = 9.5, 9.3 Hz), 119.5, 115.9, 114.0, 112.6, 112.4 (dd, J = 22.3, 3.8 Hz), 105.6, 105.4 (dd, J = 25.7, 25.5 Hz), 67.8, 36.3(2C), 35.1, 34.9, 33.7. HRMS(EI): calcd for $C_{26}H_{21}F_2NO_4$ $[M+H]^+$ m/z 449.1439, found 449.1437. HPLC purity: 98.9%, retention time = 12.12 min.

4-((8-((2,4-difluorophenyl)amino)-5-oxo-10,11-dihydro-5H-dibenzo[a,d][7]annulen-3-yl)oxy) butanamide (14a).

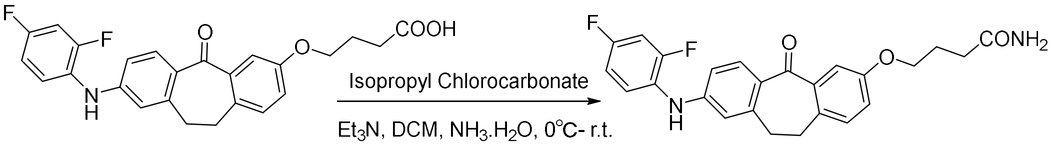

4-((8-((2,4-difluorophenyl)amino)-5-oxo-10,11-dihydro-5H-dibenzo[a,d][7]annulen-3-yl)oxy) butanoic acid **13a** (62 mg, 0.14 mmol) and triethylamine (42 mg, 0.42 mmol) were dissolved in dichloromethane (2 mL), ice bath, and then isopropyl chloroformate (25 mg, 0.21 mmol) was added dropwise. After reaction for 5 h at room temperature, 28% ammonia (2 mL) was added to continue the reaction for another 4 h. Then the reaction solution was extracted three times with ethyl acetate. All combined organic layers were washed once with brine and dried over Na_2SO_4, filtered, and the solvent was removed by rotary evaporation The residue was purified by silica gel chromatography (petroleum ether/ethyl acetate = 2:1, v/v) to give the product 4-((8-((2,4-difluorophenyl)amino)-5-oxo-10,11-dihydro-5H-dibenzo[a,d][7]annulen-3-yl)oxy) butanamide **14a** (15 mg, 24.2%) as a light yellow solid. Mp: 136.8-137.7 °C. ^1H NMR (400 MHz, DMSO-d_6) δ 8.56 (s, 1H), 7.96 (s, 1H), 7.38 (s, 4H), 7.21 (s, 1H), 7.07 (d, J = 21.7 Hz, 2H), 6.75 (s, 2H), 6.61 (s, 1H), 3.98 (s, 2H), 3.00 (s, 4H), 2.22 (s, 2H), 1.92 (s, 2H). ^{13}C NMR (151 MHz, DMSO-d_6) δ 190.9, 174.2, 158.9 (dd, J = 243.2, 11.6 Hz), 157.5, 156.1 (dd, J = 248.2, 12.5 Hz), 149.6, 145.8, 140.1, 134.7, 133.9, 130.7, 127.9, 126.6, 125.6 (dd, J = 9.4, 9.2 Hz), 119.2, 115.5, 114.0, 112.7, 112.3 (dd, J = 22.0, 3.9 Hz), 105.7 (dd, J = 26.6, 26.4 Hz), 67.6, 36.4, 33.7, 31.8, 25.2. HRMS (EI): calcd for $C_{25}H_{22}F_2N_2O_3$ $[M+H]^+$ m/z 436.1598, found 436.1603. HPLC purity: 98.7%, retention time = 10.45 min.

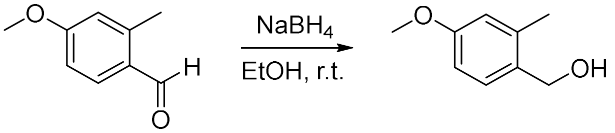

4-methoxy-2-methylbenzaldehyde was dissolved in absolute ethanol (30 mL) and sodium borohydride was added to stir at room temperature for 1 h. After adjusting pH to 4, the ethanol solvent was evaporated. Then the mixture was diluted with some water and extracted three times with ethyl acetate. All combined organic layers were washed once with brine and dried over Na_2SO_4, filtered, and the solvent was removed by rotary evaporation to give the crude (4-methoxy-2-methylphenyl)methanol **4b**[4] (3.00 g, 98.7%) as a viscous liquid. ^1H NMR (400 MHz, $CDCl_3$) δ 7.19 (d, J = 8.2 Hz, 1H), 6.89 – 6.47 (m, 2H), 4.56 (s, 2H), 3.76 (s, 3H), 2.32 (s, 3H). LC-MS(ESI) calcd for $C_9H_{12}O_2$ $[M-H_2O+H]^+$ 135.19, found 135.20.

The intermediates **5b-11b** were prepared by a method similar to that for the synthesis of the intermediates **5a-11a**. The following compounds **12b**, **12e** and **13b** were prepared from the intermediates **4b-11b** by a method similar to that for the synthesis of compound **12a**. The intermediates **3-11**[1-5] were prepared according to the corresponding literature procedure.

1-(bromomethyl)-4-methoxy-2-methylbenzene (5b[5]).

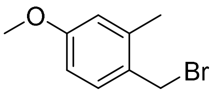

Light yellow viscous liquid. Yield: 24.3%. LC-MS(ESI) calcd for $C_9H_{11}BrO$ $[M+H]^+$ 216.19, found 216.20.

(E)-4-bromo-2-(4-methoxy-2-methylstyryl)benzoic acid (6b).

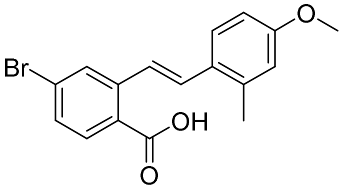

Light yellow solid. Yield: 65.7%. LC-MS(ESI) calcd for $C_{17}H_{15}BrO_3$ $[M+H]^+$ 348.21, found 348.05.

4-bromo-2-(4-methoxy-2-methylphenethyl)benzoic acid (7b).

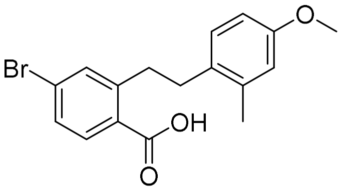

White solid. Yield: 37.7%. ^1H NMR (400 MHz, $CDCl_3$) δ 7.94 (d, *J* = 8.4 Hz, 1H), 7.46 (dd, *J* = 8.4, 2.0 Hz, 1H), 7.41 (d, *J* = 2.0 Hz, 1H), 7.05 (d, *J* = 8.0 Hz, 1H), 6.68 (m, 2H), 3.77 (s, 3H), 3.22 (dd, *J* = 10.8, 8.0 Hz, 2H), 2.86 (dd, *J* = 9.4, 5.6 Hz, 2H), 2.30 (s, 3H). LC-MS(ESI) calcd for $C_{17}H_{17}BrO_3$ $[M+H]^+$ 349.22, found 350.10.

8-bromo-3-methoxy-1-methyl-10,11-dihydro-5H-dibenzo[a,d][7]annulen-5-one (8b).

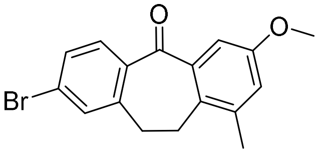

Yellow oily. Yield: 20.8%. ^1H NMR (400 MHz, $CDCl_3$) δ 7.68 (d, *J* = 8.4 Hz, 1H), 7.49-7.37 (m, 2H), 7.27 (d, *J* = 7.6 Hz, 1H), 6.92 (s, 1H), 3.82 (s, 3H), 3.29-2.97 (m, 4H), 2.31 (s, 3H). LC-MS(ESI) calcd for $C_{17}H_{15}BrO_2$ $[M+H]^+$ 332.21, found 333.05.

8-((2,4-difluorophenyl)amino)-3-methoxy-1-methyl-10,11-dihydro-5H-dibenzo[a,d][7] annulen-5-one (10b).

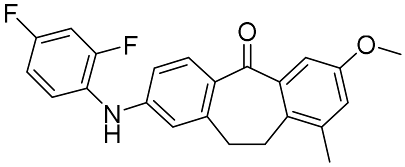

Yellow solid. Yield: 99.5%. ^1H NMR (400 MHz, $CDCl_3$) δ 7.97 (d, *J* = 8.6 Hz, 1H), 7.38 – 7.29 (m, 1H), 7.27 (d, 1H), 7.00 – 6.79 (m, 4H), 6.68 (d, *J* = 2.0 Hz, 1H), 5.83 (s, 1H), 3.81 (s, 3H), 3.06 (q, *J* = 9.2 Hz, 4H), 2.33 (s, 3H). LC-MS(ESI) calcd for $C_{23}H_{19}F_2NO_2$ $[M+H]^+$ 380.41, found 381.20.

8-((2,4-difluorophenyl)amino)-3-hydroxy-1-methyl-10,11-dihydro-5H-dibenzo[a,d][7] annulen-5-one (11b)

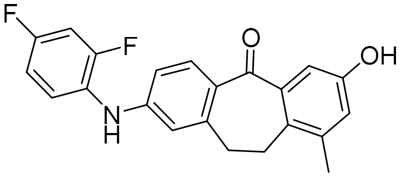

Brown solid. Yield: 84.2%. ^{1}H NMR (400 MHz, DMSO-d_6) δ 9.34 (s, 1H), 8.45 (s, 1H), 7.75 (d, *J* = 8.8 Hz, 1H), 7.44 – 7.31 (m, 2H), 7.09 (t, *J* = 8.4 Hz, 1H), 6.97 (d, *J* = 2.8 Hz, 1H), 6.76 (d, *J* = 2.4 Hz, 1H), 6.71 (d, *J* = 8.8 Hz, 1H), 6.61 (s, 1H), 2.94 (dd, *J* = 28.0, 9.2 Hz, 4H), 2.25 (s, 3H). LC-MS(ESI) calcd for $C_{22}H_{17}F_2NO_2$ $[M+H]^+$ 366.38, found 366.20.

(R)-8-((2,4-difluorophenyl)amino)-3-(2,3-dihydroxypropoxy)-1-methyl-10,11-dihydro-5H-dibenzo[a,d][7]annulen-5-one (12b).

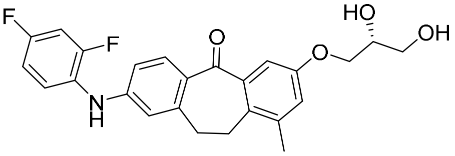

Light yellow solid. Yield: 24.3%. Mp: 135.1-135.8 °C. ^{1}H NMR (400 MHz, DMSO-d_6) δ 8.48 (s, 1H), 7.78 (d, *J* = 8.7 Hz, 1H), 7.49 – 7.28 (m, 2H), 7.08 (t, *J* = 5.9 Hz, 2H), 6.96 (d, *J* = 2.4 Hz, 1H), 6.72 (d, *J* = 8.7 Hz, 1H), 6.62 (s, 1H), 4.92 (d, *J* = 5.1 Hz, 1H), 4.65 (t, *J* = 5.7 Hz, 1H), 3.99 (dd, *J* = 9.7, 4.0 Hz, 1H), 3.84 (dd, *J* = 9.6, 6.3 Hz, 1H), 3.77 (dd, *J* = 10.2, 5.1 Hz, 1H), 3.43 (t, *J* = 5.5 Hz, 2H), 2.97 (dd, *J* = 18.0, 8.7 Hz, 4H), 2.31 (s, 3H). ^{13}C NMR (151 MHz, DMSO-d_6) δ 193.5, 158.6 (dd, *J* = 242.9, 11.6 Hz), 156.8, 155.8 (dd, *J* = 247.9, 12.6 Hz), 149.1, 145.0, 141.6, 136.9, 132.6, 132.1, 128.6, 126.1 (dd, *J* = 9.6, 3.0 Hz), 125.4 (dd, *J* = 12.1, 11.5 Hz), 120.5, 113.9, 112.5, 112.3, 112.1 (dd, *J* = 21.5, 3.5 Hz), 105.3 (dd, *J* = 26.4, 24.3 Hz), 70.2, 70.0, 63.0, 34.9, 28.5, 20.3. HRMS (EI): calcd for $C_{25}H_{23}F_2NO_4$, $[M+H]^+$ m/z 439.1595, found 439.1592. HPLC purity: 97.9%, retention time = 10.74 min. $[\alpha]_D^{25}$ = -111.3 (*c* 1.00, CH_2Cl_2).

8-((2,4-difluorophenyl)amino)-3-(3-hydroxypropoxy)-1-methyl-10,11-dihydro-5H-dibenzo [a,d][7]annulen-5-one (12e).

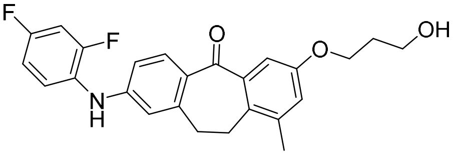

Light yellow solid. Yield: 34.5%. Mp: 127.2-127.7 °C. ^{1}H NMR (400 MHz, DMSO-d_6) δ 8.48 (s, 1H), 7.77 (d, J = 8.8 Hz, 1H), 7.46-7.28 (m, 2H), 7.09 (dd, J = 11.4, 2.0 Hz, 2H), 6.95 (d, J = 2.4 Hz, 1H), 6.70 (t, J = 12.0 Hz, 1H), 6.62 (s, 1H), 4.54 (t, J = 5.2 Hz, 1H), 4.03 (t, J = 6.0 Hz, 2H), 3.54 (dd, J = 11.6, 6.0 Hz, 2H), 2.96 (dd, J = 18.4, 8.8 Hz, 4H), 2.30 (s, 3H), 1.84 (p, J = 6.4 Hz, 2H). ^{13}C NMR (151 MHz, DMSO-d_6) δ 193.5, 158.6 (dd, *J* = 242.9, 11.6 Hz), 156.7, 155.8 (dd, *J* = 247.5, 12.7 Hz), 149.1, 145.0, 141.6, 136.9, 132.6, 132.0, 128.6, 126.4(dd, *J* = 12.7, 3.9 Hz), 120.5, 113.9, 112.5, 112.2, 112.1 (dd, *J* = 22.0, 3.5 Hz), 112.0, 105.2 (dd, *J* = 26.4, 24.3 Hz), 64.9, 57.6, 34.9, 32.4, 28.5, 20.3. HRMS (EI): calcd for $C_{25}H_{23}F_2NO_3$ $[M+H]^+$ m/z 423.1646, found 423.1648. HPLC purity: 96.7%, retention time = 10.23 min.

4-((8-((2,4-difluorophenyl)amino)-1-methyl-5-oxo-10,11-dihydro-5H dibenzo[a,d][7]annulen-3-yl)oxy)butanoic acid (13b).

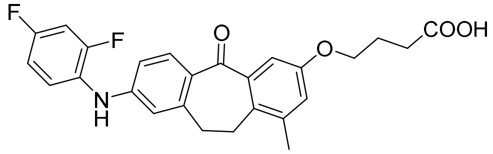

Light yellow solid. Yield: 26.9%. Mp: 120.4-120.6 °C. ^{1}H NMR (400 MHz, DMSO-*d*$_6$) δ 8.55 (s, 1H), 7.76 (d, *J* = 8.7 Hz, 1H), 7.43 – 7.31 (m, 2H), 7.11 – 7.06 (m, 1H), 7.05 (d, *J* = 2.5 Hz, 1H), 6.94 (d, *J* = 2.2 Hz, 1H), 6.72 (d, *J* = 8.7 Hz, 1H), 6.62 (s, 1H), 3.95 (t, *J* = 6.7 Hz, 2H), 3.02 – 2.91 (m, 4H), 2.30 (s, 3H), 2.05 (t, *J* = 6.8 Hz, 2H), 1.87 – 1.81 (m, 2H).^{13}C NMR (151 MHz, DMSO-*d*$_6$) δ 193.7, 158.0 (dd, *J* = 245.6, 11.3 Hz), 155.2 (dd, *J* = 247.6, 11.8 Hz), 149.2, 145.1, 141.7, 137.0, 132.7, 132.0, 130.1, 128.7, 126.3 (dd, *J* =10.3, 2.1 Hz), 126.2, 125.6 (dd, *J* =8.7, 8.5 Hz), 120.7, 114.0, 112.6, 112.3, 112.3 (dd, *J* = 22.3, 3.8 Hz), 105.4 (dd, *J* = 25.4, 25.2 Hz), 68.2, 35.1, 28.6, 27.0, 26.4, 20.5. HRMS (EI): calcd for $C_{26}H_{23}F_2NO_4$, $[M+H]^+$ m/z 451.1595; found 451.1592. Purity: 96.5%, retention time = 10.78 min.

General Procedure for Compounds 23–26.

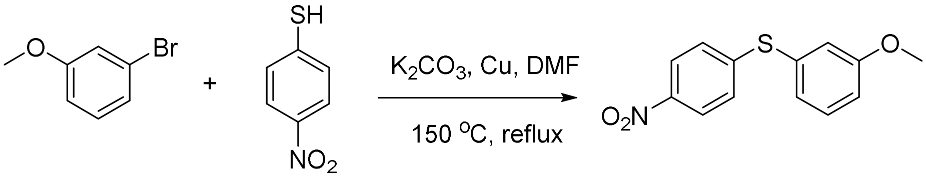

Bromo-3-methoxybenzene **15** (1.00 g, 5.00 mmol), 1-bromo-3-methoxybenzene **16**（920 mg, 6.00 mmol), potassium carbonate (1.50 g, 11.0 mol) and copper powder（1.00 g, 5.00 mmol）were in turn added in 50 mL two-necked reaction flask. Then anhydrous N, N-dimethylacetamide (20 mL) was added. After reflux reaction for 5 h at 150°C in the protection of nitrogen, the reaction solution was extracted three times with ethyl acetate. Then combined organic layers were washed once with brine and dried over Na_2SO_4, filtered, and the solvent was removed by rotary evaporation. The residue was purified by silica gel chromatography (petroleum ether/ethyl acetate = 20:1, v/v) to give the product (3-methoxyphenyl)(4-nitrophenyl)sulfane **17**[6] (500 mg, 35.7%) as a yellow solid. ^{1}H NMR (400 MHz, DMSO-*d*$_6$) δ 8.22 – 8.09 (m, 2H), 7.46 (t, J = 8.2 Hz, 1H), 7.36 – 7.28 (m, 2H), 7.18 – 7.07 (m, 3H), 3.79 (s, 3H). LC-MS(ESI) calcd for $C_{13}H_{11}NO_3S$ $[M+H]^+$ 262.30, found 262.10.

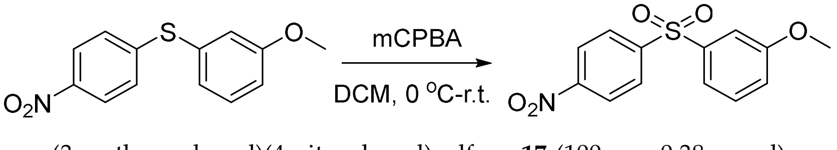

(3-methoxyphenyl)(4-nitrophenyl)sulfane **17** (100 mg, 0.38 mmol) was dissolved in dichloromethane (10 mL) and stirred in ice bath. Then m-chloroperoxybenzoic acid (mCPBA, 300 mg, 1.75 mmol) was added to react for 4 h at room temperature. The reaction solution was quenched with sodium sulfite aqueous solution, and extracted three times with dichloroethane. Then combined organic layers were washed once with brine and dried over Na_2SO_4, filtered, and the solvent was removed by rotary evaporation. The residue was purified by silica gel chromatography (petroleum ether/ethyl acetate = 5:1, v/v) to give the product 1-methoxy-3-((4-nitrophenyl)sulfonyl)benzene **18** (110 mg, 98.0%) as a yellow solid. ^{1}H NMR (400 MHz, DMSO-*d*$_6$) δ 8.44 – 8.35 (m, 2H), 8.30 – 8.22 (m, 2H), 7.58 (dd, *J* = 4.2, 1.4 Hz, 2H), 7.52 – 7.49 (m, 1H), 7.31 (m, 1H), 3.85 (s, 3H). LC-MS(ESI) calcd for $C_{13}H_{11}NO_5S[M+H_2O]^+$ 311.29, found 311.10.

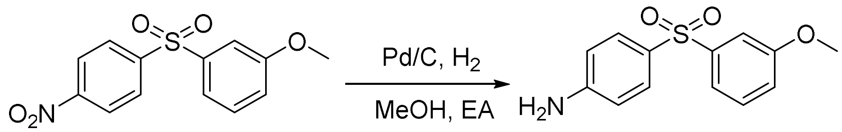

1-methoxy-3-((4-nitrophenyl)sulfonyl)benzene **18** (180 mg, 0.61 mmol) was dissolved in methanol (2 mL) and ethyl acetate (2 mL), and charged Pd/C (20.00 mg, 10%) into the reaction solution. Under hydrogen conditions, stirred for 1 h at room temperature, then the suspension was filtered and filtrate was distilled off under reduced pressure. The residue was purified by silica gel chromatography (petroleum ether/ethyl acetate = 2:1, v/v) to give the product 4-((3-methoxyphenyl)sulfonyl)aniline **19b**[7] (0.15 g, 92.6%) as a yellow solid. ^{1}H NMR (400 MHz, CD_3OD) δ 7.63 – 7.54 (m, 2H), 7.45 – 7.38 (m, 2H), 7.37 – 7.33 (m, 1H), 7.16 – 7.05 (m, 1H), 6.76 – 6.58 (m, 2H), 3.82 (s, 3H). LC-MS(ESI) calcd for $C_{13}H_{13}NO_3S[M+H]^+$ 264.31, found 264.10.

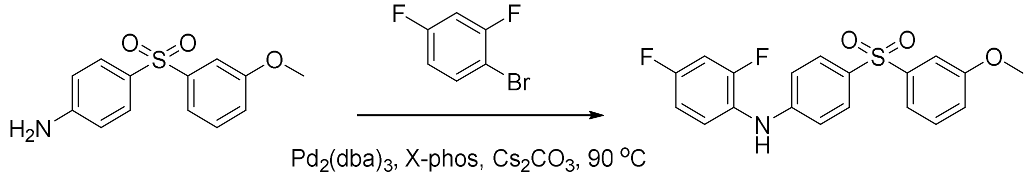

4-((3-methoxyphenyl)sulfonyl)aniline **19b** (150 mg, 0.57 mmol), 2,4-difluorobromobenzene **20** (96 mg, 0.50 mmol), $Pd_2(dba)_3$ (104 mg, 0.114 mmol), X-phos (68 mg, 0.14 mmol), cesium carbonate (Cs_2CO_3, 560 mg, 1.71 mmol) were added in turn. Then anhydrous *N*, *N*-dimethylacetamide (30 mL) was added. After reflux reaction for 10 h at 90 °C in the protection of nitrogen, the reaction solution was extracted three times with ethyl acetate. Then combined organic layers were washed once with brine and dried over Na_2SO_4, filtered, and the solvent was removed by rotary evaporation. The residue was purified by silica gel chromatography (petroleum ether/ethyl acetate = 5:1, v/v) to give the product 2,4-difluoro-N-(4-((3-methoxyphenyl)sulfonyl)phenyl)aniline **21b**[8] (130 mg, 60.7%) as a yellow viscous liquid. ^{1}H NMR (400 MHz, $CDCl_3$) δ 8.03 (s, 1H), 7.80 – 7.72 (m, 2H), 7.52 – 7.27 (m, 4H), 7.08 – 7.01 (m, 1H), 6.92-6.85 (m, 3H), 6.06 (s, 1H), 3.83 (s, 3H). LC-MS(ESI) calcd for $C_{19}H_{15}F_2NO_3S[M+H]^+$ 376.39, found 376.10.

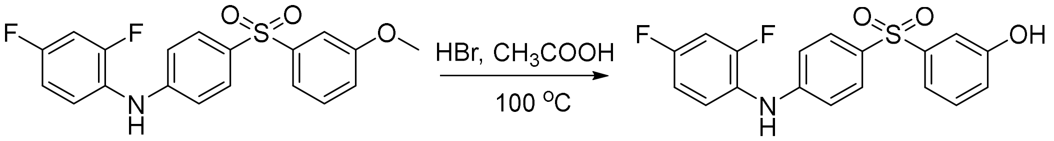

The synthesis method is the same as **11a**, 3-((4-((2,4-difluorophenyl)amino) phenyl) sulfonyl) phenol **22b**[8] (110 mg, 88.0%) as a brown viscous liquid was obtain from **21b**.^{1}H NMR (400 MHz, $CDCl_3$) δ 7.73 (d, *J* = 8.8 Hz, 2H), 7.49 (s, 1H), 7.40 (d, *J* = 7.8 Hz, 1H), 7.33 – 7.26 (m, 2H), 7.01 (dd, *J* = 8.0, 1.6 Hz, 1H), 6.93 – 6.82 (m, 4H), 5.97 (s, 1H). LC-MS(ESI) calcd for $C_{18}H_{13}F_2NO_3S[M+H]^+$ 362.36, found 362.10.

The following diaryl sulfones compounds **23b-d** and **26** were prepared from the intermediate **22b** by a method similar to that for the synthesis of compounds **12a** and **13a**.

(R)-3-(3-((4-((2,4-difluorophenyl)amino)phenyl)sulfonyl)phenoxy)propane-1,2-diol (23b).

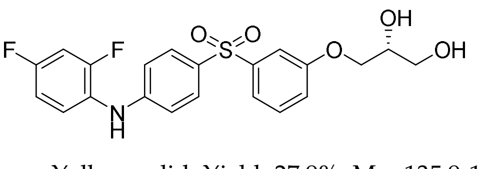

Yellow solid. Yield: 27.9%. Mp: 135.9-136.6 °C. ^{1}H NMR (400 MHz, $CDCl_3$) δ 7.65 (d, *J* = 8.8 Hz, 2H),7.37 (dd, *J* = 12.2, 4.9 Hz, 2H), 7.31 – 7.19 (m, 2H), 6.95 (dd, *J* = 8.2, 1.9 Hz, 1H), 6.88 – 6.74 (m, 4H), 6.03 (s, 1H), 4.11 – 3.82 (m, 3H), 3.69 (ddd, *J* = 16.9, 11.4, 4.6 Hz,

2H).^{13}C NMR (151 MHz, $CDCl_3$) δ 158.2 (dd, *J* = 246.9, 11.1 Hz), 157.8, 154.7 (dd, *J* = 248.8, 12.0 Hz), 147.7, 142.7, 129.8, 129.4, 128.7 (2C), 123.7 (dd, *J* = 12.4, 4.1 Hz), 123.2 (dd, *J* = 9.7, 9.5 Hz), 118.9, 118.3, 113.5 (2C), 111.7, 110.5 (dd, *J* = 22.2, 4.1 Hz), 103.9 (dd, *J* = 25.8, 24.2 Hz), 69.2, 68.4, 62.4.HRMS (EI): calcd for $C_{21}H_{19}F_2NO_5S$ $[M+H]^+$ m/z 435.0952, found 435.0947. HPLC purity: 97.4%, retention time = 13.34 min. $[\alpha]_D^{25}$ = -87.6 (*c* 1.00, CH_2Cl_2).

(S)-3-(3-((4-((2,4-difluorophenyl)amino)phenyl)sulfonyl)phenoxy)propane-1,2-diol (23c).

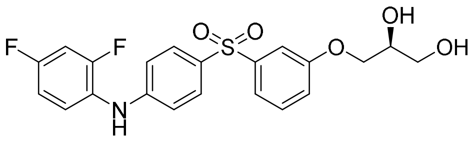

Yellow solid. Yield: 27.9%. Mp: 136.3-136.7 °C. ^{1}H NMR (400 MHz, $CDCl_3$) δ 7.73 (d, *J* = 8.7 Hz, 2H), 7.48 – 7.40 (m, 2H), 7.39 – 7.26 (m, 2H), 7.03 (dd, *J* = 8.2, 1.7 Hz, 1H), 6.95 – 6.81 (m, 4H), 6.05 (s, 1H), 4.19 – 3.96 (m, 3H), 3.78 (ddd, *J* = 17.0, 16.6, 5.3 Hz, 2H).^{13}C NMR (151 MHz, $CDCl_3$) δ 158.9 (dd, *J* = 246.8, 11.0 Hz), 156.5, 154.8 (dd, *J* = 248.6, 11.8 Hz), 148.6, 143.8, 131.1, 130.4, 129.9, 129.8, 124.6 (dd, *J* = 12.7, 4.3 Hz), 124.3 (dd, *J* = 9.7, 9.5 Hz), 120.0, 119.4, 114.5, 112.7, 111.7, 111.5 (dd, *J* = 22.3, 4.2 Hz), 104.80 (dd, *J* = 25.3, 24.2 Hz), 70.2, 69.5, 63.4. HRMS (EI): calcd for $C_{21}H_{19}F_2NO_5S$ $[M+H]^+$ m/z 435.0952, found 435.0951. HPLC purity: 98.4%, retention time = 13.56 min. $[\alpha]_D^{25}$ = +87.6 (*c* 1.00, CH_2Cl_2).

methyl 4-(3-((4-((2,4-difluorophenyl)amino)phenyl)sulfonyl)phenoxy)butanoate (23d)

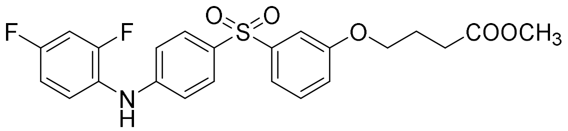

Yellow oily liquid. Yield: 64.7%. Mp: 126.8-127.4 °C. ^{1}H NMR (400 MHz, $CDCl_3$) δ 7.76 (d, *J* = 8.7 Hz, 2H), 7.46 (d, *J* = 7.7 Hz, 1H), 7.42 – 7.27 (m, 3H), 7.02 (dd, *J* = 8.1, 2.1 Hz, 1H), 6.96 – 6.82 (m, 4H), 5.95 (s, 1H), 4.02 (t, *J* = 6.1 Hz, 2H), 3.69 (s, 3H), 2.51 (t, *J* = 7.2 Hz, 2H), 2.21 – 2.01 (m, 2H). ^{13}C NMR (151 MHz, $CDCl_3$) δ 173.5, 159.2 (dd, *J* = 246.7, 11.3 Hz), 159.2, 155.7 (dd, *J* = 248.4, 12.3 Hz), 148.6, 143.7, 131.2, 130.3, 129.7 (2C), 124.6 (dd, *J* = 9.6, 2.2 Hz), 124.3 (dd, *J* = 12.4, 4.1 Hz), 119.5, 119.3, 114.5 (2C), 112.5, 111.5 (dd, *J* = 22.2, 4.0 Hz), 104.9 (dd, *J* = 25.9, 24.1 Hz), 67.2, 51.7, 30.4, 24.4. HRMS (EI): calcd for $C_{23}H_{21}F_2NO_5S$, $[M]^+$: m/z: 461.1008; found 461.1110.

4. -(3-((4-((2,4-difluorophenyl)amino)phenyl)sulfonyl)phenoxy)butanoic acid (26).

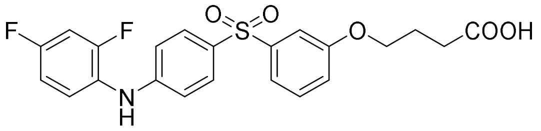

Yellow solid. Yield: 24.4%. Mp: 132.6-133.1 °C. ^{1}H NMR (400 MHz, DMSO-d_6) δ 12.17 (s, 1H), 8.69 (s, 1H), 7.74 (d, *J* = 8.8 Hz, 2H), 7.49 (t, *J* = 8.0 Hz, 1H), 7.44 – 7.33 (m, 4H), 7.22 – 7.17 (m, 1H), 7.14 – 7.06 (m, 1H), 6.85 (d, *J* = 8.6 Hz, 2H), 4.05 (t, *J* = 6.3 Hz, 2H), 2.39 (t, *J* = 7.3 Hz, 2H), 1.95 (p, *J* = 5.9, 5.2 Hz, 2H).^{13}C NMR (151 MHz, DMSO-d_6) δ 174.7, 159.4, 159.3 (dd, *J* = 244.1, 11.2 Hz), 156.4 (dd, *J* = 248.3, 12.5 Hz), 150.3, 144.3, 131.3, 129.9 (2C), 129.3, 127.1 (dd, *J* = 9.6, 2.1 Hz), 124.9 (dd, *J* = 12.1, 2.9 Hz), 119.6, 119.3, 114.1 (2C), 112.6, 112.4 (dd, *J* = 21.9, 3.4 Hz), 105.5 (dd, *J* = 26.6, 24.5 Hz), 67.8, 30.8, 24.6.HRMS (EI): calcd for $C_{22}H_{19}F_2NO_5S$ $[M+H]^+$ m/z 447.0952, found 447.0951. HPLC purity: 97.3%, retention time = 13.58 min.

The intermediates **19a-22a** were prepared from the intermediate **17** by a method similar to that for the synthesis of the intermediates **19b-22b** from the intermediate **18**. The following diaryl sulfoxides compound**25** was prepared by a method similar to that for the

synthesis of compound **26.** The intermediates **17-22**[6-11] were prepared according to the corresponding literature procedure.

4-((3-methoxyphenyl)thio)aniline (19a[9]).

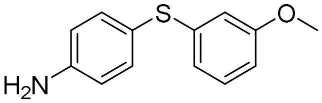

Yellow solid. Yield: 92.7%.^{1}H NMR (400 MHz, $CDCl_3$) δ 8.28 (dd, *J* = 71.2, 8.8 Hz, 1H), 7.77 (dd, *J* = 17.6, 8.8 Hz, 1H), 7.46 – 7.28 (m, 2H), 7.23 – 7.16 (m, 1H), 7.07 (d, *J* = 7.8 Hz, 1H), 7.00 – 6.89 (m, 1H), 6.86 (d, *J* = 8.6 Hz, 1H), 3.81 (s, 3H). LC-MS(ESI) calcd for $C_{13}H_{13}NOS$ $[M+H]^+$ 232.31, found 232.20.

2,4-difluoro-N-(4-((3-methoxyphenyl)thio)phenyl)aniline (21a[10]).

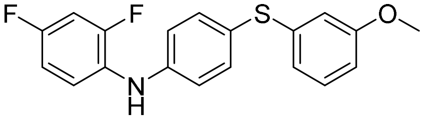

Yellow viscous liquid. Yield: 62.3%. ^{1}H NMR (400 MHz, $CDCl_3$) δ 7.59 – 7.44 (m, 2H), 7.34 (t, *J* = 7.8 Hz, 1H), 7.29 (dd, *J* = 9.2, 5.8 Hz, 1H), 7.24 (dd, *J* = 4.4, 2.0 Hz, 1H), 7.11 (d, *J* = 4.0 Hz, 1H), 6.98-6.88 (m, 4H), 6.88 – 6.80 (m, 1H), 5.90 (s, 1H), 3.83 (s, 3H). LC-MS(ESI) calcd for $C_{19}H_{15}F_2NOS$ $[M+H]^+$ 344.39, found 344.15.

3-((4-((2,4-difluorophenyl)amino)phenyl)thio)phenol (22a[11]).

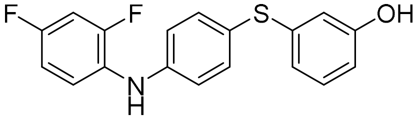

Brown viscous liquid. Yield: 57.2%. ^{1}H NMR (400 MHz, $CDCl_3$) δ 7.41 – 7.26 (m, 4H), 7.12 – 7.06 (m, 1H), 6.96 – 6.73 (m, 5H), 6.62 – 6.57 (m, 1H), 5.67 (s, 1H). LC-MS(ESI) calcd for $C_{18}H_{13}F_2NOS$ $[M+H]^+$ 330.36, found 330.15.

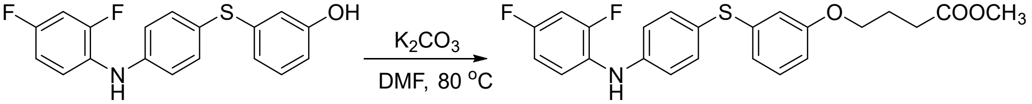

The synthesis method is the same as **12a**, methyl 4-(3-((4-((2,4-difluorophenyl)amino) phenyl)thio)phenoxy)butanoate **23a** (37 mg, 35.6%) was obtain as a brown viscous liquid from 3-((4-((2,4-difluorophenyl) amino) phenyl) thio)phenol **22a**. ^{1}H NMR (400 MHz, $CDCl_3$) δ 7.40 – 7.35 (m, 1H), 7.30 (td, *J* = 9.2, 5.6 Hz, 2H), 7.14 (t, *J* = 8.0 Hz, 1H), 6.98 – 6.94 (m, 2H), 6.93 – 6.80 (m, 2H), 6.79 – 6.73 (m, 1H), 6.73 – 6.70 (m, 1H), 6.69-6.63 (m, 1H), 5.67 (s, 1H), 3.94 (t, *J* = 6.0 Hz, 2H), 3.67 (s, 3H), 2.50 (t, *J* = 7.2 Hz, 2H), 2.11 – 2.01 (m, 2H). LC-MS(ESI) calcd for $C_{23}H_{21}F_2NO_3S$ $[M+H]^+$ 430.48, found 430.20.

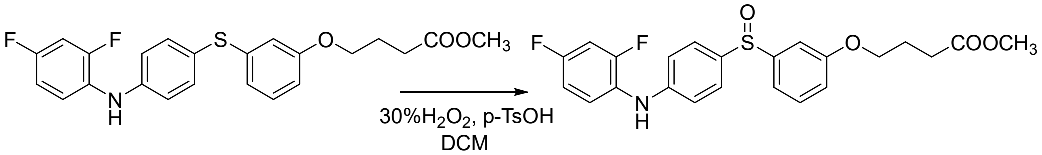

methyl 4-(3-((4-((2,4-difluorophenyl)amino) phenyl)thio)phenoxy)butanoate **23a** (37 mg, 0.09 mmol) was dissolved in dichloromethane (10 mL).Then 30% H_2O_2 (3.38 mL, 0.21 mmol) and p-toluenesulfonic acid (34 mg, 0.20 mmol) were added. After stirred for 10 h at room temperature, the reaction solution was quenched with the sodium sulfite solution and extracted three times with dichloromethane. Then combined organic layers were

washed once with brine and dried over Na_2SO_4, filtered, and the solvent was removed by rotary evaporation to give the crude methyl 4-(3-((4-((2,4-difluorophenyl)amino)phenyl)sulfinyl)phenoxy)butanoate **24** (30 mg, 78.2%) as a yellow oily liquid. LC-MS(ESI) calcd for $C_{23}H_{21}F_2NO_4S$ $[M+H]^+$ 446.48, found 446.20.

4-(3-((4-((2,4-difluorophenyl) amino) phenyl)sulfinyl)phenoxy)butanoic acid (25).

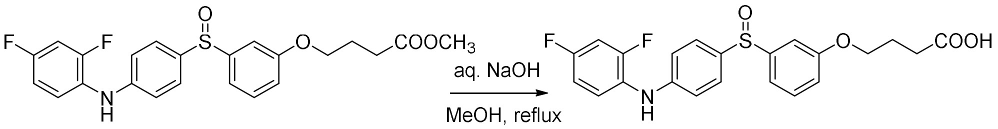

The synthesis method is the same as **13a**, 4-(3-((4-((2,4-difluorophenyl) amino) phenyl)sulfinyl)phenoxy)butanoic acid **25** (13 mg, 44.8%) as a yellow solid was obtain from methyl 4-(3-((4-((2,4-difluorophenyl)amino)phenyl)sulfinyl)phenoxy)butanoate **24** (30 mg, 0.07 mmol). Mp: 126.1-126.7 °C. ^{1}H NMR (400 MHz, DMSO-d_6) δ 8.58 (s, 1H), 7.55 – 7.26 (m, 5H), 7.19 – 6.96 (m, 4H), 6.86 (d, J = 6.5 Hz, 2H), 3.99 (s, 2H), 2.05 (s, 2H), 1.87 (s, 2H). ^{13}C NMR (151 MHz, DMSO-d_6) δ 177.1, 159.8, 158.6 (dd, J = 242.8, 11.8 Hz), 155.9 (dd, J = 247.7, 12.4 Hz), 148.4, 148.3, 134.2, 130.9, 127.0 (2C), 125.9 (dd, J = 13.6, 10.6 Hz), 117.2, 116.1, 115.0, 114.8 (2C), 112.2 (dd, J = 19.8, 2.6 Hz), 109.5, 105.3 (dd, J = 25.3, 25.1 Hz), 68.6, 34.0, 26.2. HRMS (EI): calcd for $C_{22}H_{19}F_2NO_4S$ $[M+H]^+$ m/z 431.1003, found 431.1169. HPLC purity: 98.3%, retention time = 14.07 min.

References.

[1] E. Kozma G, et al. Bioorthogonal double-fluorogenic siliconrhodamine probes for intracellular super-resolution microscopy. *Chem Commun* **2017**, *53* (50), 6696-6699.

[2] Lee Belding, et al. Phase-transfer catalysis via a proton sponge: A bifunctional role for biscyclopropenimine. *J Org Chem* **2016**, *81* (2), 553-558.

[3] Solveigh C. Koeberle, et al. Design, synthesis, and biological evaluation of novel disubstituted dibenzosuberones as highly potent and selective inhibitors of p38 mitogen activated protein kinase. *J Med Chem* **2012**, *55*, 5868-5877.

[4] Robert H.E., et al. On the necessity of nucleobase protection for 2-thiouracil for fmoc-based pseudo-complementary peptide nucleic acid oligomer synthesis. *J Org Chem* **2019**, *84* (21), 13252-13261.

[5] Sugiyama Toru, et al. PNA monomers fully compatible with standard fmoc-based solid-phase synthesis of pseudo complementary PNA. *Bioorg Med Chem Lett* **2017**, *27* (15), 3337-3341.

[6] Clark Robin D, et al. Discovery and SAR development of 2-(phenylamino) imidazolines as postacyclin receptor antagonists. *Bioorg Med Chem Lett* **2004**, *14* (4), 1053-1056.

[7] Cyrus J. Ohnmacht, et al. N-aryl-3,3,3-trifluoro-2-hydroxy-2-methylpropanamides: K(ATP) potassium channel openers. modifications on the western region. *J Med Chem* **1996**, *39* (23), 4592-4601.

[8] Thomas Otzen, et al. Folate-synthesizing enzyme system as target for development of inhibitors and inhibitor combinations against candida albicans - synthesis and biological activity of new 2,4-diaminopyrimidines and 4′-substituted 4-aminodiphenyl sulfones. *J Med Chem* **2004**, *47* (1), 240-253.

[9] Daoshan Yang, et al. Metal-free iodine-catalyzed direct arylthiation of substituted anilines with thiols. *J Org Chem* **2015**, *80* (12), 6083-6092.

[10] Jie Li, et al. Design, synthesis and antitumor activity of novel cis-furoquinoline derivatives. *Lett Drug Des Discov* **2012**, *9* (4), 379-388.

[11] M. Shahjahan Kabir, et al. A very active Cu-catalytic system for the synthesis of aryl, heteroaryl, and vinyl sulfides. *J Org Chem* **2010**, *75* (11), 3626-3643.

Table S1. Glide docking scores of compounds.

Compounds	Docking Score kcal/mol	Glide gscore kcal/mol
**12a**	-9.856	-9.856
**12c**	-9.229	-9.229
**12d**	-9.664	-9.664
**12f**	-9.194	-9.194
**13a**	-10.564	-10.566
**13c**	-9.027	-9.028
**14a**	-10.175	-10.175
**12b**	-9.962	-9.962
**12e**	-9.501	-9.501
**13b**	-10.969	-10.970
**23b**	-6.790	-6.790
**23c**	-6.982	-6.982
**26**	-6.826	-6.828
**25**	-8.125	-8.127

Figure S1. The spectra of ^{1}H NMR, ^{13}C NMR and HRMS (EI) of representative compounds.

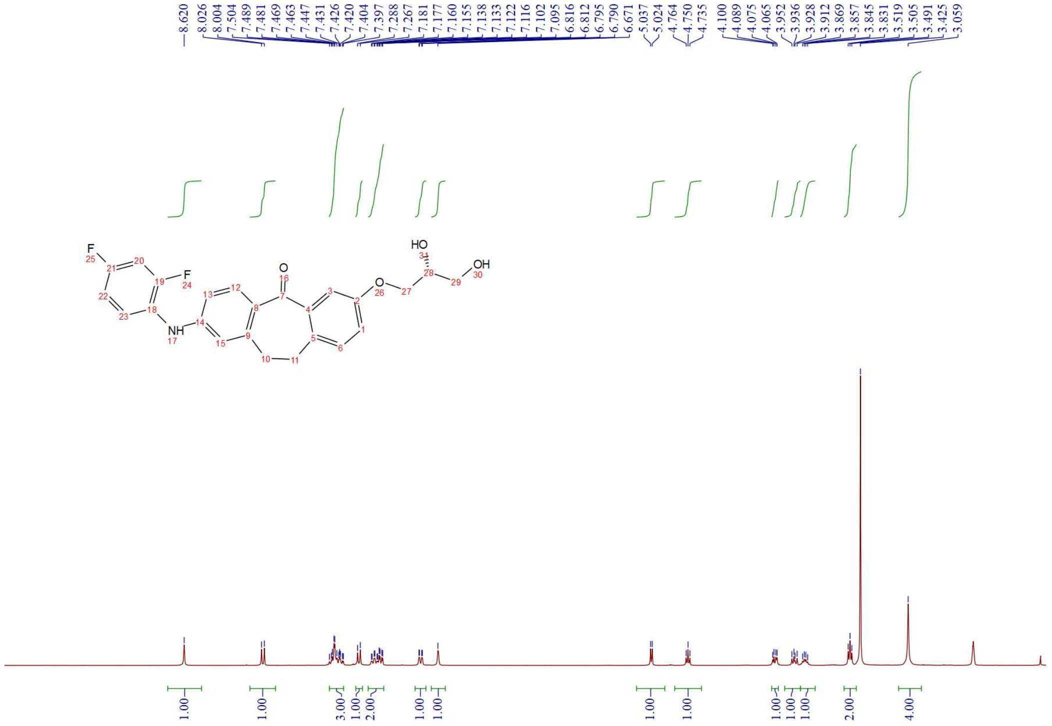
8.620
8.026
8.004
7.504
7.489
7.481
7.469
7.463
7.447
7.431
7.426
7.420
7.404
7.397
7.288
7.267
7.181
7.177
7.160
7.155
7.138
7.133
7.122
7.116
7.102
7.095
6.816
6.812
6.795
6.790
6.671
5.037
5.024
4.764
4.750
4.735
4.100
4.089
4.075
4.065
3.952
3.936
3.928
3.912
3.869
3.857
3.845
3.831
3.519
3.505
3.491
3.425
3.059
F
F
NH
O
O
HO
OH
1.00
1.00
3.00
1.00
2.00
1.00
1.00
1.00
1.00
1.00
1.00
1.00
2.00
4.00

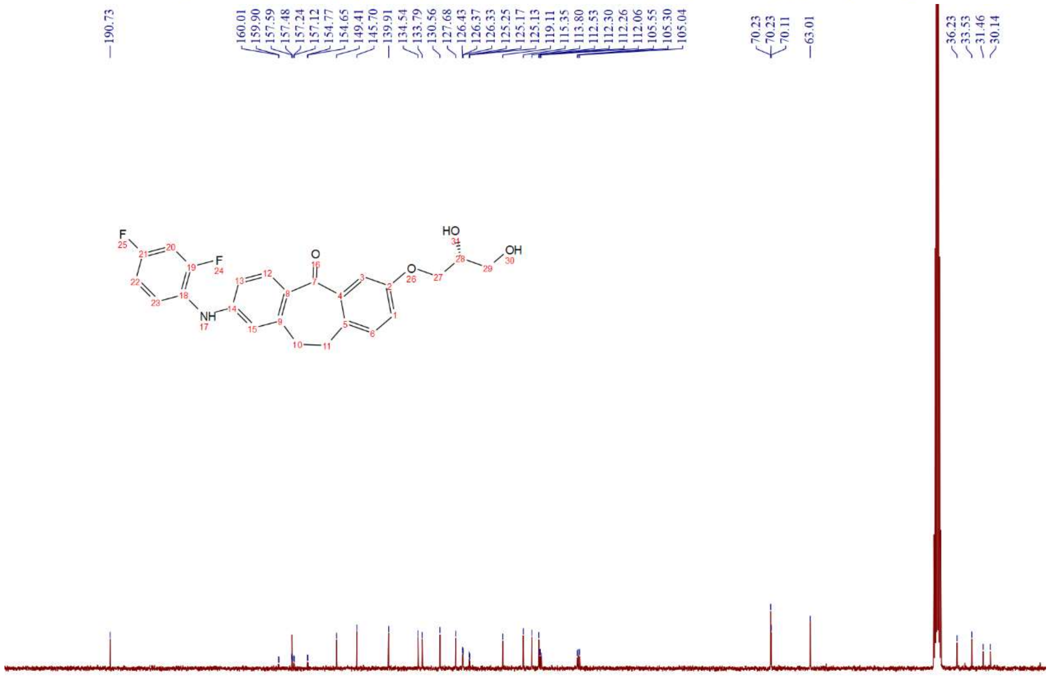
190.73
160.01
159.90
157.59
157.48
157.24
157.12
154.77
154.65
149.41
145.70
139.91
134.54
133.79
130.56
127.68
126.43
126.37
126.33
125.25
125.17
125.13
119.11
115.35
113.80
112.53
112.30
112.26
112.06
105.55
105.30
105.04
70.23
70.23
70.11
63.01
36.23
33.53
31.46
30.14
F
F
NH
O
O
HO
OH

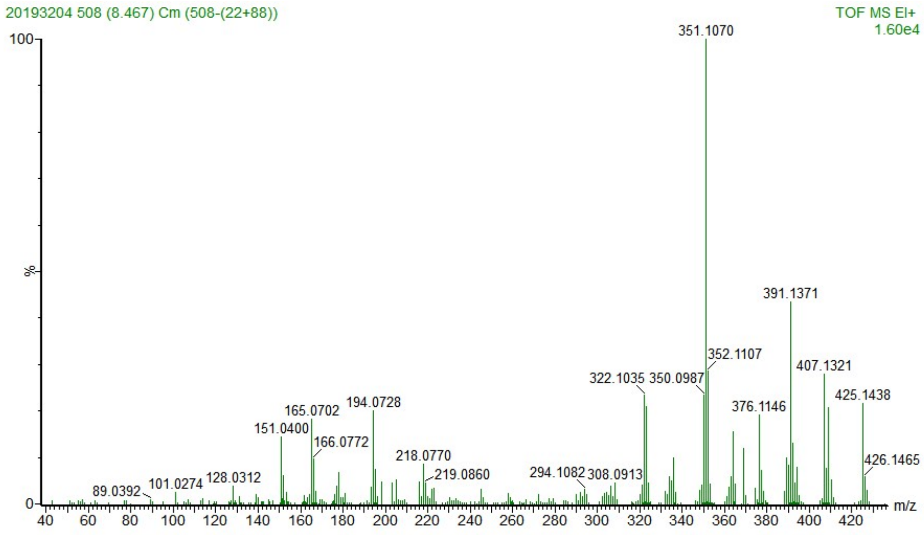

(A) ^{1}H NMR, ^{13}C NMR and HRMS (EI) spectrums of Compound **12a**.

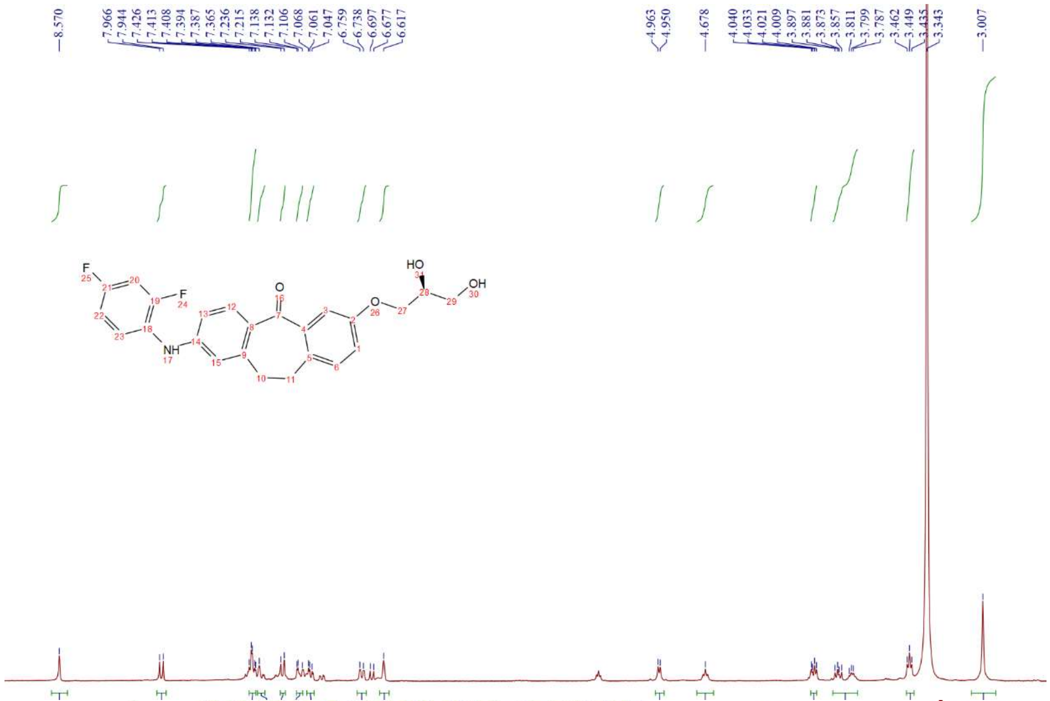

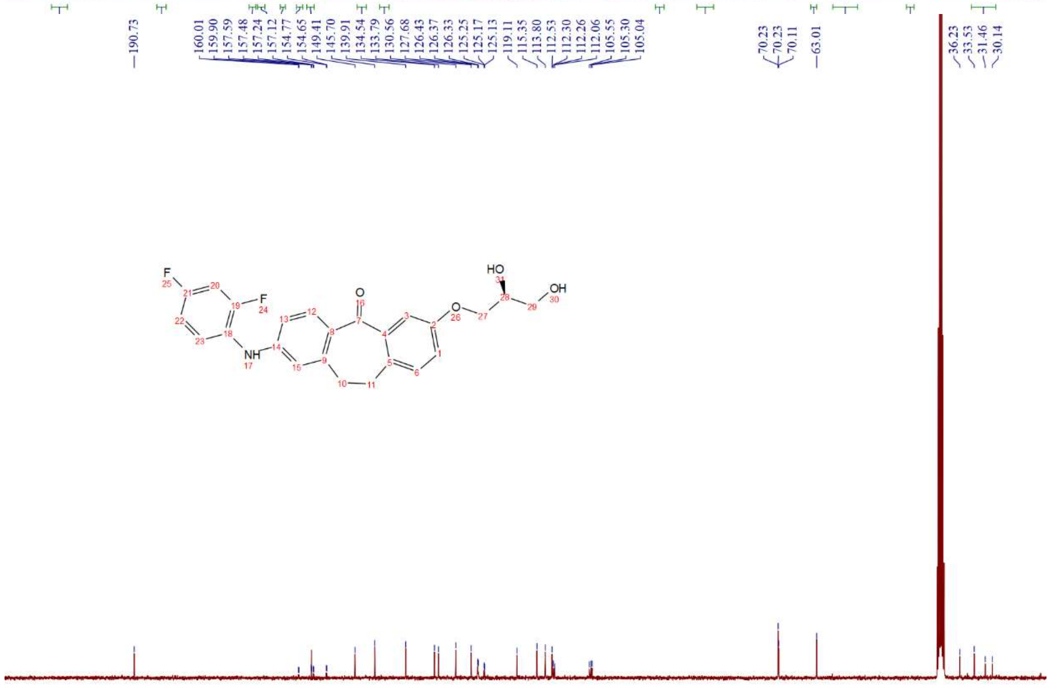

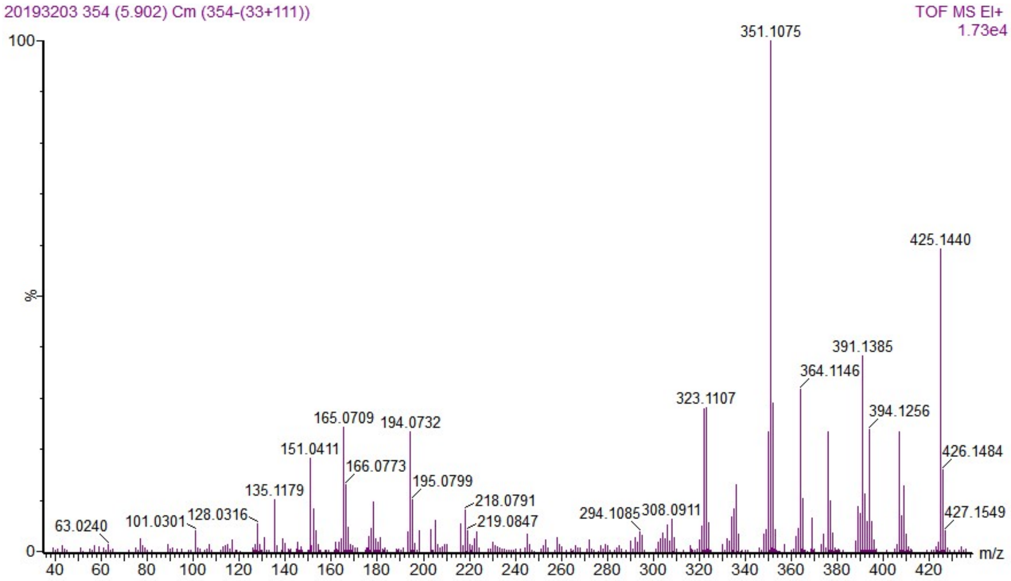

(B) ^{1}H NMR, ^{13}C NMR and HRMS (EI) spectrums of Compound **12c**.

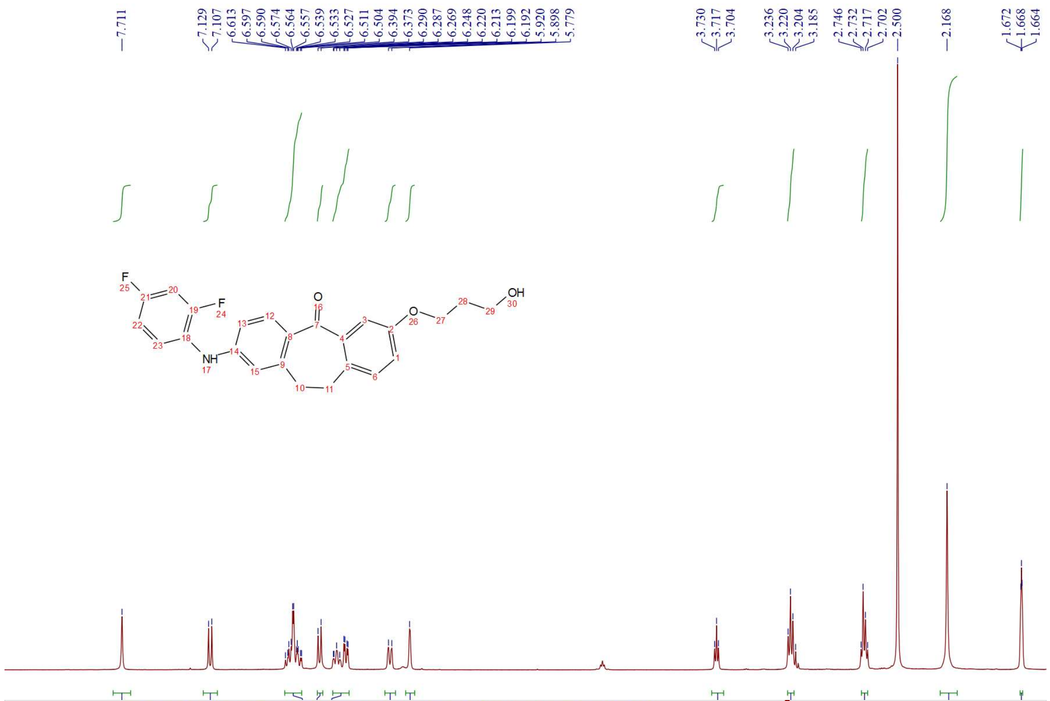
7.711
7.129
7.107
6.613
6.597
6.590
6.574
6.564
6.557
6.539
6.533
6.527
6.511
6.504
6.394
6.373
6.290
6.287
6.269
6.248
6.220
6.213
6.199
6.192
5.920
5.898
5.779
3.730
3.717
3.704
3.236
3.220
3.204
3.185
2.746
2.732
2.717
2.702
2.500
2.168
1.672
1.668
1.664
F
OH
NH
O

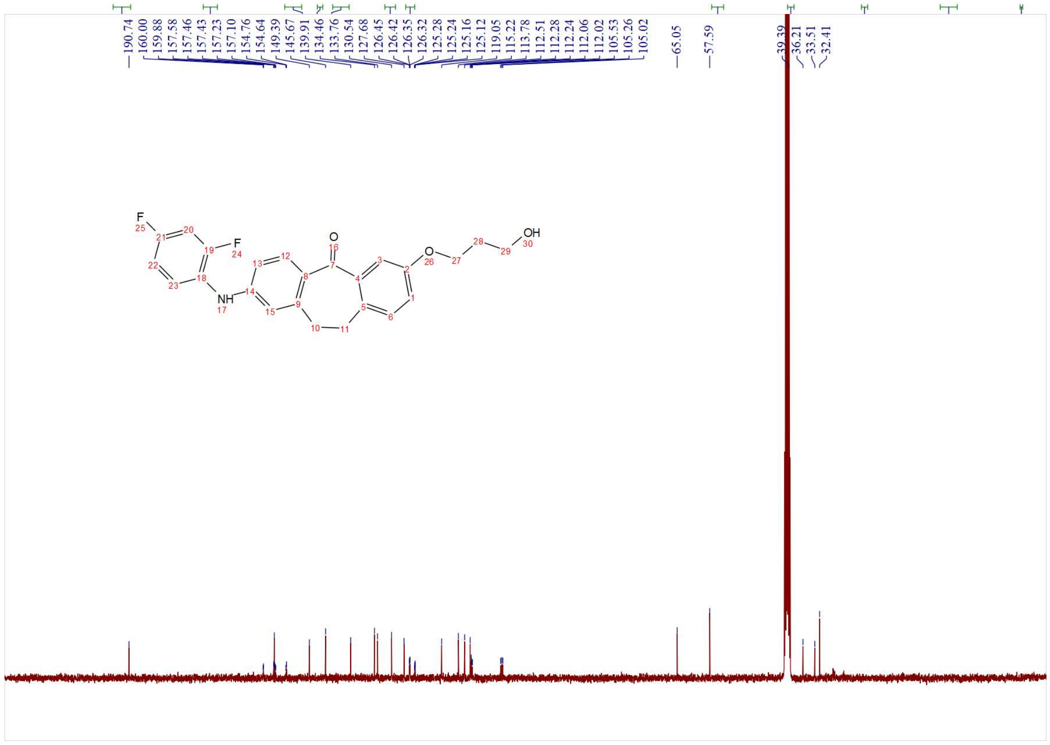
190.74
160.00
159.88
157.58
157.46
157.43
157.23
157.10
154.76
154.64
149.39
145.67
139.91
134.46
133.76
130.54
127.68
126.45
126.42
126.35
126.32
125.28
125.24
125.16
125.12
119.05
115.22
113.78
112.51
112.28
112.24
112.06
112.02
105.53
105.26
105.02
65.05
57.59
39.39
36.21
33.51
32.41
F
OH
NH
O

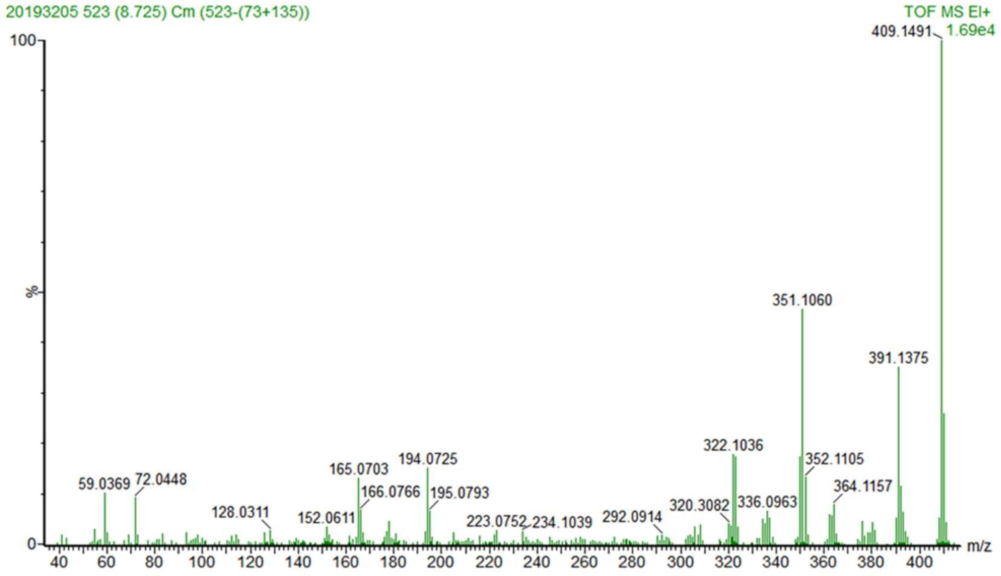

(C) ^{1}H NMR, ^{13}C NMR and HRMS (EI) spectrums of Compound **12d**.

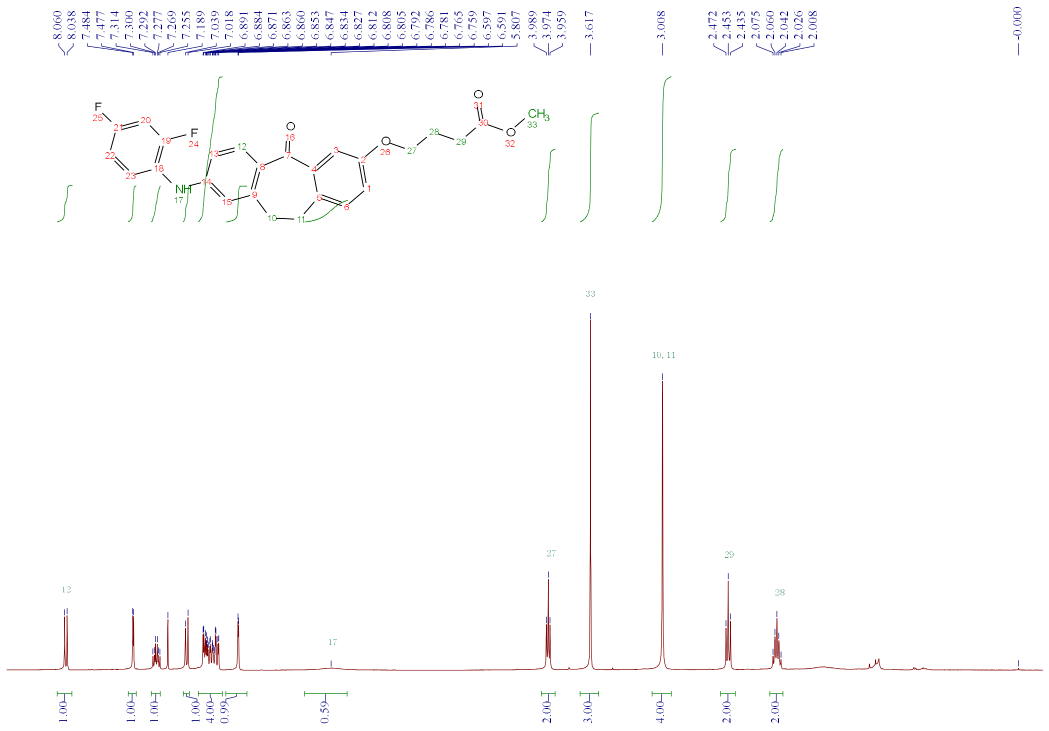

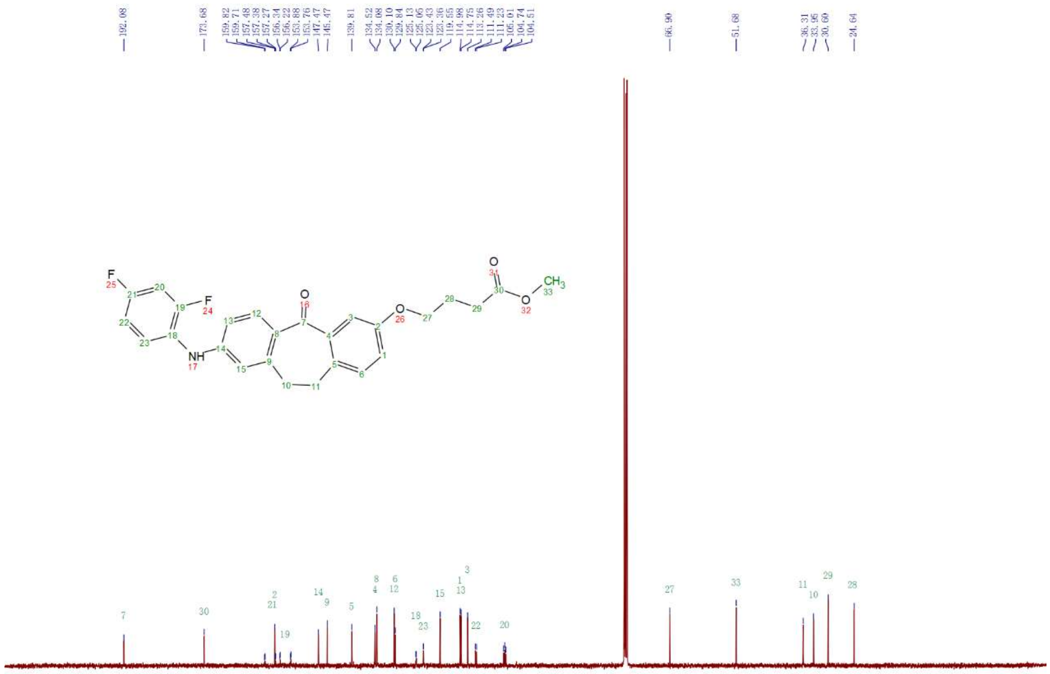

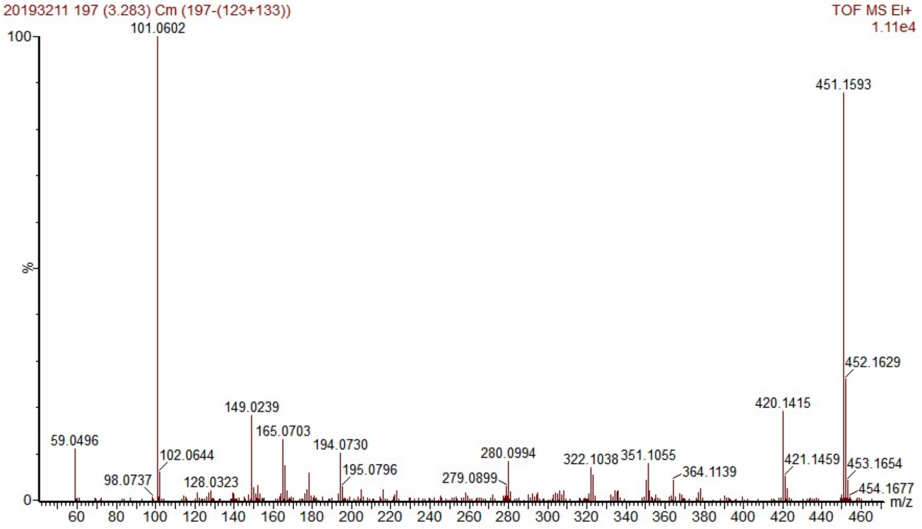

(D) ^{1}H NMR, ^{13}C NMR and HRMS (EI) spectrums of Compound **12f**.

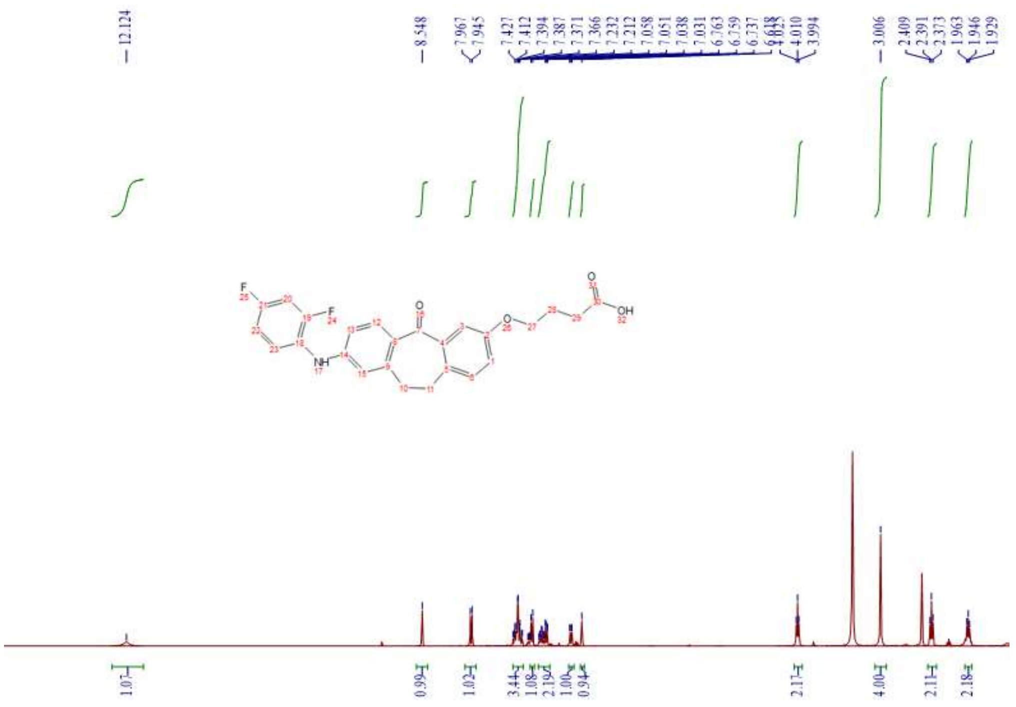
12.124
8.548
7.967
7.945
7.427
7.412
7.394
7.387
7.371
7.366
7.232
7.212
7.058
7.051
7.038
7.031
6.763
6.759
6.737
4.010
3.994
3.006
2.409
2.391
2.373
1.963
1.946
1.929
F
NH
O
OH
1.07
0.99
1.02
3.44
1.08
2.19
1.00
0.94
2.17
4.00
2.11
2.18

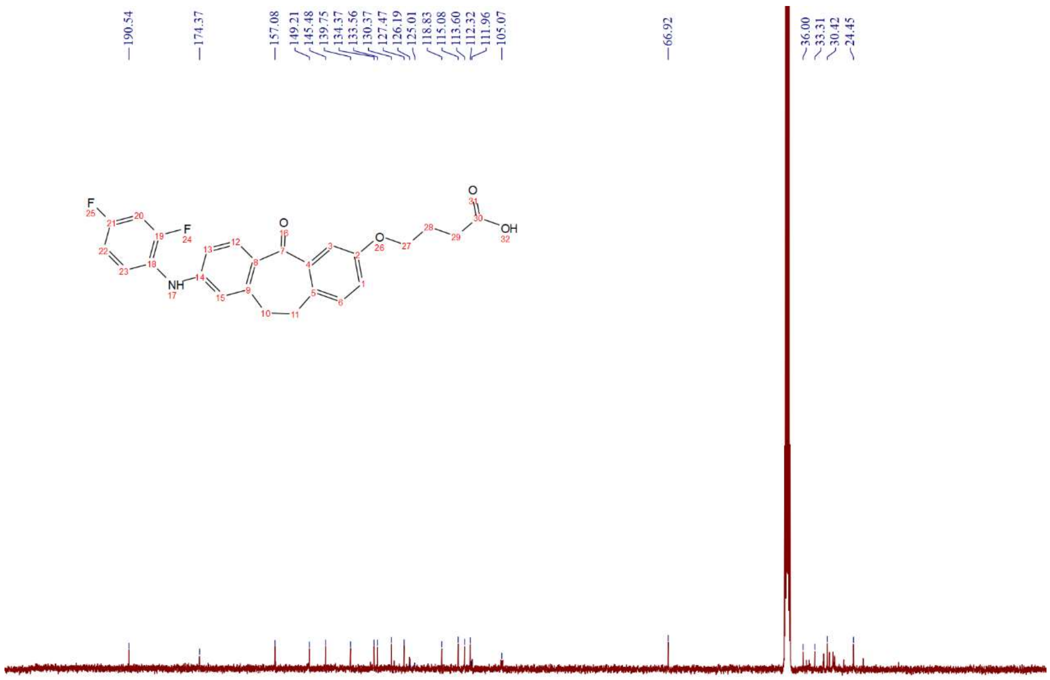
190.54
174.37
157.08
149.21
145.48
139.75
134.37
133.56
130.37
127.47
126.19
125.01
118.83
115.08
113.60
112.32
111.96
105.07
66.92
36.00
33.31
30.42
24.45
F
NH
O
OH

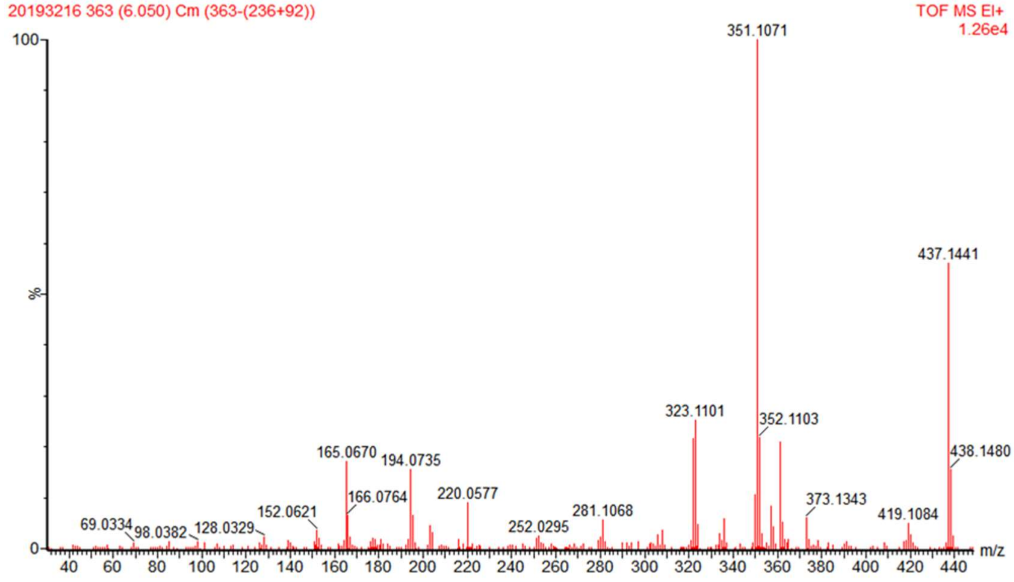

(E) ^{1}H NMR, ^{13}C NMR and HRMS (EI) spectrums of Compound **13a**.

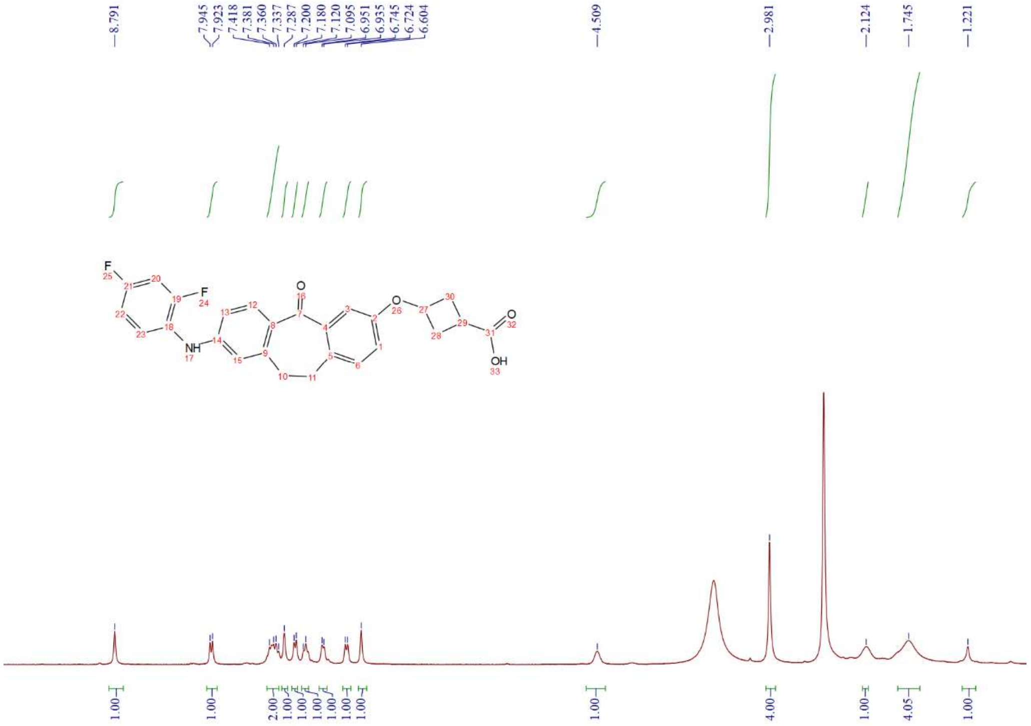

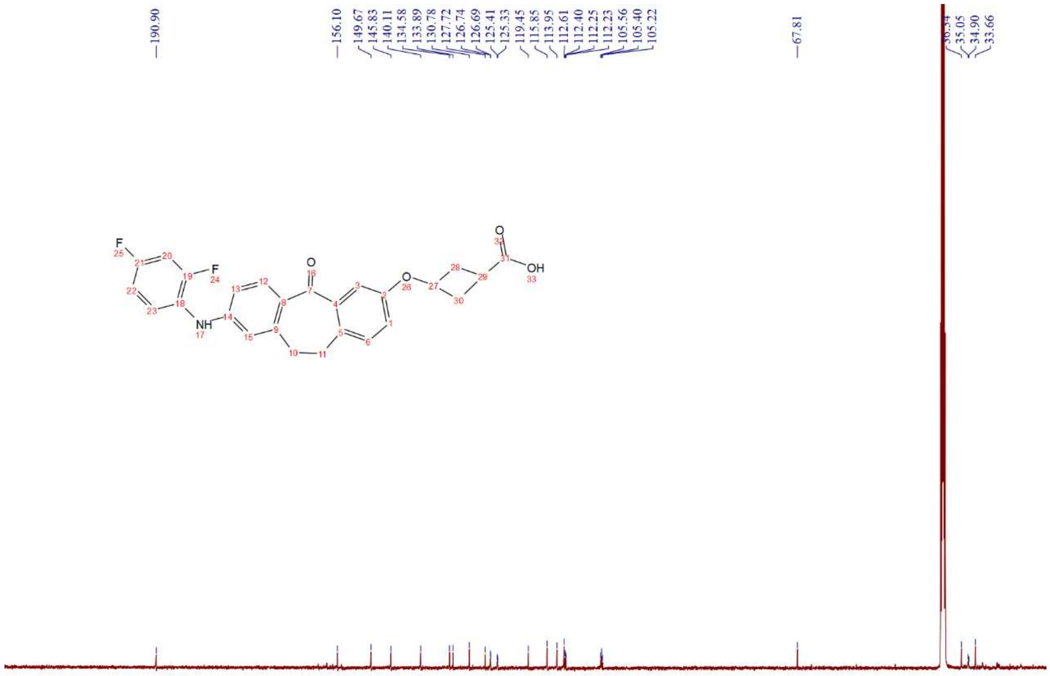

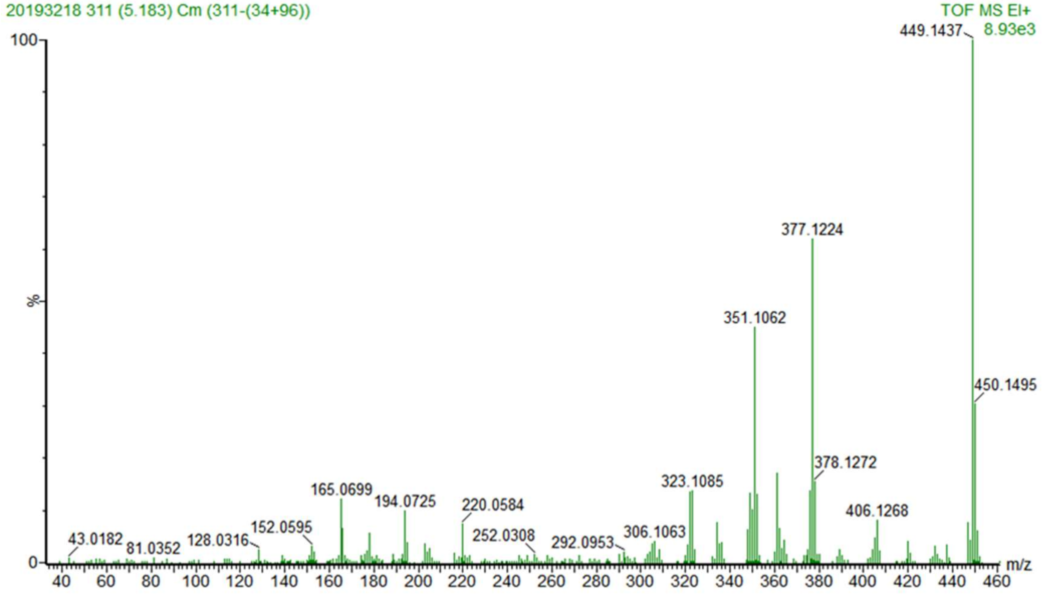

(F) ^{1}H NMR, ^{13}C NMR and HRMS (EI) spectrums of Compound **13c**.

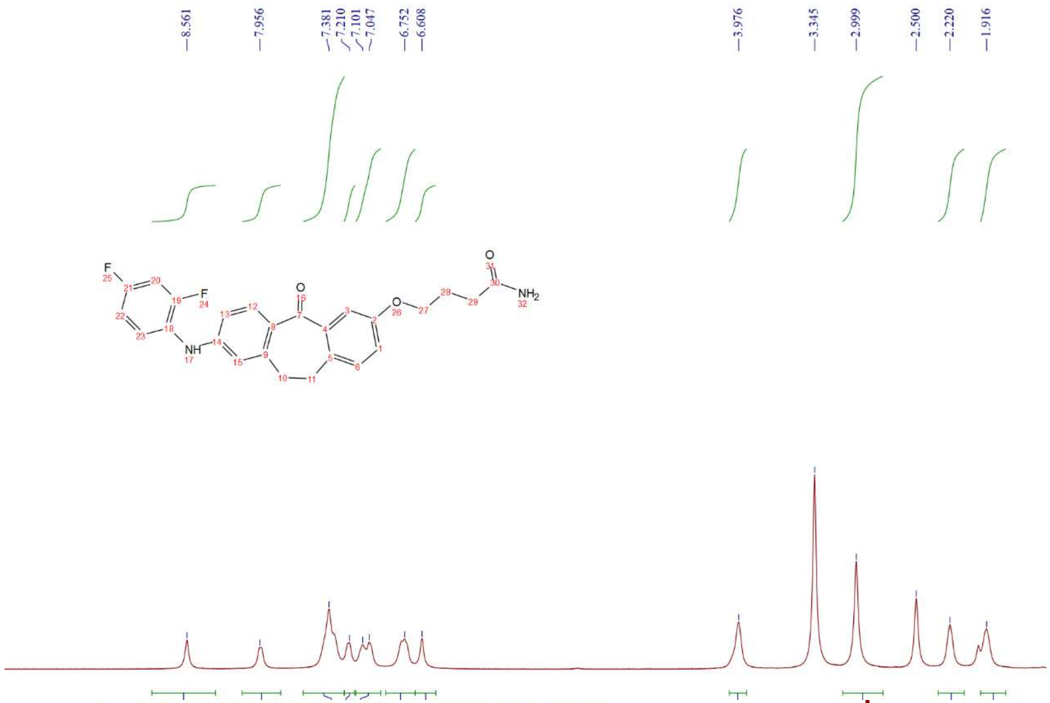
8.561
7.956
7.381
7.210
7.101
7.047
6.752
6.608
3.976
3.345
2.999
2.500
2.220
1.916
F
F
O
O
NH
NH2

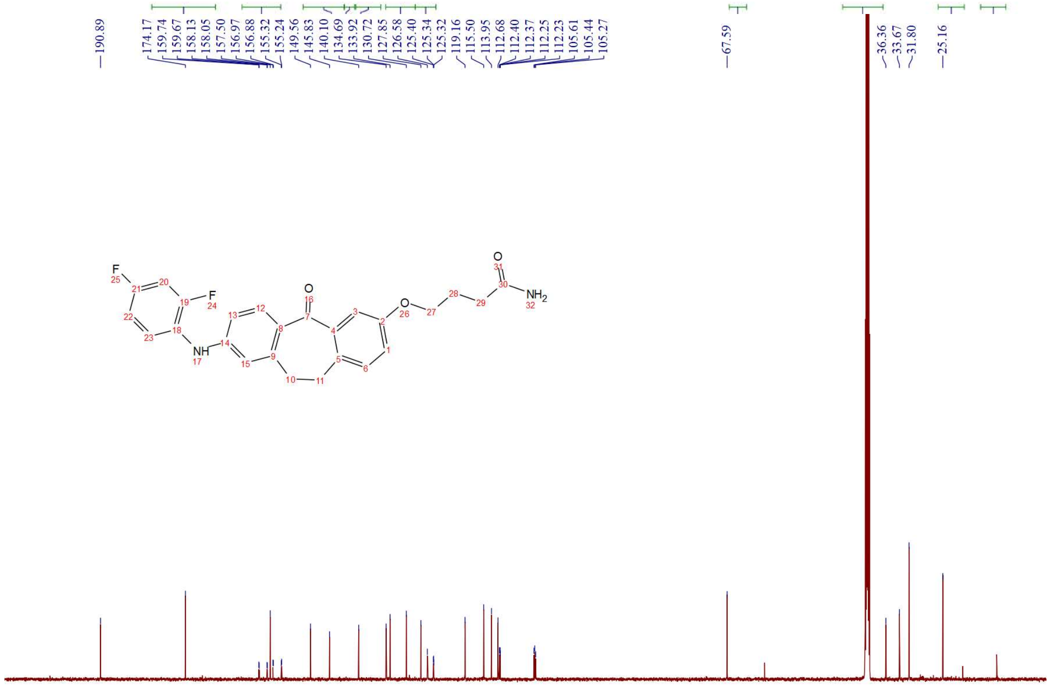
190.89
174.17
159.74
159.67
158.13
158.05
157.50
156.97
156.88
155.32
155.24
149.56
145.83
140.10
134.69
133.92
130.72
127.85
126.58
125.40
125.34
125.32
119.16
115.50
113.95
112.68
112.40
112.37
112.25
112.23
105.61
105.44
105.27
67.59
36.36
33.67
31.80
25.16
F
F
O
O
NH
NH2

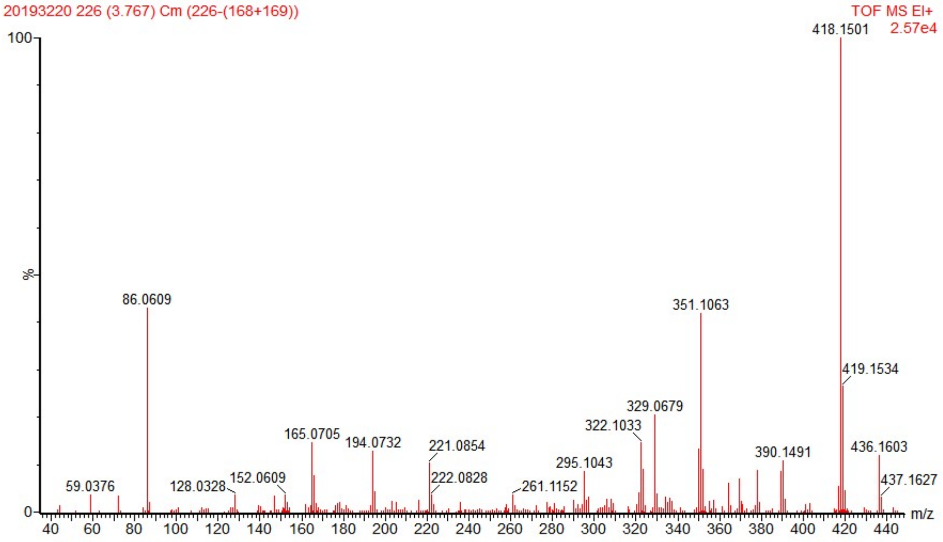

(G) ^{1}H NMR, ^{13}C NMR and HRMS (EI) spectrums of Compound **14a**.

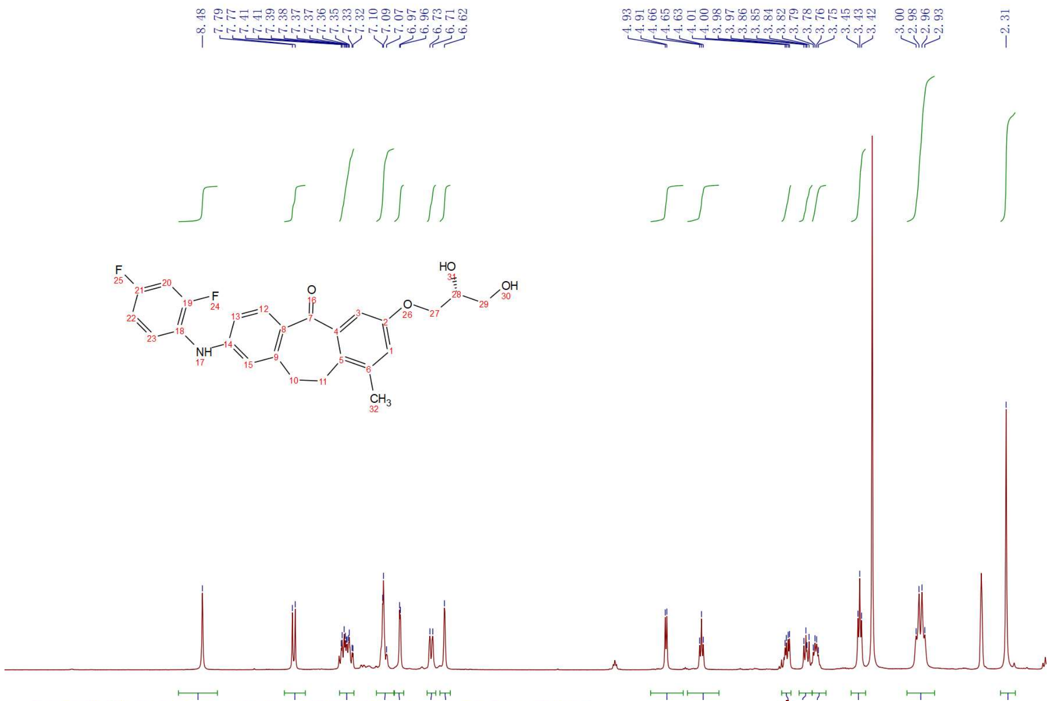
8.48
7.79
7.77
7.41
7.41
7.39
7.38
7.37
7.36
7.35
7.33
7.32
7.10
7.09
7.07
6.97
6.96
6.73
6.71
6.62
4.93
4.91
4.66
4.65
4.63
4.01
4.00
3.98
3.97
3.86
3.85
3.84
3.82
3.79
3.78
3.76
3.75
3.45
3.43
3.42
3.00
2.98
2.96
2.93
2.31
F
HO
OH
NH
O
CH3

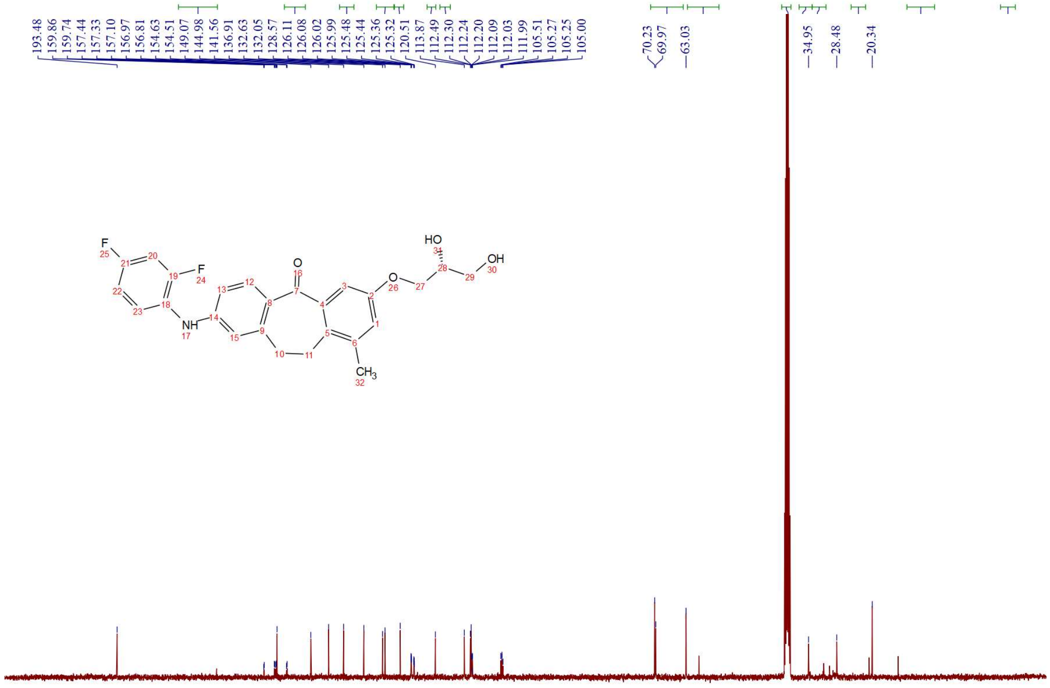
193.48
159.86
159.74
157.44
157.33
157.10
156.97
156.81
154.63
154.51
149.07
144.98
141.56
136.91
132.63
132.05
128.57
126.11
126.08
126.02
125.99
125.48
125.44
125.36
125.32
120.51
113.87
112.49
112.30
112.24
112.20
112.09
112.03
111.99
105.51
105.27
105.25
105.00
70.23
69.97
63.03
34.95
28.48
20.34
F
HO
OH
NH
O
CH3

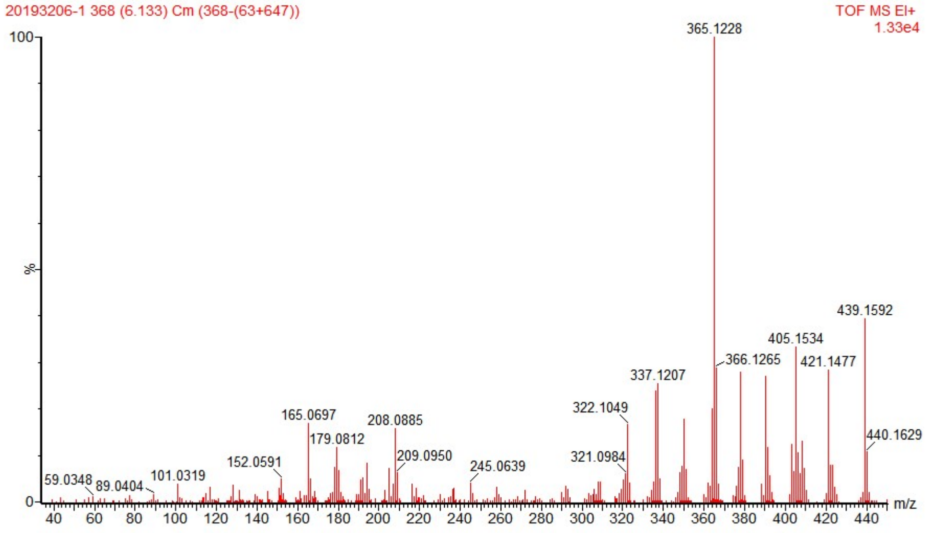

(H) ^{1}H NMR, ^{13}C NMR and HRMS (EI) spectrums of Compound **12b**.

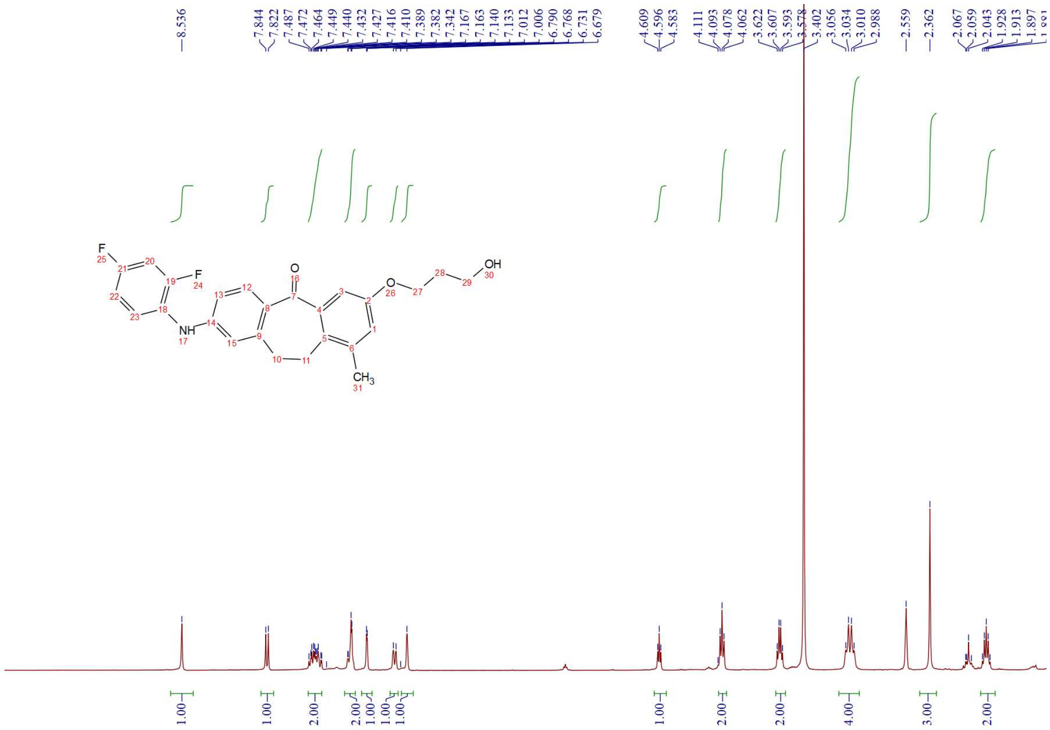

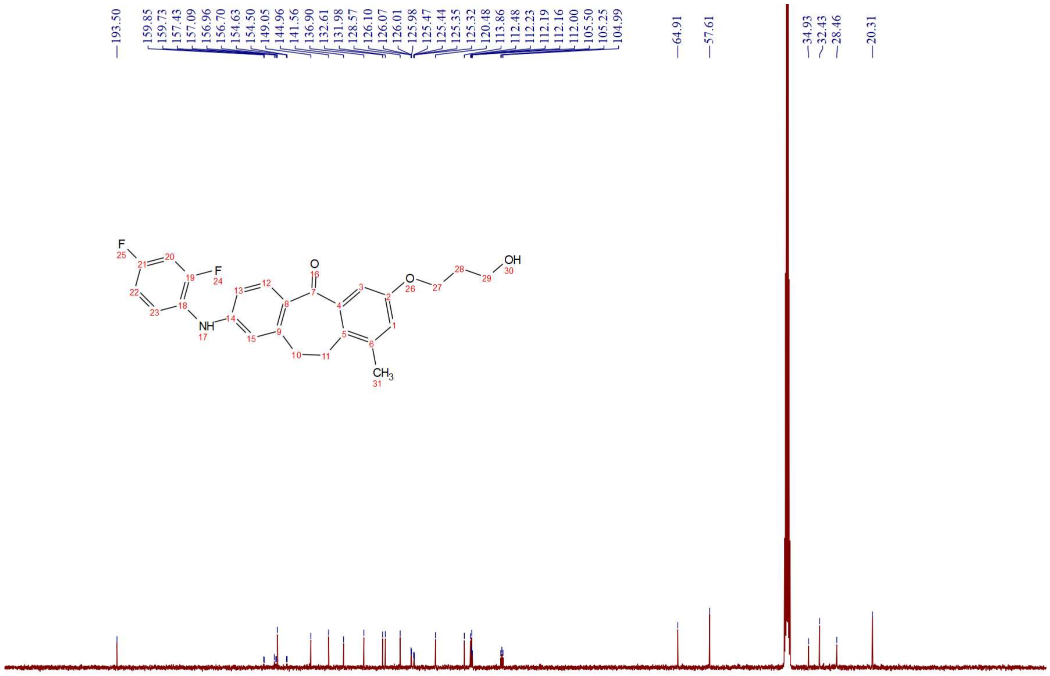

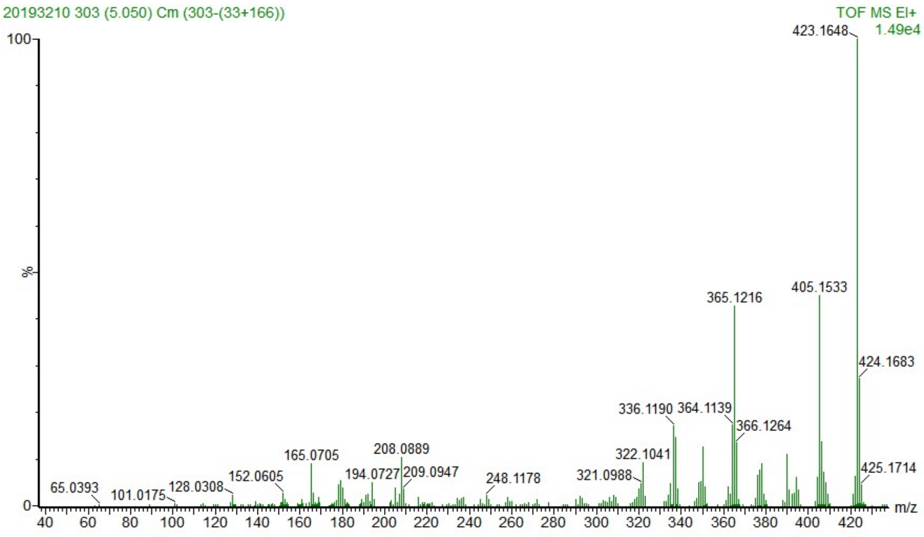

(I) ^{1}H NMR, ^{13}C NMR and HRMS (EI) spectrums of Compound **12e**.

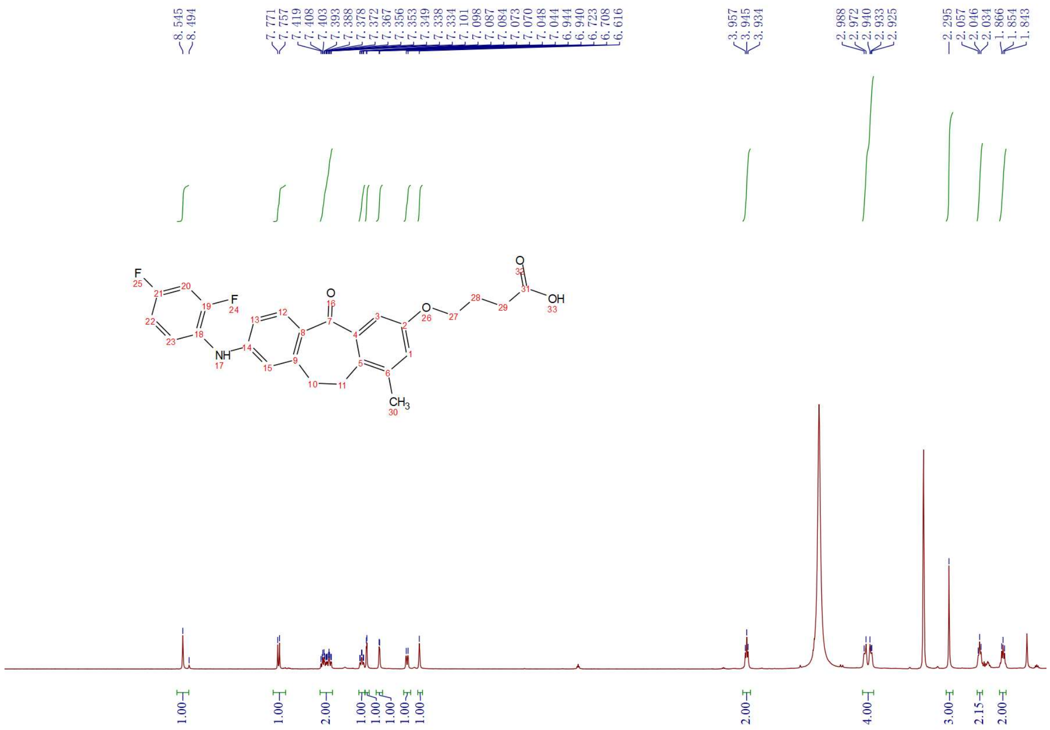
8.545
8.494
7.771
7.757
7.419
7.408
7.403
7.393
7.388
7.378
7.372
7.367
7.356
7.353
7.349
7.338
7.334
7.101
7.098
7.087
7.084
7.073
7.070
7.048
7.044
6.944
6.940
6.723
6.708
6.616
3.957
3.945
3.934
2.988
2.972
2.940
2.933
2.925
2.295
2.057
2.046
2.034
1.866
1.854
1.843
F
NH
O
OH
CH3
1.00
1.00
2.00
1.00
1.00
1.00
1.00
1.00
2.00
4.00
3.00
2.15
2.00

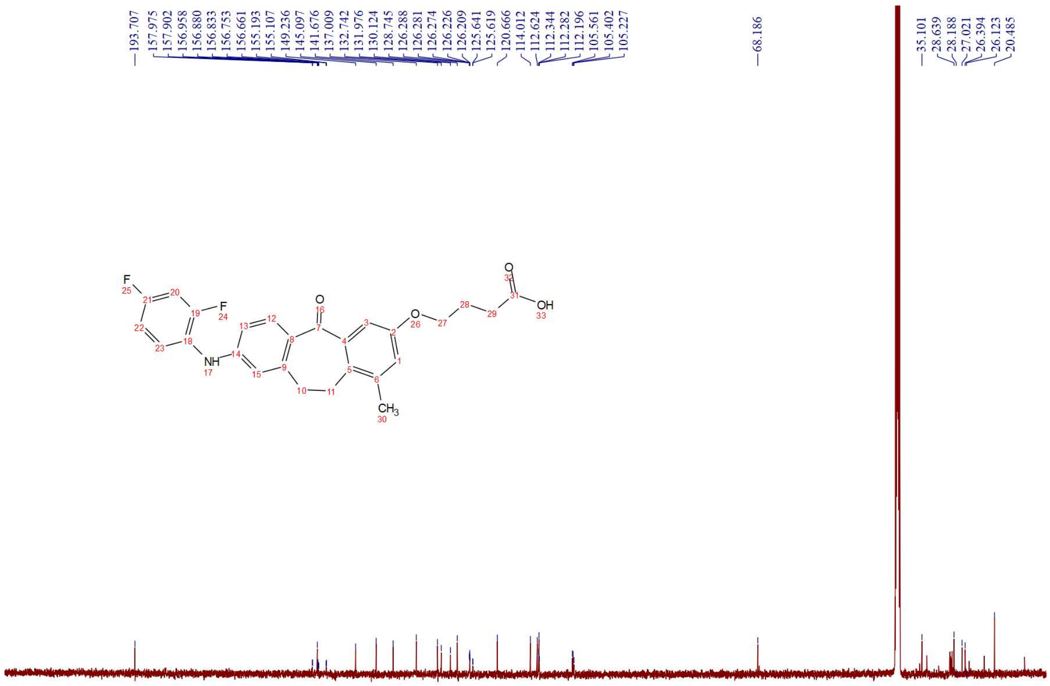
193.707
157.975
157.902
156.958
156.880
156.833
156.753
156.661
155.193
155.107
149.236
145.097
141.676
137.009
132.742
131.976
130.124
128.745
126.288
126.281
126.274
126.226
126.209
125.641
125.619
120.666
114.012
112.624
112.344
112.282
112.196
105.561
105.402
105.227
68.186
35.101
28.639
28.188
27.021
26.394
26.123
20.485
F
NH
O
OH
CH3

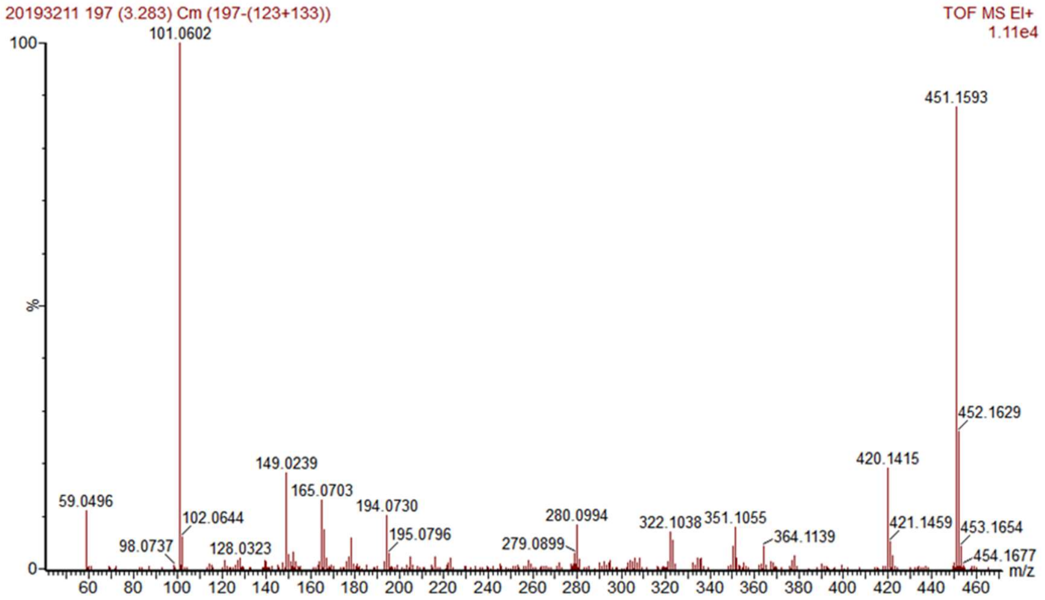

(J) ^{1}H NMR, ^{13}C NMR and HRMS (EI) spectrums of Compound **13b**.

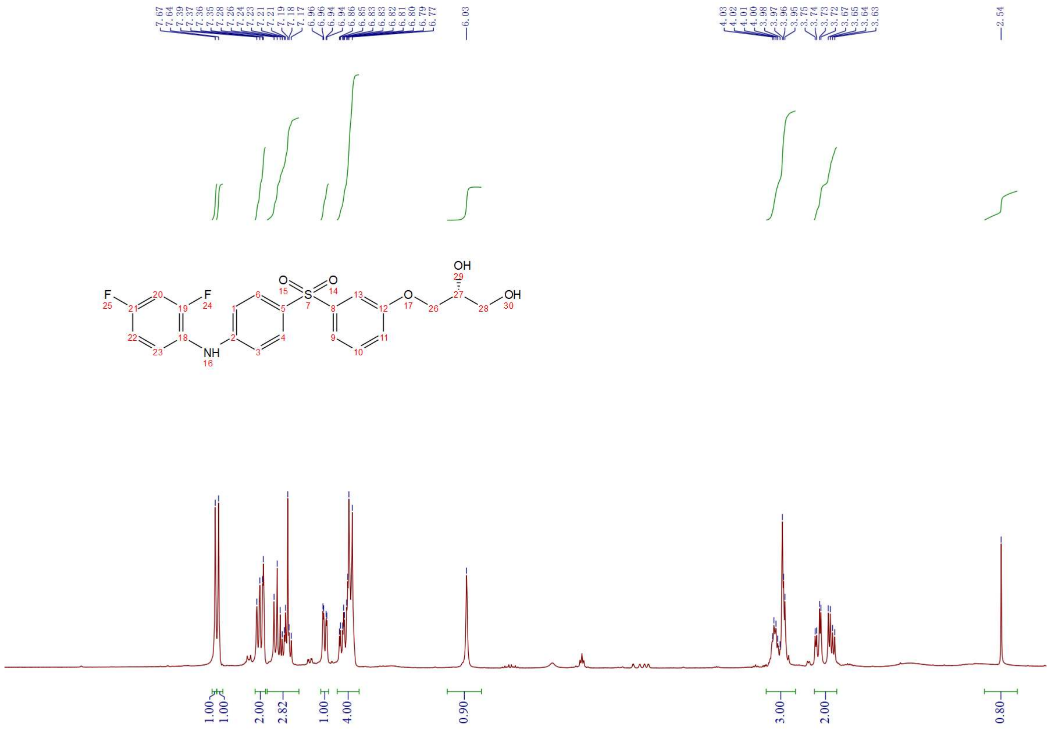

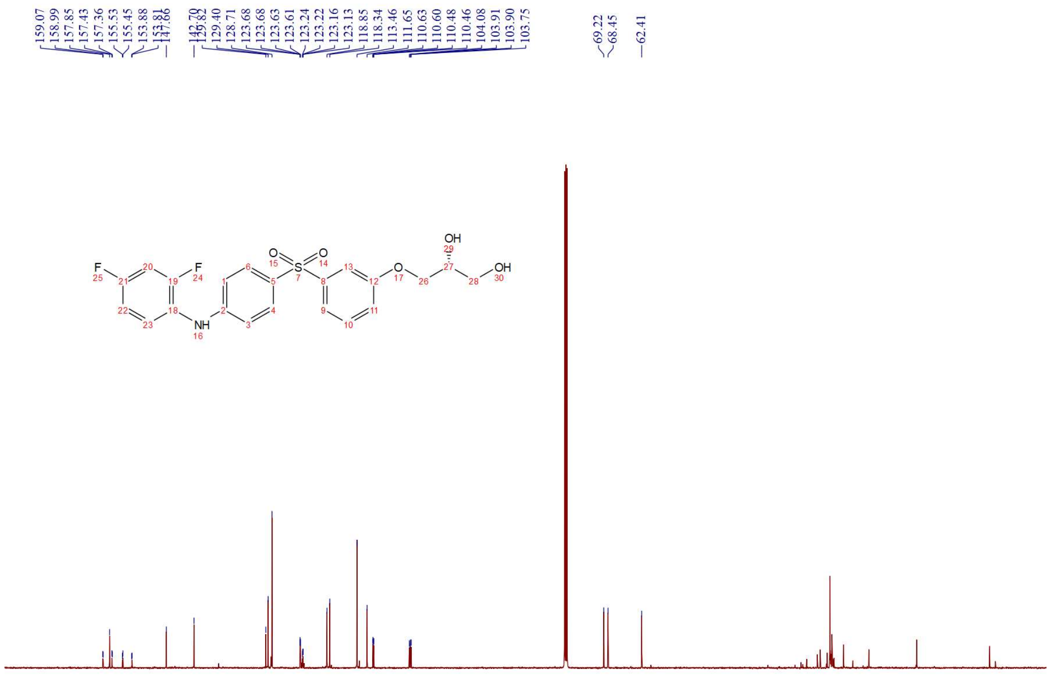

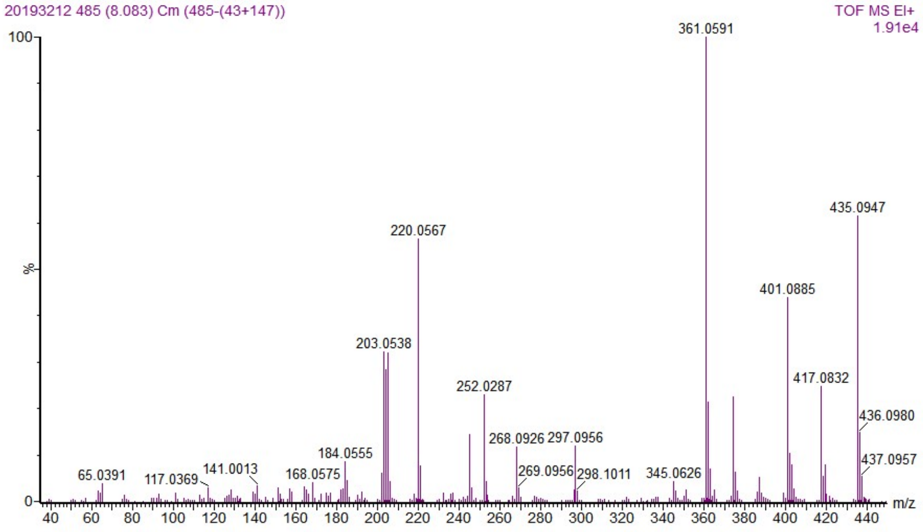

(K) ^{1}H NMR, ^{13}C NMR and HRMS (EI) spectrums of Compound **23b**.

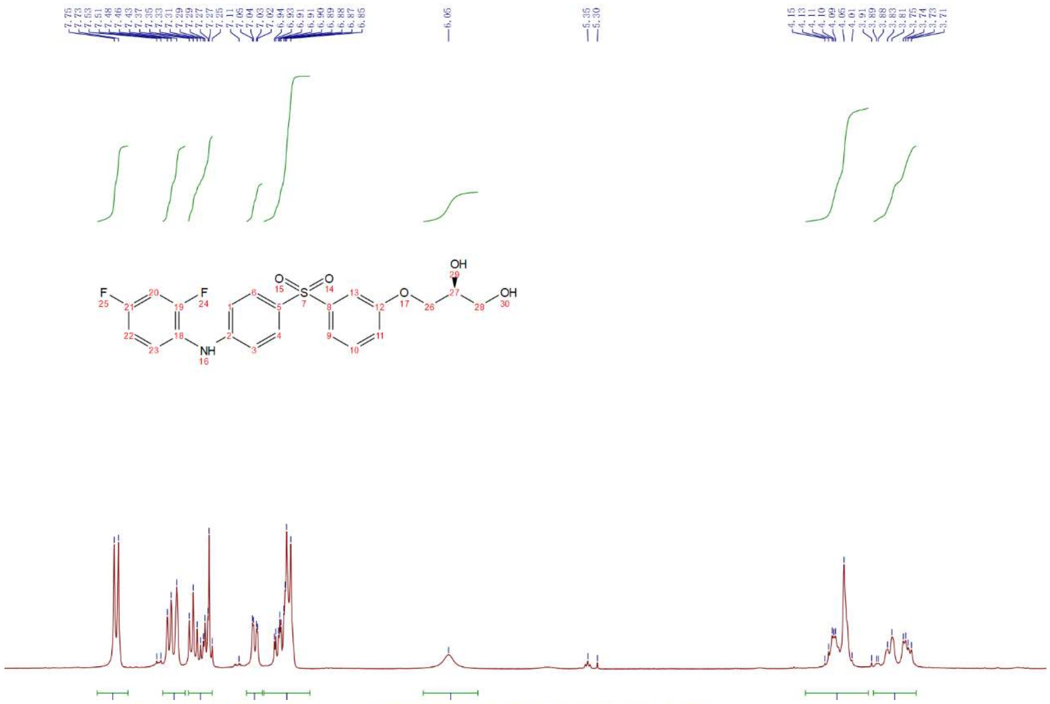
7.75 7.73 7.53 7.51 7.48 7.46 7.43 7.37 7.35 7.33 7.31 7.29 7.29 7.27 7.27 7.25 7.11 7.05 7.04 7.03 7.02 6.94 6.93 6.91 6.91 6.90 6.89 6.88 6.87 6.85
6.05
5.35 5.30
4.15 4.13 4.11 4.10 4.09 4.05 4.01 3.91 3.89 3.88 3.83 3.81 3.75 3.74 3.73 3.71
F
F
NH
O
S
O
O
OH
OH

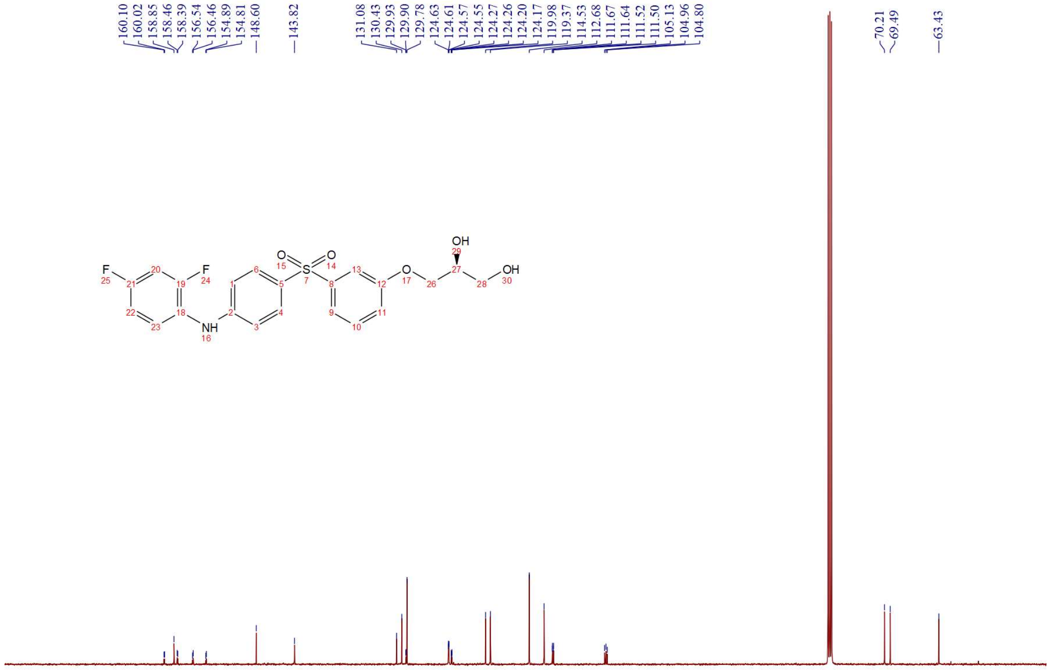
160.10 160.02 158.85 158.46 158.39 156.54 156.46 154.89 154.81
148.60
143.82
131.08 130.43 129.93 129.90 129.78 124.63 124.61 124.57 124.55 124.27 124.26 124.20 124.17 119.98 119.37 114.53 112.68 111.67 111.64 111.52 111.50 105.13 104.96 104.80
70.21 69.49
63.43
F
F
NH
O
S
O
O
OH
OH

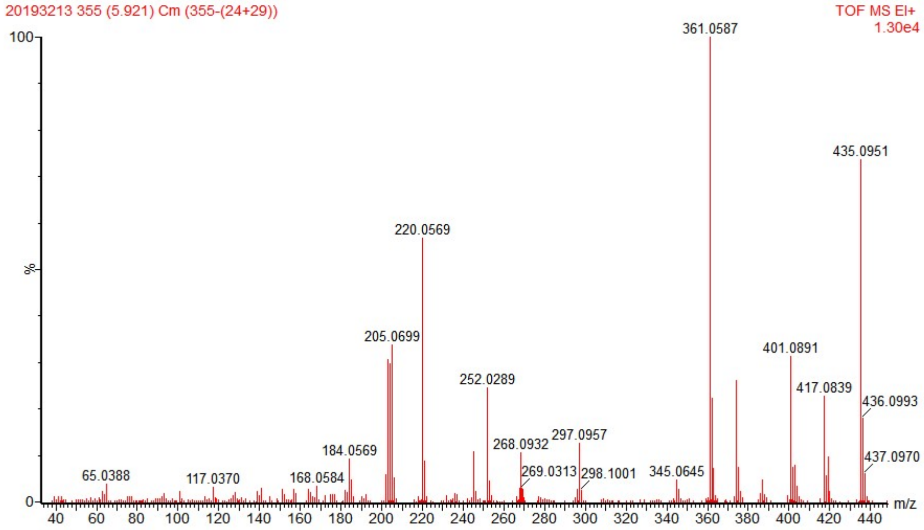

(L) ^{1}H NMR, ^{13}C NMR and HRMS (EI) spectrums of Compound **23c**.

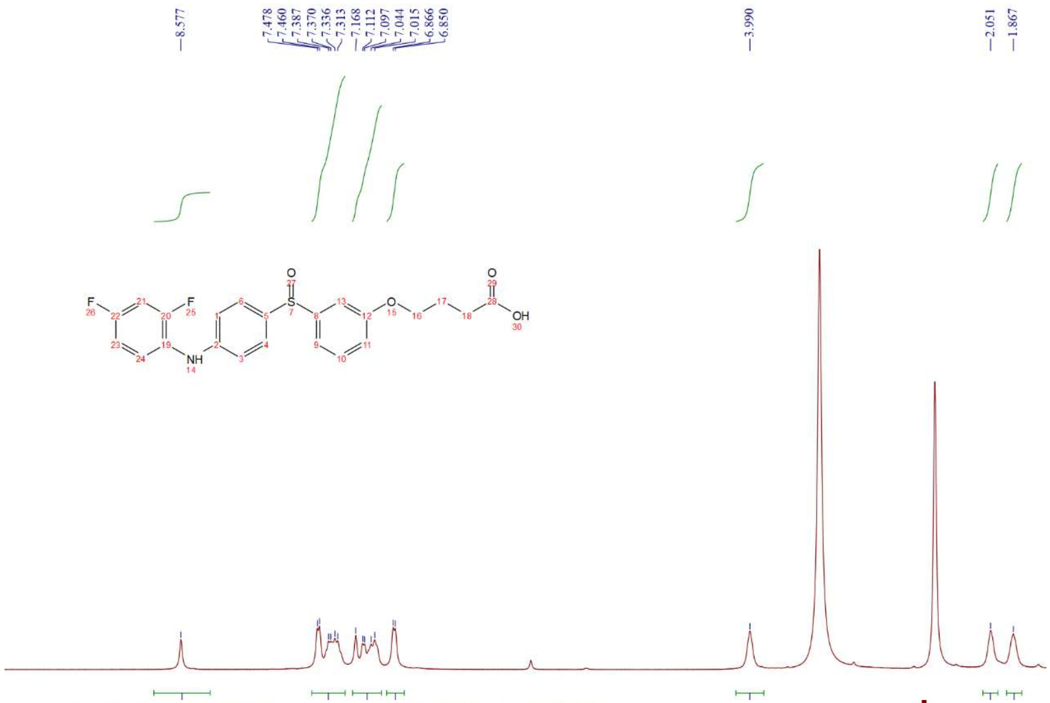
8.577
7.478
7.460
7.387
7.370
7.336
7.313
7.168
7.112
7.097
7.044
7.015
6.866
6.850
3.990
2.051
1.867
F
26
F
25
NH
14
S
O
27
7
O
15
O
29
OH
30

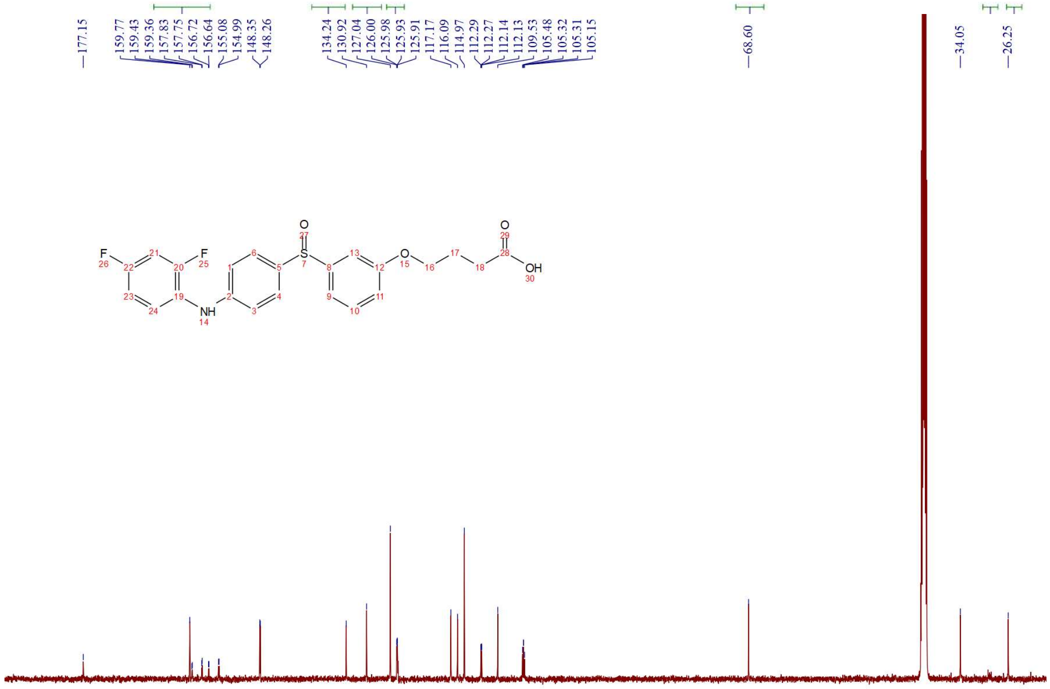
177.15
159.77
159.43
159.36
157.83
157.75
156.72
156.64
155.08
154.99
148.35
148.26
134.24
130.92
127.04
126.00
125.98
125.93
125.91
117.17
116.09
114.97
112.29
112.27
112.14
112.13
109.53
105.48
105.32
105.31
105.15
68.60
34.05
26.25
F
26
F
25
NH
14
S
O
27
7
O
15
O
29
OH
30

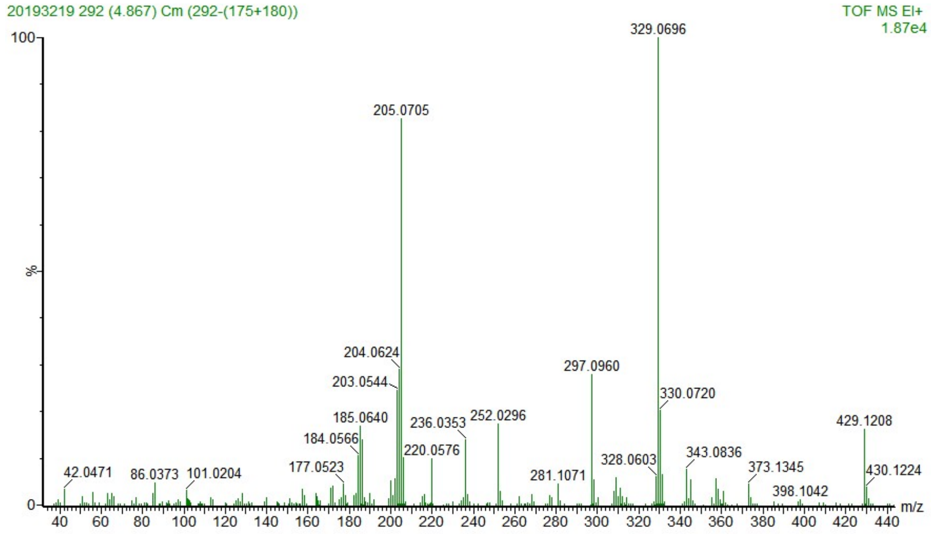

(M) ^{1}H NMR, ^{13}C NMR and HRMS (EI) spectrums of Compound **25**.

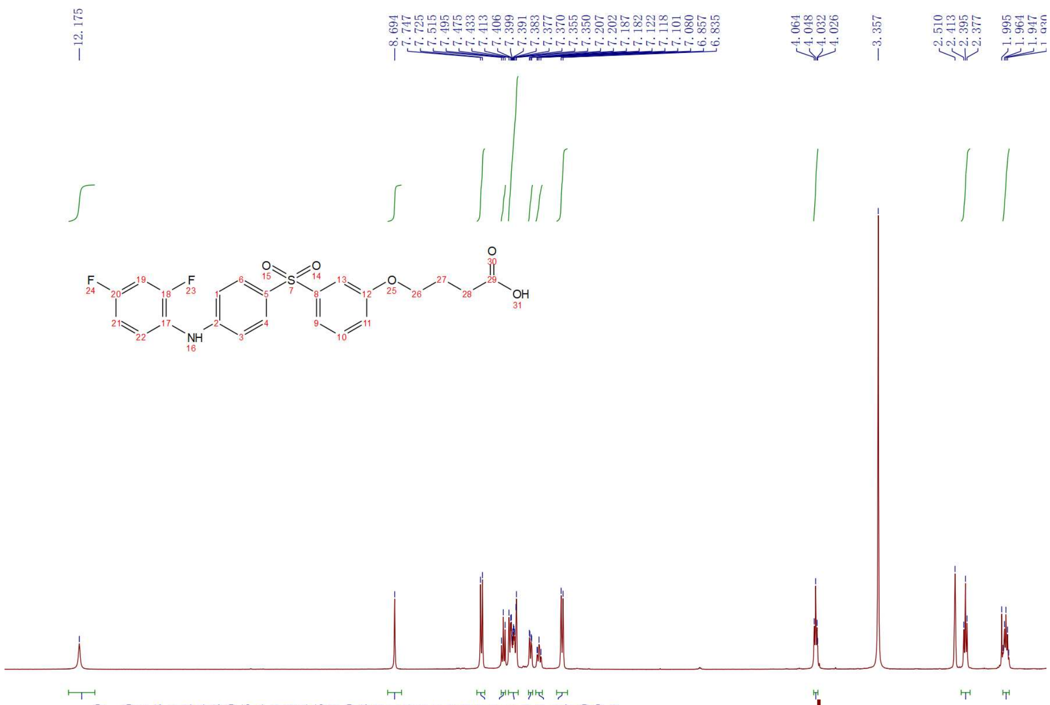
12.175
8.694
7.747
7.725
7.515
7.495
7.475
7.433
7.413
7.406
7.399
7.391
7.383
7.377
7.370
7.355
7.350
7.207
7.202
7.187
7.182
7.122
7.118
7.101
7.080
6.857
6.835
4.064
4.048
4.032
4.026
3.357
2.510
2.413
2.395
2.377
1.995
1.964
1.947

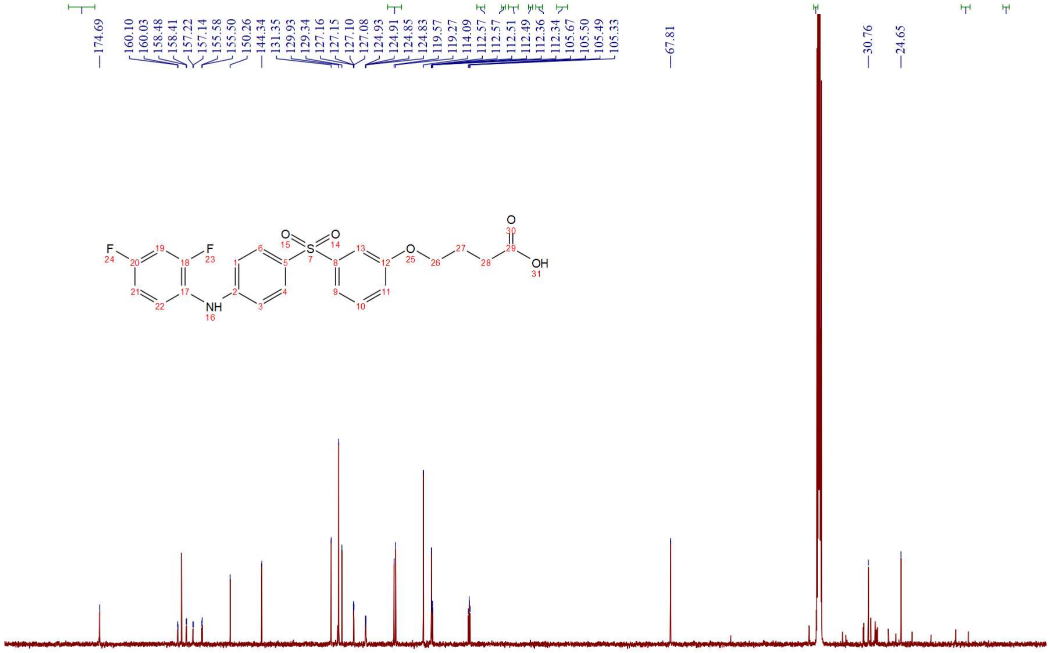
174.69
160.10
160.03
158.48
158.41
157.22
157.14
155.58
155.50
150.26
144.34
131.35
129.93
129.34
127.16
127.15
127.10
127.08
124.93
124.91
124.85
124.83
119.57
119.27
114.09
112.57
112.57
112.51
112.49
112.36
112.34
105.67
105.50
105.49
105.33
67.81
30.76
24.65

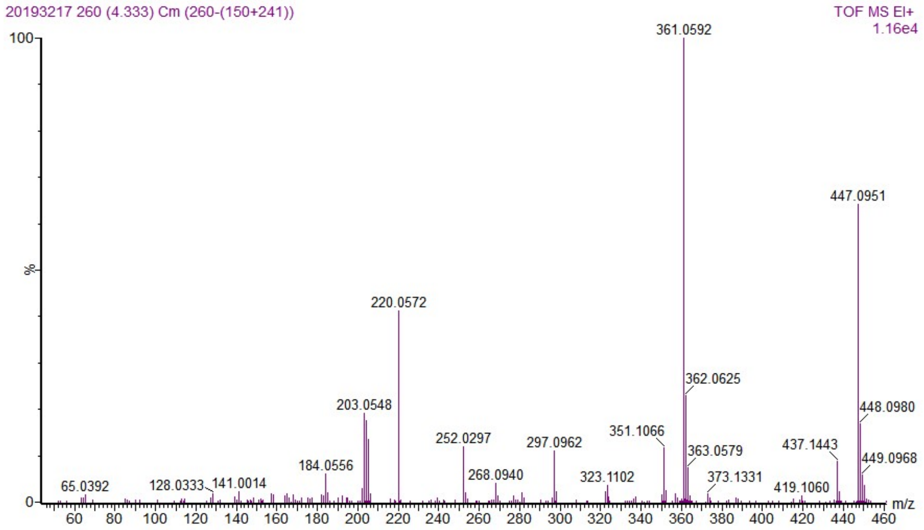

(N) ^{1}H NMR, ^{13}C NMR and HRMS (EI) spectrums of Compound **26**.
